# Tuning the Electronic Properties of Azophosphines as Ligands and Their Application in Base-Free Transfer Hydrogenation Catalysis

**DOI:** 10.1021/acs.organomet.4c00302

**Published:** 2024-09-06

**Authors:** Emma J. Jordan, Ethan D. E. Calder, Bethan L. Greene, Holly V. Adcock, Louise Male, Paul W. Davies, Andrew R. Jupp

**Affiliations:** School of Chemistry, University of Birmingham, Edgbaston, Birmingham B15 2TT, United Kingdom

## Abstract

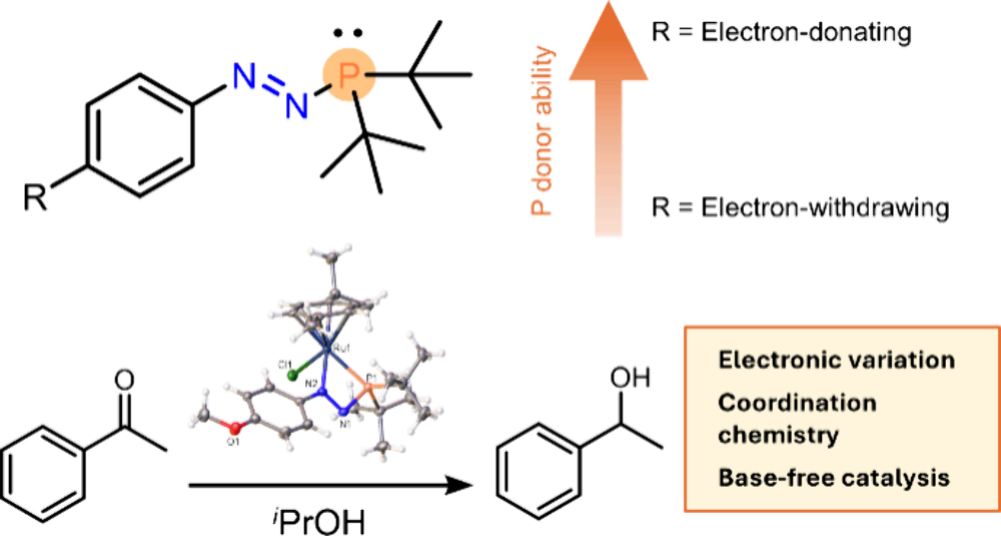

The design and tuning of new ligands is crucial for unlocking new reactivity at transition metal centers. Azophosphines have recently emerged as a new class of 1,3-P,N ligands in ruthenium piano-stool complexes. This work shows that the azophosphine synthesis can tolerate *N*-aryl substituents with strongly electron-donating and electron-withdrawing *para*-R groups and that the nature of this R group can affect the spectroscopic and structural properties of the azophosphines, as measured by NMR spectroscopy, UV–vis spectroscopy, single-crystal X-ray diffraction, and DFT studies. Azophosphines are shown to be relatively weak phosphine donors, as shown by analysis of the ^1^*J*_P–Se_ coupling constants of the corresponding azophosphine selenides, but the donor properties can be fine tuned within this area of chemical space. Monodentate and bidentate Ru–azophosphine complexes were prepared, and their first use as a catalyst was probed. The Ru–azophosphine complexes were found to promote the transfer hydrogenation of acetophenone to 1-phenylethanol without the requirement of a harsh base additive, and the bidentate complex was more active than the monodentate analogue.

## Introduction

**Figure 1:**
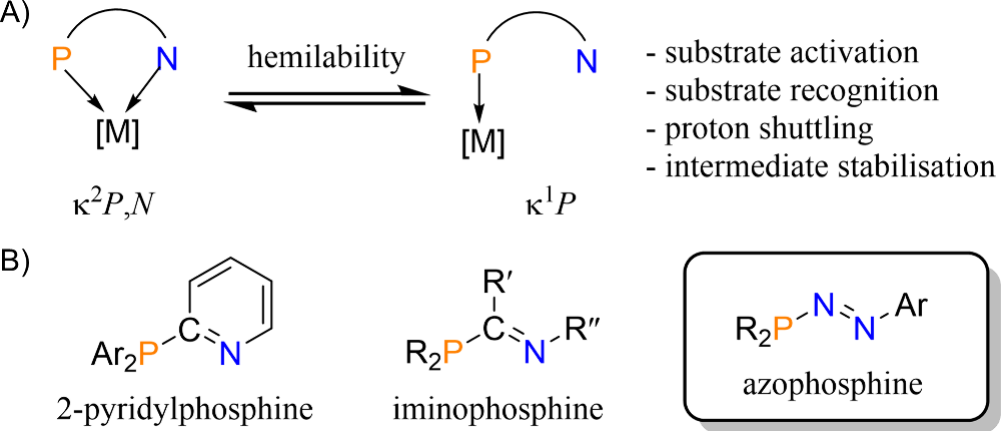
(A) Properties of hybrid P,N ligands; (B) examples of 1,3-P,N ligands.

The use of ligands to control the electronic and steric environment around a metal center is crucial for tuning the properties of metal centers for applications in catalysis, pharmaceutical chemistry, and materials. More sophisticated ligands can also be designed to enable metal–ligand cooperativity, which unlocks reactivity that is not possible at the metal center alone. Hybrid ligands that feature different donor sites can take advantage of specific binding preferences to give control of the coordination chemistry and allow for these cooperative effects such as hemilability, proton shuttling, and substrate recognition and activation (Figure 1A).^1^ 1,3-P,N ligands are a widely studied class of compounds that can bind selectively through the hard N atom, the soft P atom, or both and can bind to one or multiple metal centers.^2−6^ This ligand class is typified by 2-pyridylphosphines and iminophosphines, both of which have a C atom as the bridging center between the P and the N donors (Figure 1B).^2^

Azophosphines (Figure 1B) are heavier analogues of triazenes and are a new member of the family of 1,3-P,N ligands that have a bridging N atom between the donor N and P sites.^7^ A limited number of azophosphines were explored in the 1970s and 1980s,^8−10^ but interest in this class of molecules has been renewed with a series of recent publications exploring their synthesis and reactivity. The Cummins group synthesized a *P*-anthracenyl derivative that could serve as a synthetic equivalent of a phosphaazide^11^ and a small family of azophosphines that could undergo cycloaddition chemistry with alkynes to form N-heterocyclic iminophosphoranes.^12^ In our own work, we have also demonstrated cycloaddition chemistry of azophosphines to form seven-membered diazaphosphepine heterocycles,^13^ and most pertinent to this study, we showed that azophosphines could act as ligands in Ru complexes.^7^ The azophosphines initially bound to the ruthenium in a κ^1^-*P* manner, but the bidentate κ^2^-*P*,*N* binding mode could be cleanly accessed by using a halide-abstracting agent to free up a coordination site on the metal center.

Ru complexes have been widely studied as catalysts for transfer hydrogenation reactions, where an organic H_2_ surrogate is used to bypass the hazards associated with using high pressures of H_2_ gas.^14−16^ Isopropanol has been touted as the ideal H_2_ source, as it is inexpensive, readily available, and highly solubilizing, has an appropriate boiling point, and generates acetone as a byproduct.^17^ The Ru-catalyzed transfer hydrogenation of ketones is a powerful reaction to produce alcohols, and if a chiral catalyst is used, this can be done in an asymmetric manner. The conversion of acetophenone into 1-phenylethanol using isopropanol as the H_2_ source has been catalyzed by a range of Ru-based catalysts,^18−25^ and in all but one case an alkali metal base is required as an additive for catalysis to occur. The only example of a Ru catalyst for this reaction that does not require addition of a strong base is an isolated ruthenium hydride, and in this case, adding a large excess of potassium *tert*-butoxide had no effect.^26^ The ability to catalyze this reaction without the requirement for harsh bases is highly desirable.

In this work, we broaden the scope of azophosphines by systematically varying the electronic properties of the *N*-aryl substituent and explore how this affects their structural and spectroscopic characteristics. The azophosphine-selenides are synthesized to probe how these electronic variations affect the P-donor properties of the azophosphines. The azophosphines are subsequently assessed as ligands in ruthenium piano-stool complexes. Finally, we demonstrate that these Ru complexes are catalytically active in the transfer hydrogenation of acetophenone without the need for a strong base additive.

## Results and Discussion

### Synthesis and Characterization

**Scheme 1:**
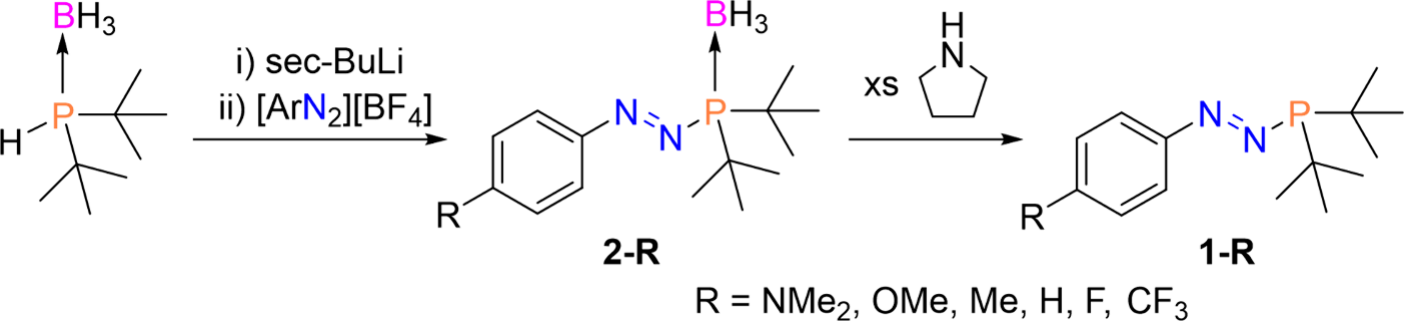
Synthesis of Azophosphines 1-R via the Corresponding Azophosphine-Boranes **2-R**

To probe how varying the electronic properties of the azophosphines would affect their overall structure and ligand ability, we targeted a series of azophosphines that varied at the *N*-aryl position. The para substituent of the aryl group was changed from strongly electron donating to electron withdrawing while maintaining the same substituents on the phosphorus in all cases to enable simple comparison. Using our general synthetic route to azophosphines (**1-R**)^7^ via the corresponding azophosphine-borane (**2-R**), we obtained azophosphine-boranes **2-R** (R = NMe_2_, OMe, Me, H, F, CF_3_) as brightly colored red-orange solids (Scheme 1, isolated yields = 46–84%). The borane protecting groups in **2-R** were removed to afford the corresponding azophosphines **1-R** as darker colored solids or oils (isolated yields = 50–76%). Note that **1-NMe**_**2**_ and **2-NMe**_**2**_ were previously reported in our earlier communication,^7^ but all other molecules presented herein are novel.

**Figure 2:**
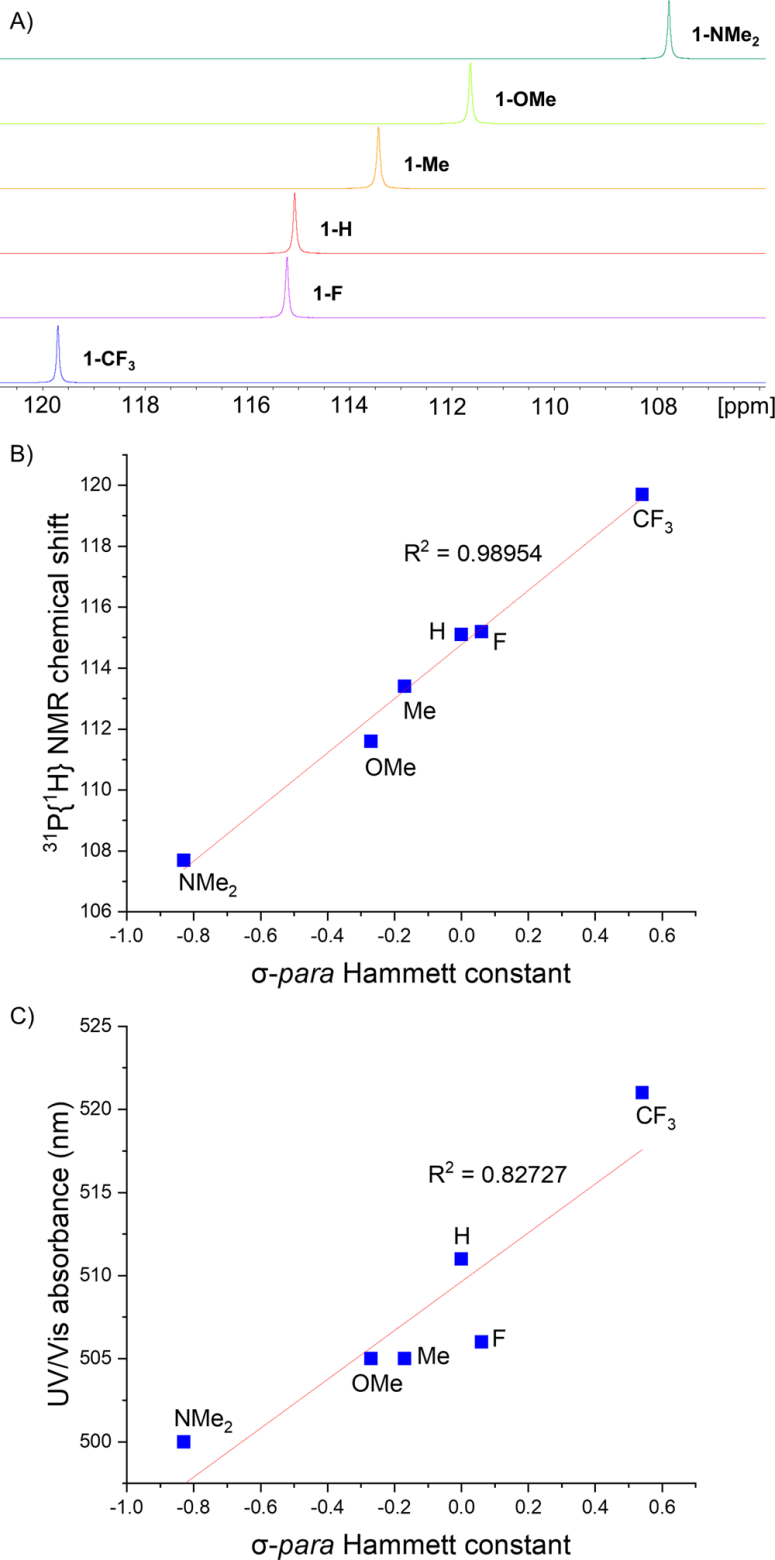
(A) Stacked ^31^P{^1^H} NMR spectra of **1-R**. (B) Plot of the ^31^P{^1^H} NMR chemical shift (δ) against the σ-para Hammett constant for **1-R**. (C) Plot of the λ_max_ (nm) of UV–vis absorbance between 500 and 521 nm against the σ-para Hammett constant for **1-R**.

The electronic effect of modulating R can be seen spectroscopically in the ^31^P{^1^H} NMR spectra and UV–vis spectra of **1-R**. The ^31^P{^1^H} chemical shift of **1-R** becomes gradually more deshielded as the R group becomes more electron withdrawing (Figure 2A), which demonstrates that although the R group is distant from the phosphorus center, it does still have a marked effect on its properties. This trend is evident in a plot of the ^31^P NMR chemical shifts of **1-R** against the corresponding σ-para Hammett constants of R, which shows a positive linear trend with an *R*^2^ value of close to unity (Figure 2B).

The colors of **1-R** are various shades of orange and red. The UV–vis spectra of each azophosphine show a strong absorbance band in the UV region between 350 and 388 nm and a much weaker absorbance band between 500 and 521 nm that is responsible for the color of the compounds. The UV–vis spectra of **1-R** show only minor differences in absorption as the R group is varied. Overall, absorbance is red shifted moving from **1-NMe**_**2**_ to **1-CF**_**3**_; however, **1-F** is blue shifted relative to **1-H** due to the mesomeric effect of F. This is summarized in the plot of the UV–vis absorbance of **1-R** against the corresponding σ-para Hammett constants of R (Figure 2C), which shows an overall linear trend with an *R*^2^ value of 0.82727.

**Figure 3:**
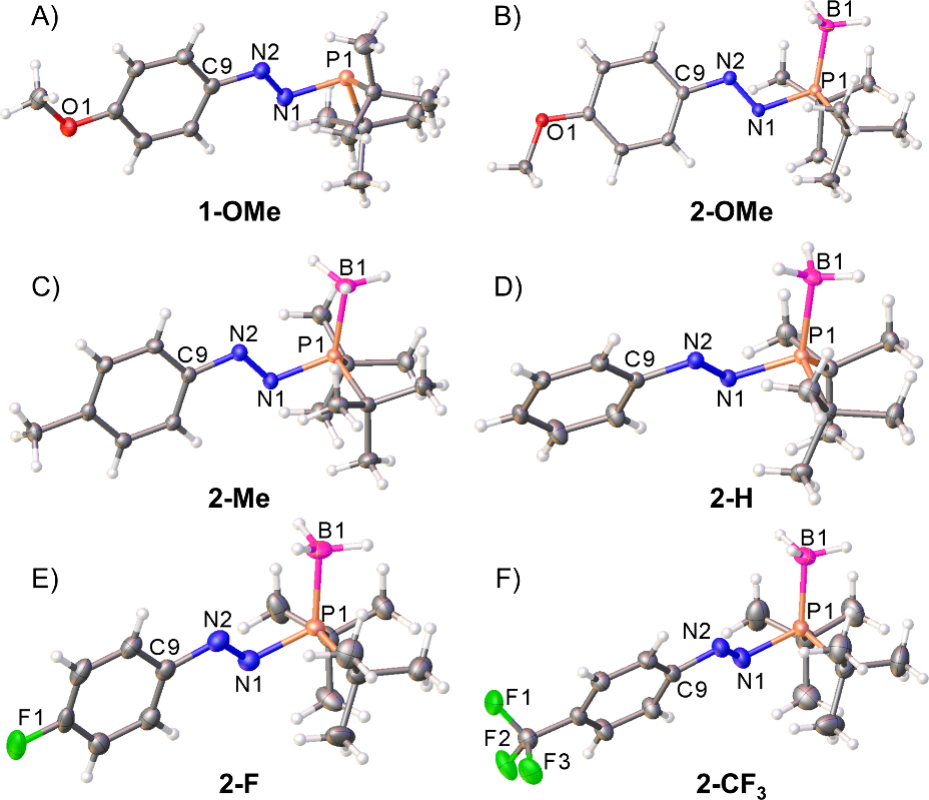
Single-crystal structures of **1-OMe** (A), **2-OMe** (B), **2-Me** (C), **2-H** (D), **2-F** (E), and **2-CF**_**3**_ (F). The structures of **2-F** and **2-CF**_**3**_ contain four and two crystallographically independent molecules, respectively. Only one molecule is shown for clarity. Thermal ellipsoids were drawn at the 50% probability level.^27^

The structural variations in the azophosphines and the azophosphine-boranes were also explored. Azophosphine **1-OMe** and all six azophosphine-boranes **2-R** were characterized by single-crystal X-ray diffraction (SXRD) (Figure 3). Note the structures of **1-NMe**_**2**_ and **2-NMe**_**2**_ were previously reported^7^ and are not reproduced in Figure 3, but the bond metric data are used here for comparison purposes. Selected bond distances, bond angles, and torsion angles for crystallographically characterized azophosphines **1-NMe**_**2**_ and **1-OMe** and azophosphine-boranes **2-R** are tabulated in Table 1.

**Table 1:** Selected Bond Distances (Angstroms), Bond angles (degrees), and Torsion Angles (degrees) of Crystallographically Characterized Azophosphines (**1-R**) and Azophosphine-Boranes (**2-R**)^a^^7^

	C9–N2	N1–N2	N1–P1
**1-NMe**_**2**_^b^	1.424(3)	1.258(3)	1.7494(18)
			1.7655(19)
**1-OMe**	1.4333(19)	1.2560(17)	1.7660(13)
**2-NMe**_**2**_^b^	1.4047(18)	1.2681(17)	1.7380(12)
**2-OMe**	1.4238(15)	1.2561(14)	1.7576(10)
**2-Me**	1.4318(17)	1.2475(16)	1.7519(12)
**2-H**	1.4329(14)	1.2516(14)	1.7575(10)
**2-F**	1.441(4)	1.233(4)	1.763(3)
**2-CF**_**3**_	1.443(2)	1.235(2)	1.7552(17)

	P1–N1–N2	C10–C9–N1–N2
**1-NMe**_**2**_^b^	119.34(14)	22.9(3)
	115.16(15)	15.9(2)
**1-OMe**	113.81(11)	3.4(2)
**2-NMe**_**2**_^b^	114.33(10)	4.3(2)
		0.3(2)
**2-OMe**	113.37(8)	6.24(16)
**2-Me**	114.28(9)	3.79(19)
**2-H**	113.04(8)	1.30(16)
**2-F**	113.4(3)	4.4(5)
**2-CF**_**3**_	115.20(14)	1.9(3)
		12.7(3)

a For structures containing >1 crystallographically independent molecules: one representative value is provided if there is no statistically significant difference (3σ); values for each molecule are provided if there is a statistical difference.

b Crystal structures previously reported, but selected bond distances and angles provided for comparison.

The structure of azophosphine **1-OMe** has N1–N2 and N1–P1 bond lengths of 1.2560(17) and 1.7660(13) Å, respectively, and is analogous to the previously reported **1-NMe**_**2**_.^7^ The pyramidal geometry at phosphorus for **1-OMe** is reflected in the sum of the angles around P1 (307.9°), which highlights that the lone pair on phosphorus will be available for further reactivity and coordination chemistry.

The fact that we were able to obtain crystal structures for all six azophosphine-boranes **2-R** enabled us to assess systematic trends as the electronic properties of the R group are varied (Table 1). In general, the N1–N2 bond distance increases and the C9–N2 bond distance decreases as the R group becomes more electron donating, as expected with the increased donation of electron density from the *N*-aryl substituent into the N=N π* orbital. This effect can be seen most clearly by comparing the two extreme azophosphine-boranes in terms of electronic properties: **2-NMe**_**2**_ (C9–N2, 1.4047(18) Å; N1–N2, 1.2681(17) Å) and **2-CF**_**3**_ (C9–N2, 1.443(2) Å; N1–N2, 1.235(2) Å). The percentage change in the P–N bond distances is much smaller across the series and not in a specific order, which is logical because the phosphine lone pair is forming a dative bond to the borane moiety and therefore cannot have a significant interaction with the neighboring N=N π* orbital. The P1–N1–N2 bond angles also do not sequentially increase/decrease moving through the library, although there is a small degree of variation. The most significant differences in torsion angle within the series of **2-R** are observed within **2-NMe**_**2**_ and **2-CF**_**3**_, which contain more than one crystallographically independent molecule; thus, these differences can be assigned to crystal packing effects.

**Figure 4:**
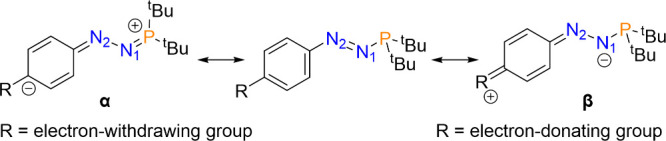
Key resonance structures of azophosphines, where the para substituent is varied from an electron-withdrawing to an electron-donating group.

To explore the electronic structure of azophosphines **1-R** in more detail, density functional theory (DFT) calculations and natural bond orbital (NBO) analyses were carried out (see SI for details). The N–N bond distances in this series are all in a very narrow range of 1.236–1.239 Å (values in Table S3), which implies there is an approximately equal degree of delocalization of electron density into the N=N π* orbital across the series. However, the source of this donated electron density varies as a function of the nature of the R group, as shown by the two resonance structures α and β (Figure 4). Natural population analysis (NPA) shows an increasingly electron-poor phosphorus center as more electron-withdrawing substituents are used. This is consistent with a higher contribution from resonance α as the phosphorus lone pair is increasingly delocalized into the N=N π* orbital and extended π system. Conversely, with more electron-donating substituents, the NPA value for N1 becomes more negative, consistent with a higher contribution from resonance β as more electron density resides on the formally anionic N1 center. The NPA values are further supported by examination of the donor/acceptor interactions, which show larger donations from the neighboring aryl π orbital into the N=N π* orbital with electron-donating substituents and increasing donation of the phosphorus lone pair into the N=N π* orbital with electron-withdrawing substituents (see Table S5 for all values). The similarity of the lengths of the N=N bonds across the series is reflective of both resonance forms α and β imparting similar effects on the bond. These data underline the effects of both competing resonances on azophosphines and suggest that control over the substituent may have an important role on the applications of azophosphines as ligands, with electron-donating substituents yielding a more electron-rich phosphorus center.

### Azophosphine-Selenides

**Scheme 2:**
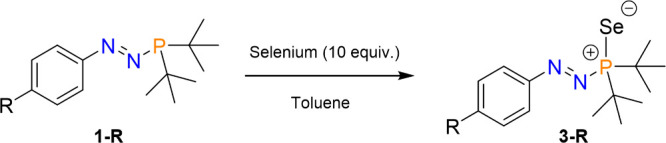
Synthesis of Azophosphine-Selenides **3-R**

To gain more insight into how varying the electronics of the *N*-aryl group affect the σ-donor character of the phosphorus center of the azophosphine ligands, we investigated the ^31^P–^77^Se spin–spin coupling constants (^1^*J*_P–Se_) of the corresponding azophosphine selenides (**3-R**; Scheme 2) by ^31^P{^1^H} NMR spectroscopy (^77^Se: 7.6% abundant, *I* = 1/2). This approach is well established in the literature^28−31^ as an alternative to the more traditional measurement of the Tolman electronic parameter.^30,32,33^ Providing the steric bulk surrounding the phosphorus atom remains consistent,^28^ the value of the ^1^*J*_P–Se_ coupling constant is dependent on the electronic nature of the substituents at P and, as governed by Bent’s rule and the Fermi-contact interactions of the s orbitals of P and Se, increases with increasingly electron-withdrawing substituents. The magnitude of ^1^*J*_P–Se_ therefore inversely corresponds to the relative σ-donor capability of a homologous series of P-based ligands.

The azophosphine-selenides were prepared by addition of an excess of gray selenium to a toluene solution of the azophosphine **1-R** (Scheme 2). Full conversion to the azophosphine-selenide was confirmed by ^31^P{^1^H} NMR spectroscopy. A similar trend in the ^31^P chemical shift to that of **1-R** is observed for the azophosphine-selenides, increasing in ppm from **3-NMe**_**2**_ to **3-CF**_**3**_ (Table 2). The value of the ^1^*J*_P–Se_ coupling constant increases from **3-NMe**_**2**_ to **3-CF**_**3**_, which demonstrates that the phosphorus centers in the azophosphine ligands **1-R** become a poorer σ donor as R gets increasingly electron withdrawing, as expected.

**Table 2:** Chemical Shifts (ppm) of **1-R** and **3-R** and ^1^*J*_P–Se_ Coupling Constants (Hertz) of **3-R** Determined by ^31^P{^1^H} NMR Spectroscopy in Toluene-*d*_8_

R	δ/ppm (**1-R**)	δ/ppm (**3-R**)	^1^*J*_P–Se_/Hz (**3-R**)
NMe_2_	107.8	108.8	794
OMe	111.7	112.0	801
Me	113.4	113.3	804
H	115.1	114.1	806
F	115.2	114.4	806
CF_3_	119.7	116.4	810

To put these values in perspective against established phosphines that have a similar steric profile around the phosphorus center, the ^1^*J*_P–Se_ values of the selenides of P^*t*^Bu_3_ (709 Hz in C_6_D_6_ or 686 Hz in CDCl_3_) and JohnPhos (2-(di-*tert*-butylphosphino)biphenyl, a ligand used with high success in Pd-catalyzed cross-coupling reactions; 735 Hz in CDCl_3_) are significantly lower than those of azophosphines **1-R**, and the azophosphines are therefore much poorer donors.^30,31,34^ The influence of the electron-withdrawing azo group on the donor ability of **3-R** can be seen by comparing **3-H** (806 Hz) and **3-CF**_**3**_ (810 Hz) with the selenides of the analogous PhP^*t*^Bu_2_ (708 Hz in CDCl_3_) and (*p*-(CF_3_)C_6_H_4_)P^*t*^Bu_2_ (720 Hz in CDCl_3_), respectively.^31^**3-H** and **3-CF**_**3**_ are both approximately 100 Hz greater in ^1^*J*_P–Se_ value than their nonazo analogues. These data show that in general azophosphines are relatively weak phosphine donors and that within this chemical space it is possible to fine tune the donor properties by varying the R group.

### Coordination Chemistry

**Scheme 3:**
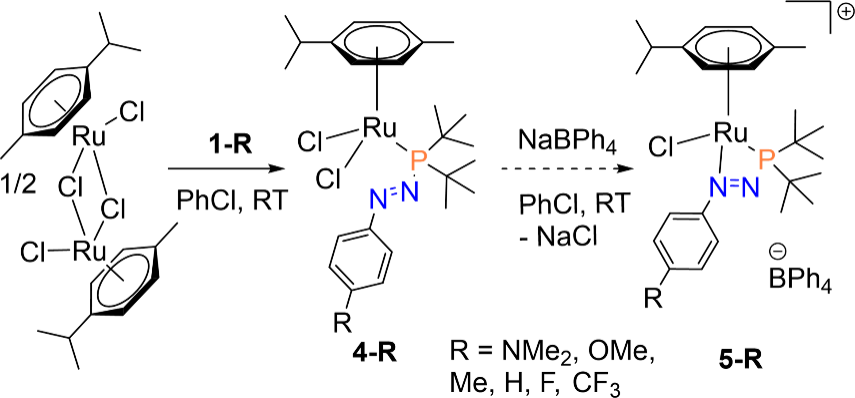
General Synthesis Used To Evaluate the κ^2^*P*,*N* Coordinative Ability of Azophosphines.

**Figure 5:**
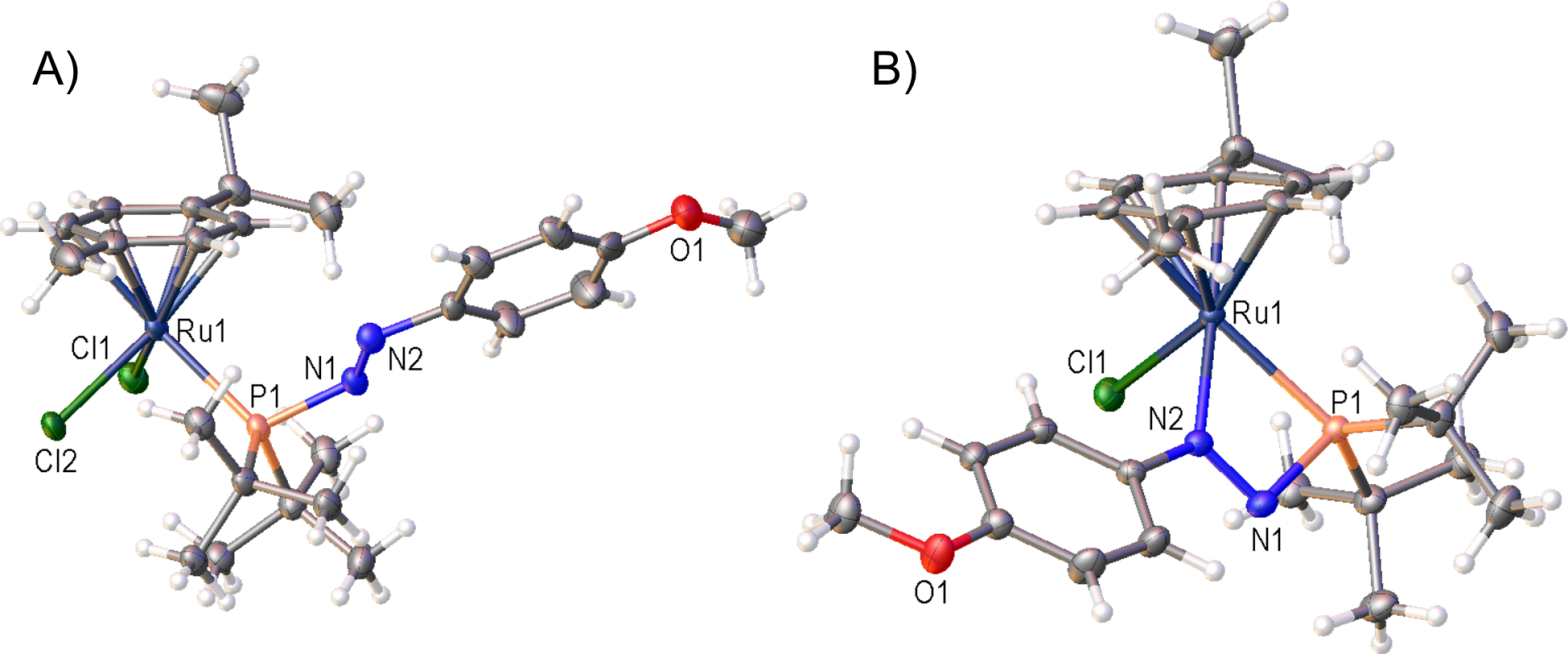
Single-crystal structures of **4-OMe** (A) and the cation in **5-OMe** with the [BPh_4_]^−^ counteranion omitted for clarity (B). Selected bond distances (Angstroms) and angles (degrees). **4-OMe**: P1–Ru1 2.4078(13), P1–N1 1.751(4), N1–N2 1.257(6), P1–N1–N2 118.7(4). **5-OMe**: P1–Ru1 2.3933(5), P1–N1 1.7758(16), N1–N2 1.283(2), P1–N1–N2 99.18(11), P1–Ru1–N2 62.92(4). Thermal ellipsoids were drawn at the 50% probability level.^27^

We previously reported the exclusive κ^1^*P* coordination of MesN_2_P^*t*^Bu_2_ to a chloro-Ru(*p*-cymene) framework and how κ^2^*P,N* coordination could be achieved by using an azophosphine with a smaller steric profile at the ortho positions flanking the terminal nitrogen (**1-NMe**_**2**_) and switching the arene on Ru to benzene.^7^ Continuing our study of the coordination chemistry of azophosphines, we were keen to learn whether fine tuning of their electronics, in addition to sterics, could also affect κ^2^*P,N* coordination. Following the same synthesis (Scheme 3), reaction of **1-OMe** with 0.5 equiv of the [Ru(*p*-cymene)Cl_2_]_2_ dimer gave full conversion from 111.7 to 115.2 ppm in the ^31^P{^1^H} NMR spectrum, indicative of κ^1^*P* coordination. This was corroborated by SXRD and showed the complex has the composition Ru(*p*-cymene)(κ^1^*P*-(**1-OMe**))Cl_2_ (**4-OMe**) (Figure 5A). Addition of NaBPh_4_ to the chlorobenzene solution of **4-OMe** gave a white precipitate (NaCl), and filtration, removal of solvent, and washing with hexane gave **5-OMe** as a brown precipitate in 71% isolated yield. The ^31^P{^1^H} NMR spectrum showed a clean, upfield shift to 67.4 ppm, indicative of κ^2^*P,N* coordination. Again, this was confirmed by SXRD, which showed the bidentate nature of the ligand in the complex [Ru(*p*-cymene)(κ^2^*P,N*-(**1-OMe**))Cl][BPh_4_] (**5-OMe**) (Figure 5B). Pleasingly, this demonstrated that κ^2^*P,N* coordination of **1-OMe** was compatible with the bulkier η^6^-(*p*-cymene) on the Ru center and did not require the switch to the η^6^-benzene moiety.

**Figure 6:**
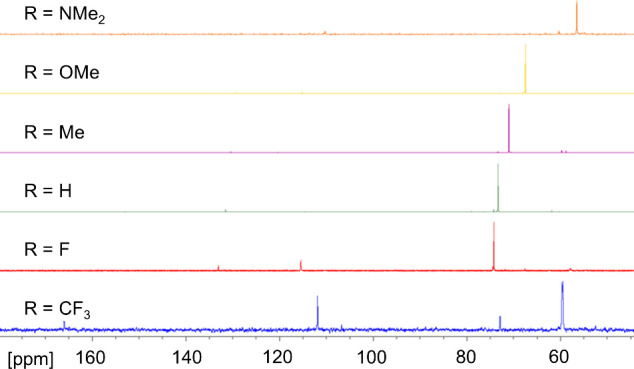
Crude ^31^P{^1^H} NMR spectra of products from the reaction in Scheme 3, in most cases showing formation of **5-R**.

To test how modulating the electronics of **1-R** affected the ability of the azophosphine to act as a competent κ^2^*P,N* ligand, we probed the formation of the bidentate complex with the increasingly electron-poor azophosphines. To chlorobenzene solutions of each azophosphine **1-R** was added 0.5 equiv of the [Ru(*p*-cymene)Cl_2_] dimer. Without isolation of the corresponding Ru(*p*-cymene)(κ^1^*P*-(**1-R**))Cl_2_ (**4-R**) products, NaBPh_4_ was added to initiate κ^2^*P,N* coordination to attempt to access the respective complex [Ru(*p*-cymene)(κ^2^*P,N*-(**1-R**))Cl][BPh_4_] salts (**5-R**), as assessed by ^31^P{^1^H} NMR spectroscopy without any further purification (Figure 6). These experiments show that the electron-rich azophosphine **1-OMe** affords **5-OMe** in quantitative yields under these conditions (as measured by ^31^P{^1^H} NMR spectroscopy). **1-Me** and **1-H** formed predominantly **5-Me** (71.5 ppm) and **5-H** (73.6 ppm), respectively; however, the crude spectra included trace signals for **1-Me** (113.4 ppm), **1-H** (115.1 ppm), **4-Me** (116.5 ppm), and a trace unknown impurity (R = Me, 130.1 ppm; R = H 131.6) (Figure 6). **1-F** also formed mostly **5-F** (74.3 ppm); however, the crude spectrum showed signals associated with **1-F** (115.4 ppm) and an unknown impurity (133.3 ppm) of greater intensity than when R = Me or H. **1-CF**_**3**_ was shown not be an effective ligand for this transformation. It is possible that a small amount of **5-CF**_**3**_ was formed (tentatively assigned as 72.9 ppm), but the crude spectrum showed two main signals at 59.8 and 112.1 ppm and a smaller signal at 165.7 ppm, none of which can be assigned to **1-CF**_**3**_ (119.7 ppm) or **4-CF**_**3**_ (121.6 ppm). These results show that subtle differences to the electronics of the system can have major implications on the ligand properties of azophosphines, which is supported by the relatively minor deviations of the ^1^*J*_P–Se_ coupling constants of the phosphine-selenide compounds **3-R**.

**Figure 7:**
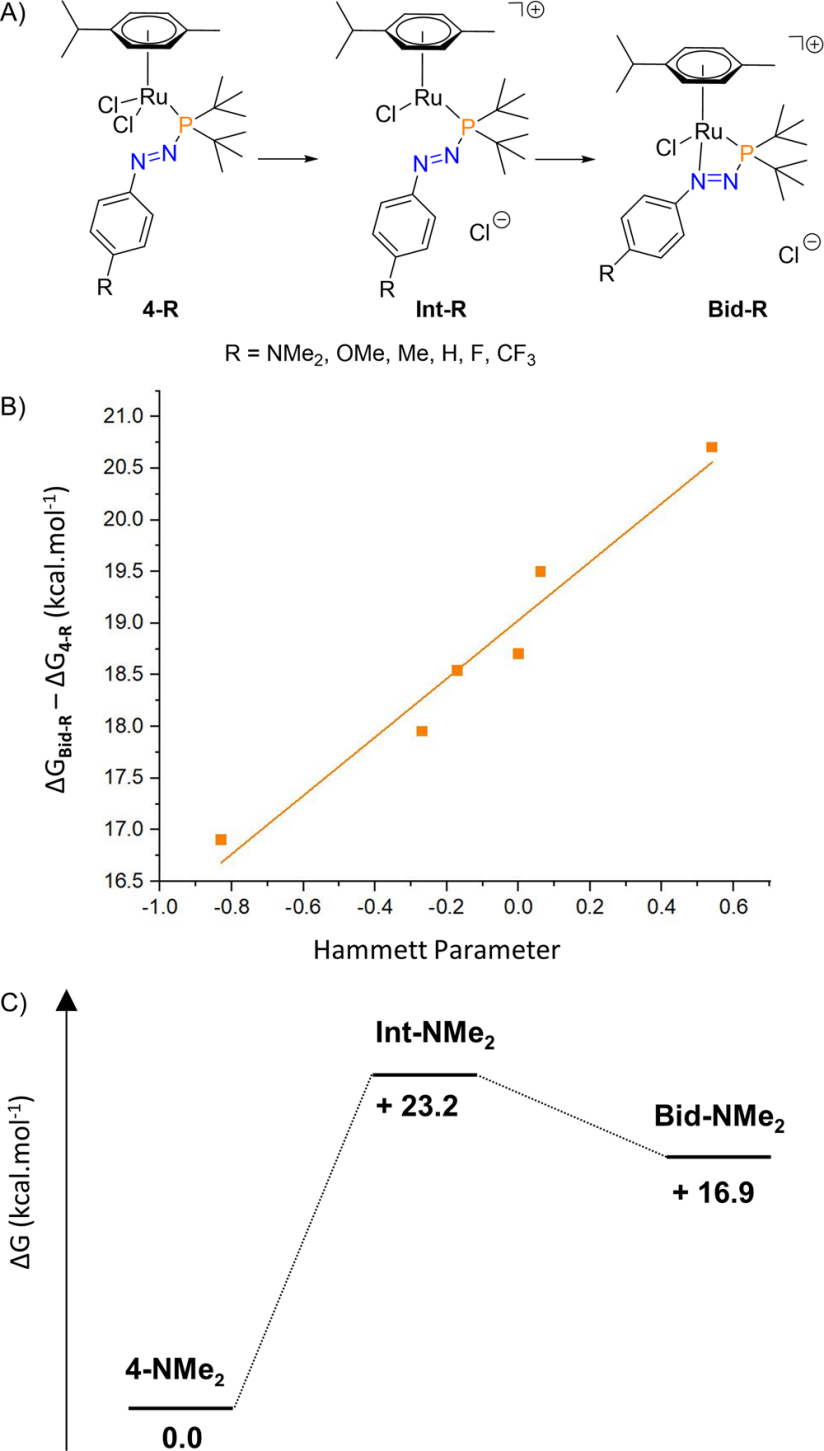
(A) Ru complexes analyzed computationally. (B) Energy difference between **4-R** and **Bid-R** plotted against the Hammett parameter of the para substituent. (C) Reaction coordinate for the conversion of **4-NMe**_**2**_ to **Bid-NMe**_**2**_ via **Int-NMe**_**2**_. Optimizations performed at the ωB97XD(toluene)/def2TZVP level of theory.

The effect of the R group on the formation of the bidentate complexes **5-R** was further studied by DFT calculations (see SI for details). The structures of monodentate complexes **4-R** (R = NMe_2_, OMe, Me, H, F, CF_3_) were optimized as well as the analogous bidentate complexes [Ru(*p*-cymene)(κ^2^*P,N*-(**1-R**))Cl][Cl] labeled as **Bid-R** (note these bidentate complexes studied computationally contain the Cl^–^ counteranion instead of those accessed experimentally, **5-R**, which feature the BPh_4_^–^ counteranion, Figure 7A). In all cases, the monodentate complex **4-R** is favored over the analogous bidentate complex **Bid-R** (see SI for all values); this is in line with the experimental data in which the bidentate complexes do not form spontaneously from the corresponding monodentate complex and a halide-abstracting agent is required to promote the reaction. However, the bidentate complexes become less disfavored when electron-donating substituents are used (Figure 7B), which is rationalized by the donor sites on the azophosphine becoming more electron rich. These data highlight the importance of electron-donating para substituents when forming bidentate complexes with azophosphines. Despite the formation of the bidentate complexes being thermodynamically uphill relative to the monodentate complexes, this reaction is driven by precipitation of the corresponding NaCl salt following halide abstraction and subsequent formation of an intermediate (**Int-R**; Figure 7). For all of the R groups explored, **Bid-R** is more energetically favored than the corresponding **Int-R**; for R = NMe_2_, this value is 6.26 kcal·mol^–1^, whereas for the electron-withdrawing R = CF_3_, this value is only 3.38 kcal·mol^–1^ (see SI for all values; the reaction profile for the NMe_2_-substituted compounds is shown in Figure 7C).

To extend this computational insight, in our previous publication we explored the complexation of the *N*-mesityl-substituted azophosphine, MesN_2_P^*t*^Bu_2_, and showed that although it was simple to access the monodentate Ru complex Ru(*p*-cymene)(κ^1^*P*-(MesN_2_P^*t*^Bu_2_))Cl_2_ (cf. **4-R**), despite extensive synthetic efforts we could not synthesize the bidentate analogue (cf. **5-R**). We therefore carried out the same computational study on this particular azophosphine and found that the final bidentate product is actually higher in energy than the halide-abstracted intermediate by 6.49 kcal·mol^–1^, so although halide abstraction is experimentally possible, the formation of the bidentate complex is disfavored. This result shows that the steric profile of the *N*-aryl group on the azophosphine is key, and in the mesityl case, the *ortho*-methyl substituents are bulky enough to preclude formation of the bidentate complex.

### Catalysis

**Figure 8:**
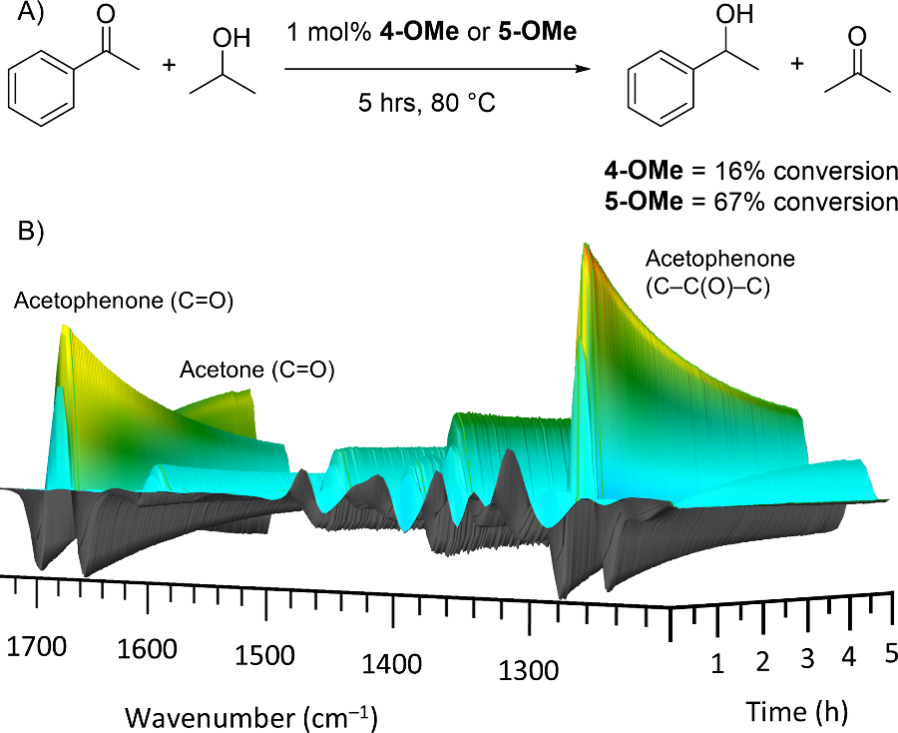
(A) Base-free transfer hydrogenation of acetophenone with catalysts **4-OMe** and **5-OMe**. (B) FTIR surface plot showing changes in IR spectra over time for reaction using **5-OMe** as catalyst.

Finally, we explored the monodentate and bidentate complexes as catalysts in the transfer hydrogenation of acetophenone to 1-phenylethanol. We hypothesized that the azophosphine could act as a hybrid ligand to enable this process to occur in a base-free manner.^1^ To this end, the base-free transfer hydrogenation of acetophenone using isopropanol as a hydrogen source was carried out using 1 mol % of **4-OMe** or **5-OMe** as catalyst (Figure 8A) and the reactions followed by FTIR spectroscopy using ReactIR. ReactIR allows for in situ monitoring of the reactant and/or product without the need for offline analysis.^35^ The use of standard addition (see SI) enabled quantification of acetophenone concentration and calculation of its conversion by following the isolated, characteristic peak at 1267 cm^–1^ (C–C(O)–C stretch). As the peaks in the FTIR spectra assigned to 1-phenylethanol were not sufficiently resolved, reliable quantification of product yield was not possible by this method. ^1^H NMR spectroscopy instead provided an integration ratio of acetophenone:1-phenylethanol in the reaction mixture to provide their relative percent composition. Note this value does not equate to a percent yield of 1-phenylethanol as it assumes selective conversion of acetophenone, although no other side products were observed in the ^1^H NMR spectra.

When using the monodentate **4-OMe** as the catalyst, the reaction was sluggish, although it is noteworthy that some of the target 1-phenylethanol was observed despite the lack of external base in this reaction. The FTIR reaction monitoring for this reaction reproducibly showed erratic spikes in the spectra at various time points, which precludes any detailed discussion of kinetics, but after 5 h, approximately 16% of the acetophenone had been converted. An aliquot of the reaction mixture taken after 16 h was analyzed by ^1^H NMR spectroscopy and showed a 79:21 ratio of acetophenone:1-phenylethanol. The experiment using the bidentate **5-OMe** as the catalyst was far more promising. The surface plot of **5-OMe** showed the consumption of acetophenone, represented by the decrease in the peak at 1681 cm^–1^ (C=O stretch, acetophenone) and the tracked peak at 1267 cm^–1^ with time (Figure 8B). The production of acetone as a byproduct immediately after addition of **5-OMe** was observed, represented by the characteristic peak at 1710 cm^–1^ (C=O stretch). After 5 h, the conversion of acetophenone was 67%. ^1^H NMR spectroscopic analysis after 16 h showed a relative composition of 11% acetophenone to 89% 1-phenylethanol. Further studies showed that benzaldehyde can be reduced to benzyl alcohol faster than the analogous reaction for acetophenone (see SI for details). However, formic acid is not a suitable hydrogen source in place of isopropanol for this transfer hydrogenation chemistry, and 0% conversion for the reduction of acetophenone was observed in this case. These results provide a proof-of-concept that the azophosphine ligand is capable of promoting the base-free transfer hydrogenation of acetophenone and benzaldehyde and that the bidentate complex **5-OMe** is a more active catalyst than the monodentate **4-OMe**.

## Conclusions

In this study, we have broadened the family of azophosphines **1-R** by systematically varying the electronic nature of the *N*-aryl substituent. The changes in electronics yield clear trends in the NMR and UV–vis spectroscopic measurements of these compounds and also have a minor effect on the structure of the azophosphines as explored by single-crystal X-ray diffraction and DFT studies. The azophosphine-selenides **3-R** were synthesized and the ^1^*J*_P–Se_ coupling constants measured to assess the donor strength of the phosphine center; these results showed that azophosphines in general are relatively weaker phosphine donors but that the strength can be fine tuned with the more electron-donating *N*-aryl substituents giving stronger azophosphine donors. This finding was also borne out in the coordination chemistry studies, where the stronger donors afforded the bidentate [Ru(*p*-cymene)(κ^2^*P,N*-(**1-R**))Cl][BPh_4_] (**5-R**) complexes more cleanly. Finally, we explored the Ru complexes **4-OMe** and **5-OMe** as catalysts in the transfer hydrogenation of acetophenone. Both catalysts could promote this transformation without the need for harsh external bases, and the bidentate complex **5-OMe** was a much more efficient catalyst. These results show that azophosphines are valuable hybrid ligands that can be readily tuned and can promote important catalytic reactions. Work to explore the role of azophosphines and related ligands in catalysis is ongoing in our laboratory.

## Experimental Section

### General Remarks

Except as otherwise noted, the syntheses of **1-R**, **2-R** (R = OMe, Me, H, F, CF_3_), **3-R** (R = NMe_2_, OMe, Me, H, F, CF_3_), **4-OMe**, and **5-OMe** were performed using standard Schlenk line technique under a flow of dry, oxygen-free nitrogen or an MBraun ECO glovebox under an atmosphere of dry, oxygen-free nitrogen with water and oxygen levels maintained at < 0.1 ppm. Room temperature (RT) refers to reactions where no thermostatic control was applied and the temperature was recorded as 16–25 °C. Unless otherwise stated, overnight reactions refer to a period of 16 h, and degassing refers to three freeze–pump–thaw cycles. All NMR spectra were collected on a Bruker 500 MHz AV_NEO Advance NMR Spectrometer or a Bruker 400 MHz AV_NEO Advance NMR Spectrometer. Chemical shifts are reported in ppm; coupling constants (*J*) are reported in Hertz (Hz). ^1^H and ^13^C NMR spectra were referenced internally to the most upfield solvent peak; ^31^P and ^31^P{^1^H} NMR spectra are externally referenced to 85% H_3_PO_4_; ^11^B{^1^H} NMR spectra are externally referenced to BF_3_·OEt_2_; ^19^F{^1^H} NMR spectra are externally referenced to CFCl_3_. All UV–vis absorption spectra were collected on a Cary50 UV–vis spectrometer. The samples were prepared as 5 × 10^–5^ M solutions in toluene or DCM to a volume of 3 mL and irradiated in a quartz glass High Precision Cell cuvette (10 × 10 mm) from Hellma Analytics. Air-sensitive UV–vis samples were collected using a sealable cuvette with solutions made up in the glovebox. All IR spectra were collected on a Perkin-Elmer Spectrum Two FT-IR spectrometer using the attenuated total reflection (ATR) sampling technique. In situ ReactIR measurements were taken with a Mettler Toledo ReactIR 700 and a 6 × 1.5 mm AgX Fiber DiComp probe. The FTIR data underwent second-derivative processing using the standard function in the Mettler-Toledo iCIR software. The spectra were recorded every minute, and each was comprised of 159 scans. The acetophenone was monitored using the height of the signal at 1267 cm^–1^ relative to a two-point baseline (1284 and 1252 cm^–1^). All mass spectra were collected on a Waters Xevo G2-XS TOF mass spectrometer using electrospray ionization (ESI) positive ion mode or atmospheric solids analysis probe (ASAP) with atmospheric pressure ionization positive ion mode. Using ESI, samples were dissolved in dry acetonitrile and directly injected into the ESI ionization chamber via a 250 μL glass syringe. Using ASAP, samples were run neat by dipping a glass capillary into the sample vial before insertion into the ASAP source. For HRMS, the three major monoisotopic masses are provided for each compound. Single-crystal X-ray diffraction data were collected at either 100 or 120 K on an Agilent SuperNova A diffractometer using an Atlas detector using Cu Kα radiation (λ = 1.54184). Details of structure solution and refinement can be found in the Supporting Information. Compounds **1-NMe**_**2**_ and **2-NMe**_**2**_ were first reported in our earlier communication,^7^ in which the synthetic details and characterization data can be found.

### General Procedure for the Synthesis of Arenediazonium Tetrafluoroborates

***Caution*****: diazonium salts can undergo violent decomposition and explosion upon isolation, often governed by the counterion. The tetrafluoroborate salts are often significantly more stable; however, caution in their preparation is still essential.**(36) Arenediazonium tetrafluoroborates were prepared following a standard literature procedure.^37,38^ To the substituted aniline (2.0 mmol, 1 equiv) in water (1 mL), 50 wt % aq. HBF_4_ (0.68 mL) was added. The mixture was cooled in an ice–water bath, and a solution of NaNO_2_ (2.0 mmol, 1 equiv) in water (0.4 mL) was added dropwise. The mixture was stirred for 45 min at 0 °C after which the precipitate was collected by Buchner filtration, washed with diethyl ether (3 × 20 mL), and dried in vacuo to obtain the crude arenediazonium tetrafluoroborate [ArN_2_][BF_4_]. The crude arenediazonium tetrafluoroborate was dissolved in minimal acetonitrile, filtered if necessary, and recrystallized by addition to excess diethyl ether (approximately 70 mL) with addition of further diethyl ether until no more precipitate formed. The precipitate was collected by Buchner filtration, washed with diethyl ether (3 × 20 mL), and dried in vacuo to obtain the purified arenediazonium tetrafluoroborate [ArN_2_][BF_4_]. The salts were transferred to foil-wrapped vials and stored in the freezer at −35 °C.

#### *p*-Dimethylaminobenzenediazonium Tetrafluoroborate [*p*-(NMe_2_)C_6_H_4_N_2_][BF_4_]

Isolated as a dark gray solid (235.0 mg, 53%). ^1^H (400.1 MHz, CD_3_CN, 298 K): δ 8.00 (d, ^3^*J*_H–H_ = 10 Hz, 2H*; H*_Ar_), 6.93 (d, ^3^*J*_H–H_ = 10 Hz, 2H; *H*_Ar_), 3.26 (s, 6H; N(C*H*_3_)_2_. ^19^F{^1^H} (376.5 MHz, CD_3_CN, 298 K): δ −152.0 (s; ^11^B*F*_4_), −151.9 (s; ^10^B*F*_4_).

#### *p*-Anisyldiazonium Tetrafluoroborate [*p*-(OMe)C_6_H_4_N_2_][BF_4_]

Isolated as a white solid (1.1 g, 65%). ^1^H (400.1 MHz, CD_3_CN, 298 K): δ 8.43–8.39 (m, 2H; H_Ar_), 7.36–7.32 (m, 2H; H_Ar_), 4.06 (s; 3*H*, *p*-OC*H*_3_). ^19^F{^1^H} (376.5 MHz, CD_3_CN, 298 K): δ −151.6 (s; ^11^B*F*_4_), −151.6 (s; ^10^B*F*_4_).

#### *p*-Tolyldiazonium Tetrafluoroborate [*p*-(Me)C_6_H_4_N_2_][BF_4_]

Isolated as a white solid (157.2 mg, 38%). ^1^H (400.1 MHz, CD_3_CN, 298 K): δ 8.36 (d, ^3^*J*_H–H_ = 9 Hz, 2H; H_Ar_), 7.73 (d, ^3^*J*_H–H_ = 9 Hz, 2H; H_Ar_), 2.61 (s; 3H, *p*-C*H*_3_). ^19^F{^1^H} (376.5 MHz, CD_3_CN, 298 K): δ −151.6 (s; ^11^B*F*_4_), −151.7 (s; ^10^B*F*_4_).

#### Benzenediazonium Tetrafluoroborate [C_6_H_4_N_2_][BF_4_]

Isolated as an off-white solid (121.8 mg, 31%). ^1^H (400.1 MHz, CD_3_CN, 298 K): δ 7.90–7.94 (m, 2H; *H*_Ar_), 8.25 (tt, ^3^*J*_H–H_ = 8 Hz, ^4^*J*_H–H_ = 1 Hz, 1H; *H*_Ar_), 8.49–8.51 (m, 2H; *H*_Ar_). ^19^F{^1^H} (376.5 MHz, CD_3_CN, 298 K): δ −150.3 (s; B*F*_4_).

#### *p*-Fluorobenzenediazonium Tetrafluoroborate [*p*-(F)C_6_H_4_N_2_][BF_4_]

Isolated as a white solid (368.2 mg, 43%). ^1^H (400.1 MHz, CD_3_CN, 298 K): δ 8.61–8.58 (m, 2H; *H*_Ar_), 7.69–7.64 (m, 2H; *H*_Ar_). ^19^F{^1^H} (376.5 MHz, CD_3_CN, 298 K): δ −84.76 (s; p-*F*), −151.4 (s; ^11^B*F*_4_), −151.5 (s; ^10^B*F*_4_).

#### *p*-(Trifluoromethyl)benzenediazonium Tetrafluoroborate [*p*-(CF_3_)C_6_H_4_N_2_][BF_4_]

Isolated as a pale yellow solid (860 mg, 45%). ^1^H (400.1 MHz, CD_3_CN, 298 K): δ 8.74–8.68 (m, 2H; *H*_Ar_), 8.34–8.16 (m, 2H; *H*_Ar_). ^19^F{^1^H} (376.5 MHz, CD_3_CN, 298 K): δ −64.8 (s; *p*-C*F*_3_), −151.1 (s; B*F*_4_).

### General Procedure for Synthesis of Azophosphine-Borane Adducts **2-R**

A 25 mL Schlenk flask was charged with a 0.25 M THF solution of H^*t*^Bu_2_P·BH_3_ (1 equiv) and cooled to −78 °C. A 1.42 M cyclohexane solution of *sec*-butyllithium (1 equiv) was added dropwise, resulting in a color change from colorless to pale yellow. The solution was allowed to warm to room temperature over 2 h and then stirred for a further 30 min at room temperature. This solution was added dropwise to a separate Schlenk containing a slurry of arenediazonium tetrafluoroborate salt (1 equiv) in THF at −78 °C, resulting in an immediate color change. The reaction was left to warm slowly to room temperature and stirred for 1 h. The volatiles were removed in vacuo, and the product was isolated by column chromatography. Removal of the volatiles in vacuo yielded the product as an intensely colored solid. The air-stable, solid products were stored in vials, away from direct light, in the freezer.

#### *p*-(OMe)C_6_H_4_N_2_P^*t*^Bu_2_·BH_3_ (**2-OMe**)

Borane azophosphine **2-OMe** was synthesized from precursor [*p*-(OMe)C_6_H_4_N_2_][BF_4_] (5.1 mmol, 1.1 mg, 1 equiv). Purification by column chromatography (eluent = 0 → 10% diethyl ether in hexane) obtained **2-OMe** as a pink powder (1.0 g, 66%). Single crystals of **2-OMe** suitable for single-crystal X-ray diffraction were grown by slow evaporation of a toluene solution of the product at room temperature. ^1^H (500.1 MHz, CDCl_3,_ 298 K): δ 7.86 (d, ^3^*J*_H–H_ = 9 Hz, 2H; *H*_Ar_), 6.99 (d, ^3^*J*_H–H_ = 9 Hz, 2H; *H*_Ar_), 3.90 (s, 3H; *p*-OC*H*_3_), 1.39 (d, ^3^*J*_H–P_ = 12 Hz, 18H; ^*t*^Bu), 0.56 (br quart., 3H; B*H*_3_). ^13^C{^1^H} (125.8 MHz, CDCl_3_): δ 163.8 (s, *p*-*C*_Ar_), 149.9 (d; ^3^*J*_C–P_ = 37 Hz; *i*-*C*_Ar_), 124.9 (s; *o*-*C*_Ar_), 114.3 (s; *m*-*C*_Ar_), 55.9 (s, *p-*O*C*H_3_), 35.1 (d; ^1^*J*_C–P_ = 25 Hz*; C*(CH_3_)_3_), 27.7 (s; *C*(CH_3_)_3_). ^31^P{^1^H} (202.4 MHz, CDCl_3_): δ 103.9 (br quart.). ^31^P (162.0 MHz, CDCl_3_): δ 103.9 (br s). ^11^B{^1^H} (128.4 MHz, CDCl_3_): δ −42.5 (d, ^1^*J*_B–P_ = 51 Hz). UV–vis (toluene, nm) λ_max_: 334 (s), 499 (w). ESI-HRMS *m*/*z* for C_15_H_27_N_2_OPB [M – H]^+^: calcd (found) 292.1990 (292.1996), 293.1957 (293.1964), 294.1987 (294.2044). IR (cm^–1^) ν_max_: 2988 (w), 2967 (w), 2868 (w), 2407 (m, B–H), 1593 (s, N=N).

#### *p*-(Me)C_6_H_4_N_2_P^*t*^Bu_2_·BH_3_ (**2-Me**)

Borane azophosphine **2-Me** was synthesized from precursor [*p*-(Me)C_6_H_4_N_2_][BF_4_] (1.6 mmol, 328.0 mg, 1 equiv). Purification by column chromatography (eluent = 0 → 5% diethyl ether in hexane) obtained **2-Me** as a pink powder (305.0 mg, 69%). Single crystals of **2-Me** suitable for single-crystal X-ray diffraction were grown by slow evaporation of a toluene solution of the product at −35 °C. ^1^H (400.1 MHz, CD_3_CN_,_ 298 K): δ 7.73 (d, ^3^*J*_H–H_ = 8 Hz, 2H; *H*_Ar_), 7.39 (d, ^3^*J*_H–H_ = 8 Hz, 2H; *H*_Ar_), 2.43 (s, 3H; *p*-C*H*_3_, 1.36 (d, ^3^*J*_H–P_ = 12 Hz, 18H; ^*t*^Bu), 0.47 (br quart., 3H; B*H*_3_). ^13^C{^1^H} (125.8 MHz, CD_3_CN): δ 154.1 (d; ^3^*J*_C–P_ = 36 Hz; *i*-*C*_Ar_), 145.5 (s, *p*-*C*_Ar_), 131.0 (s; *C*_Ar_), 123.2 (s; *C*_Ar_), 35.7 (d; ^1^*J*_C–P_ = 25 Hz; *C*(CH_3_)_3_), 27.8 (s; *C*(CH_3_)_3_), 21.6 (s; *p-*CH_3_). ^31^P{^1^H} (202.4 MHz, CD_3_CN): δ 106.2 (br quart.). ^31^P (162.0 MHz, CD_3_CN): δ 106.2 (br s). ^11^B{^1^H} (128.4 MHz, CD_3_CN): δ −43.0 (d, ^1^*J*_B–P_ = 52 Hz). UV–vis (toluene, nm) λ_max_: 310 (s), 510 (w). ESI-HRMS *m*/*z* for C_15_H_27_BN_2_P [M – H]^+^: calcd (found) 276.2041 (276.2039), 277.2008 (277.2008), 278.2038 (278.2047), 279.2068 (279.2146). IR (cm^–1^) ν_max_: 3727 (w), 3700 (w), 3627 (w), 3599 (w), 2983 (w), 2965 (m), 2921 (w), 2868 (w), 2414 (m), 2393 (m), 2333 (m, B–H), 1739 (w), 1598 (w, N=N), 1501 (m).

#### C_6_H_4_N_2_P^*t*^Bu_2_·BH_3_ (**2-H**)

Borane azophosphine **2-H** was synthesized from precursor [C_6_H_4_N_2_][BF_4_] (1.9 mmol, 360 mg, 1 equiv). Purification by column chromatography (eluent = 0 → 5% diethyl ether in hexane) obtained **2-H** as a pink/red powder (296 mg, 60%). Single crystals of **2-H** suitable for single-crystal X-ray diffraction were grown by slow evaporation of a toluene solution of the product at room temperature. ^1^H (400.1 MHz, CD_3_CN_,_ 298 K): δ 7.84–7.81 (m, 2H; *H*_Ar_), 7.66–7.57 (m, 3H; *H*_Ar_), 1.38 (d, ^3^*J*_H–P_ = 13 Hz, 18H; ^*t*^Bu), 0.48 (br quart., 3H; B*H*_3_). ^13^C{^1^H} (101 MHz, CD_3_CN, 289 K): δ 155.6 (d, ^3^*J*_C–P_ = 35 Hz; *i*-*C*_Ar_), 134.2 (s, *C*_Ar_), 130.5 (s, *C*_Ar_), 123.1 (s, *C*_Ar_), 35.7 (d, ^1^*J*_C–P_ = 25 Hz; *C*(CH_3_)_3_), 27.8 (s, C(*C*H_3_)_3_). ^31^P{^1^H} (162 MHz, CDCN, 298 K): δ 107.6 (br quart.). ^31^P (162.0 MHz, CD_3_CN, 298 K): δ 107.6 (br s). ^11^B{^1^H} (128.4 MHz, CD_3_CN, 298 K): δ −42.9 (d, ^1^*J*_B-P_ = 51 Hz). UV–vis (toluene, nm) λ_max_: 296 (s), 509 (w). ESI-HRMS *m*/*z* for C_14_H_25_BN_2_P [M – H]^+^: calcd (found) 262.1885 (262.1882), 263.1851 (263.1855), 264.1883 (264.1883). IR (cm^–1^) ν_max_: 2969 (w), 2952 (w), 2921.2 (w), 2871 (w), 2408 (m), 2372 (m, B–H), 1738 (m), 1477 (m, N=N).

#### *p*-(F)C_6_H_4_N_2_P^*t*^Bu_2_·BH_3_ (**2-F**)

Borane azophosphine **2-F** was synthesized from precursor [*p-*(F)C_6_H_4_N_2_][BF_4_] (1.9 mmol, 393 mg, 1 equiv). Purification by column chromatography (eluent = 0 → 5% diethyl ether in hexane) obtained **2-F** as a red solid (318 mg, 60%). Single crystals of **2-F** suitable for single-crystal X-ray diffraction were grown by slow evaporation of a hexane solution of the product at room temperature. ^1^H NMR (400 MHz, CD_3_CN, 298 K): δ 7.94–7.84 (m, 2H; *H*_Ar_), 7.37–7.27 (m, 2H; *H*_Ar_), 1.37 (d, ^3^*J*_H–P_ = 12.7 Hz, 18H; ^*t*^Bu), 0.47 (br quart., 3H; B*H*_3_). ^13^C{^1^H} (101 MHz, CD_3_CN, 289 K): δ 166.6 (d, ^1^*J*_C–F_ = 253 Hz; *p-C*F), 152.5 (dd, ^3^*J*_C–P_ = 36 Hz, ^4^*J*_C–F_ = 3 Hz; *i*-*C*_Ar_), 125.68 (dd, ^3^*J*_C–F_ = 10, ^4^*J*_C–P_ = 2 Hz, *o*-*C*_Ar_), 117.4 (d, ^2^*J*_C–F_ = 23 Hz, *m*-*C*_AR_), 35.7 (d, ^1^*J*_C–P_ = 25 Hz; *C*(CH_3_)_3_), 27.8 (s, C(*C*H_3_)_3_). ^31^P{^1^H} (162 MHz, CDCN, 298 K): δ 107.9 (br quart.). ^31^P (162.0 MHz, CDCN, 298 K): δ 107.9 (br s). ^19^F{^1^H} (377 MHz, CD_3_CN, 298 K): δ −108.0 (s). ^11^B{^1^H} (128.4 MHz, CD_3_CN, 298 K): δ −43.0 (d, ^1^*J*_B-P_ = 51 Hz). UV–vis (toluene, nm) λ_max_: 301 (s), 510 (w). ESI-HRMS *m*/*z* for C_14_H_24_BN_2_FP [M – H]^+^: calcd (found) 280.1790 (280.1787), 281.1757 (281.1754), 282.1786 (282.1791), 283.1817 (283.1872). IR (cm^–1^) ν_max_: 2983 (w), 2966 (w) 2870 (w), 2382 (m, B–H), 1589 (m, N=N), 1497 (s).

#### *p*-(CF_3_)C_6_H_4_N_2_P^*t*^Bu_2_·BH_3_ (**2-CF_3_**)

Borane azophosphine **2-CF**_**3**_ was synthesized from precursor [*p-(*CF_3_)C_6_H_4_N_2_][BF_4_] (1.3 mmol, 350 mg, 1 equiv). Purification by column chromatography (eluent = 0 → 5% diethyl ether in hexane) followed by further purification by column chromatography (eluent = 0 → 2% diethyl ether in hexane) obtained **2-CF**_**3**_ as a purple solid (162 mg, 46%). Single crystals of **2-CF**_**3**_ suitable for single-crystal X-ray diffraction were grown by slow evaporation of a hexane solution of the product at −35 °C. ^1^H NMR (400 MHz, CD_3_CN, 298 K): δ 7.95 (app. d, *J* = 8.6 Hz, 2H; *H*_Ar_), 7.91 (d, *J* = 8.6 Hz, 2H; *H*_Ar_), 1.40 (d, ^3^*J*_H–P_ = 12.7 Hz, 18H; ^*t*^Bu), 0.49 (br quart., 3H; B*H*_3_). ^13^C{^1^H} (101 MHz, CD_3_CN, 289 K): δ 156.7 (d, ^3^*J*_C–P_ = 35 Hz; *i*-*C*_Ar_), 134.2 (q, ^2^*J*_C–F_ = 32 Hz; *p*-*C*CF_3_), 127.8 (q, ^4^*J*_C–F_ = 4 Hz; *m*-*C*_Ar_), 123.58 (q, ^1^*J*_C–F_ = 272 Hz; *C*F_3_); 123.6 (s, *o*-*C*_Ar_), 35.9 (d, ^1^*J*_C–P_ = 24 Hz; *C*(CH_3_)_3_), 27.8 (s, C(*C*H_3_)_3_). ^31^P{^1^H} (162 MHz, CD_3_CN, 298 K): δ 111.4 (br quart.). ^31^P (162.0 MHz, CD_3_CN, 298 K): δ 111.4 (br s). ^19^F{^1^H} (377 MHz, CD_3_CN, 298 K): δ −63.3 (s). ^11^B{^1^H} (128.4 MHz, CD_3_CN, 298 K): δ −42.9 (d, ^1^*J*_B-P_ = 50 Hz). UV–vis (toluene, nm) λ_max_: 281 (s), 523 (w). ESI-HRMS *m*/*z* for C_15_H_24_BN_2_F_3_P [M – H]^+^: calcd (found) 330.1758 (330.1764), 331.1725 (331.1737), 332.1755 (332.1757), 333.1786 (333.1821). IR (cm^–1^) ν_max_: 2970 (w), 2925 (w), 2872 (w), 2361 (m), 2348 (m, B–H), 1738 (w) 1609 (w, N=N).

### General Procedure for Synthesis of Azophosphines **1-R**

RN_2_P^*t*^Bu_2_·BH_3_ (1 equiv) was added to a 25 mL Schlenk ampule and dissolved in toluene. To this, pyrrolidine was added (10 equiv), and the mixture was stirred at room temperature overnight (16 h), which gave 100% conversion to RN_2_P^*t*^Bu_2_ by crude ^31^P{^1^H} NMR spectroscopy. Excess pyrrolidine was removed in vacuo, and the resulting oil was filtered through a silica plug (eluent = 10% diethyl ether in hexane) to remove the pyrrolidine·BH_3_ adduct. Removal of the eluent in vacuo obtained the target deprotected RN_2_P^*t*^Bu_2_ product as a dark-colored solid or oil. The air-sensitive products were stored in vials in the glovebox freezer.

#### *p*-(OMe)C_6_H_4_N_2_P^*t*^Bu_2_ (**1-OMe**)

Azophosphine **1-OMe** was synthesized from azophosphine borane 2-OMe (1.2 mmol, 343 mg, 1 equiv). The reaction was stirred at room temperature overnight (16 h) to obtain 100% conversion of **2-OMe** to **1-OMe** and isolated as a dark red solid (248 mg, 76%). The flask was then rinsed with toluene-*d*_8_ to prepare an NMR sample. ^1^H (500.1 MHz, tol-*d*_8,_ 298 K): δ 7.68 (m, 2H; *m*-*H*_Ar_), 6.67 (m, 2H; *o*-*H*_Ar_), 3.25 (s, 3H; *p*-OC*H*_3_), 1.32 (d, ^3^*J*_H–P_ = 11 Hz, 18H; ^*t*^Bu). ^13^C{^1^H} (125.8 MHz, tol-*d*_8_): δ 161.9 (s; *p*-*C*_Ar_), 150.2 (d, ^3^*J*_C–P_ = 22 Hz; *i*-*C*_Ar_), 122.3 (s; *m*-*C*_Ar_), 114.2 (s; *o*-*C*_Ar_), 54.9 (s; *p*-O*C*H_3_), 36.3 (d, ^1^*J*_C–P_ = 26 Hz; *C*(CH_3_)_3_), 29.2 (d, ^2^*J*_C–P_ = 12 Hz; C(*C*H_3_)_3_). ^31^P{^1^H} (202.4 MHz, tol-*d*_8_): δ 111.6 (s). ^31^P (202.4 MHz, tol-*d*_8_): δ 111.6 (br m). UV–vis (toluene, nm) λ_max_: 354 (s), 504 (w). ASAP-HRMS *m*/*z* for C_15_H_26_N_2_OP [M – H]^+^: calcd (found) 282.1814, 281.1783; found 282.1824, 281.1789. IR (cm^–1^) ν_max_: 3070 (w), 2974 (m), 2940 (m), 2891 (m), 2860 (m), 1601 (s, N=N), 1579 (s), 1502 (s).

#### *p*-(CH_3_)C_6_H_4_N_2_P^*t*^Bu_2_ (**1-Me**)

Azophosphine was synthesized from azophosphine borane **2-Me** (0.4 mmol, 100 mg, 1 equiv). The reaction was stirred at room temperature overnight (16 h) to obtain 100% conversion of **2-Me** to **1-Me** and isolated as a dark red oil (70 mg, 72%). The flask was then rinsed with toluene-*d*_8_ to prepare an NMR sample. ^1^H (400.1 MHz, tol-*d*_8,_ 298 K): δ 7.62 (d, ^3^*J*_H–H_ = 8 Hz, 2H; *m*-*H*_Ar_), 6.94 (d, ^3^*J*_H–H_ = 8 Hz, 2H; *o*-*H*_Ar_), 2.05 (s, 3H; *p*-C*H*_3_), 1.31 (d, ^3^*J*_H–P_ = 11 Hz, 18H; ^*t*^Bu). ^13^C{^1^H} (100.6 MHz, tol-*d*_8_): δ 153.6 (d, ^3^*J*_C–P_ = 22 Hz; *i*-*C*_Ar_), 140.4 (s; *p*-*C*_Ar_), 129.8 (s; *m*-*C*_Ar_), 121.6 (s; *o*-*C*_Ar_), 36.43 (d, ^1^*J*_C–P_ = 26; *C*(CH_3_)_3_), 29.2 (d, ^2^*J*_C–P_ = 12 Hz; C(*C*H_3_)_3_), 21.1 (s; *p*-*C*H_3_). ^31^P{^1^H} (202.4 MHz, tol-*d*_8_): δ 113.4 (s). ^31^P (202.4 MHz, tol-*d*_8_): δ 113.4 (br m). UV–vis (toluene, nm) λ_max_: 353 (s), 504 (w). ASAP-HRMS *m*/*z* for C_15_H_26_N_2_P [M – H]^+^: calcd 265.1833 (found 265.1833), 266.1865 (266.1864). IR (cm^–1^) ν_max_: 2979 (w), 2940 (w), 2893 (w), 2862 (w), 1602 (w, N=N).

#### C_6_H_4_N_2_P^*t*^Bu_2_ (**1-H**)

Azophosphine **1-H** was synthesized from azophosphine borane **2-H** (0.4 mmol, 100 mg, 1 equiv). The reaction was stirred at room temperature overnight (16 h) to obtain 100% conversion of **2-H** to **1-H** and isolated as a dark red oil (53 mg, 56%). The flask was then rinsed with toluene-*d*_8_ to prepare an NMR sample. ^1^H (500.1 MHz, tol-*d*_8,_ 298 K): δ 7.66–7.64 (m, 2H; *H*_Ar_), 7.15–7.11 (m, 2H; *H*_Ar_), 7.06–7.03 (m, 1H; *p*-*H*_Ar_), 1.30 (d, ^3^*J*_H–P_ = 11 Hz, 18H; ^*t*^Bu). ^13^C{^1^H} (125.7 MHz, tol-*d*_8_): δ 155.0 (d, ^3^*J*_C–P_ = 21 Hz; *i*-*C*_Ar_), 130.3 (s; *p*-*C*_Ar_), 129.1 (s; *m*-*C*_Ar_), 121.6 (s; *o*-*C*_Ar_), 36.5 (d, ^1^*J*_C–P_ = 26 Hz; *C*(CH_3_)_3_), 29.2 (d, ^2^*J*_C–P_ = 12 Hz; C(*C*H_3_)_3_). ^31^P{^1^H} (202.4 MHz, tol-*d*_8_): δ 115.1 (s). ^31^P (202.4 MHz, tol-*d*_8_): δ 115.1 (br m). UV–vis (toluene, nm) λ_max_: 350 (s), 512 (w). ASAP-HRMS *m*/*z* for C_14_H_24_N_2_P [M – H]^+^: calcd (found) 251.1677 (251.1682), 252.1708 (252.1714), 253.1739 (253.1763). IR (cm^–1^) ν_max_: 2978 (w), 2941 (w), 2894 (w), 2861 (w), 1593 (w, N=N).

#### *p*-(F)C_6_H_4_N_2_P^*t*^Bu_2_ (**1-F**)

Azophosphine **1-F** was synthesized from azophosphine borane **2-F** (0.4 mmol, 100 mg, 1 equiv). The reaction was stirred at room temperature overnight (16 h) to obtain 100% conversion of **2-F** to **1-F**, and isolated as a dark red oil (63 mg, 63%). The flask was then rinsed with toluene-*d*_8_ to prepare an NMR sample. ^1^H (500.1 MHz, tol-*d*_8,_ 298 K): δ 7.48–7.44 (m, 2H; *H*_Ar_), 6.75–71 (m, 2H; *H*_Ar_), 1.28 (d, ^3^*J*_H–P_ = 11 Hz, 18H; ^*t*^Bu). ^13^C{^1^H} (125.7 MHz, tol-*d*_8_): δ 164.3 (dd; *p*-*C*_Ar_), 151.6 (dd; *i*-*C*_Ar_), 123.3 (d, ^3^*J*_C–P_ = 9 Hz; *m*-*C*_Ar_), 115.9 (d, ^2^*J*_C–F_ = 26 Hz; *o*-*C*_Ar_), 36.5 (d, ^1^*J*_C–P_ = 26 Hz; *C*(CH_3_)_3_), 29.2 (d, ^2^*J*_C–P_ = 12 Hz; C(*C*H_3_)_3_). ^31^P{^1^H} (162.0 MHz, tol-*d*_8_): δ 115.2 (s). ^31^P (162.0 MHz, tol-*d*_8_): δ 115.2 (br m). ^19^F{^1^H} (470.5 MHz, tol-*d*_8_): δ −111.0 (br m). UV–vis (toluene, nm) λ_max_: 353 (s), 506 (w). ASAP-HRMS *m*/*z* for C_14_H_23_FN_2_P [M – H]^+^: calcd (found) 269.1583 (269.1586), 270.1614 (270.1631), 271.1645 (271.1696). IR (cm^–1^) ν_max_: 3055 (w), 2970 (m), 2944 (m), 2897 (w), 2862 (m), 1591 (m, N=N).

#### *p*-(CF_3_)C_6_H_4_N_2_P^*t*^Bu_2_ (**1-CF_3_**)

Azophosphine **1-CF**_**3**_ was synthesized from azophosphine borane **2-CF_3_** (0.3 mmol, 92 mg, 1 equiv). The reaction was stirred at room temperature overnight (16 h) to obtain 100% conversion of **2-CF**_**3**_ to **1-CF**_**3**_ and isolated as a dark red oil (44 mg, 50%). The flask was then rinsed with toluene-*d*_8_ to prepare an NMR sample. ^1^H (500.1 MHz, tol-*d*_8,_ 298 K): δ 7.41–7.39 (m, 2H; *H*_Ar_), 7.31–7.29 (m, 2H; *H*_Ar_), 1.29 (d, ^3^*J*_H–P_ = 11 Hz, 18H; ^*t*^Bu). ^13^C{^1^H} (125.7 MHz, tol-*d*_8_): δ 155.7 (dd; *p*-*C*_Ar_), 131.8–131.0 (m; *p*-*C*_Ar_), 126.5–126.4 (m; *m*-*C*_Ar_), 121.5 (s, *o*-*C*_Ar_), 37.0 (d, ^1^*J*_C–P_ = 27 Hz; *C*(CH_3_)_3_), 29.2 (d, ^2^*J*_C–P_ = 12 Hz; C(*C*H_3_)_3_). ^31^P{^1^H} (202.4 MHz, tol-*d*_8_): δ 119.7 (s). ^31^P (202.4 MHz, tol-*d*_8_): δ 119.7 (br m). ^19^F{^1^H} (470.5 MHz, tol-*d*_8_): δ −62.3 (s). UV–vis (toluene, nm) λ_max_: 361 (s), 519 (w). ASAP-HRMS *m*/*z* for C_15_H_23_F_3_N_2_P [M – H]^+^: calcd (found) 319.1551 (319.1559), 320.1582 (320.1584). IR (cm^–1^) ν_max_: 2980 (w), 2944 (w), 2897 (w), 2864 (w) 1611 (w, N=N).

### General Procedure for Synthesis of Azophosphine-Selenides **3-R**

RN_2_P^*t*^Bu_2_ (1 equiv) was added to a vial and dissolved in toluene-*d*_8_ (1 mL). To this, gray selenium was added (10 equiv), and the mixture was left to stir at room temperature for 30 min. After this time, an aliquot was transferred to a J. Young’s NMR tube, and conversion from RN_2_P^*t*^Bu_2_ to RN_2_P(Se)^*t*^Bu_2_ was monitored by crude ^31^P{^1^H} NMR spectroscopy.

#### *p*-(NMe_2_)C_6_H_4_N_2_P(Se)^*t*^Bu_2_ (**3-NMe_2_**)

Azophosphine-selenide **3-NMe**_**2**_ was synthesized from azophosphine **1-NMe**_**2**_ (0.02 mmol, 6 mg, 1 equiv) to obtain 100% conversion to **3-NMe**_**2**_. ^31^P{^1^H} (202.4 MHz, tol-*d*_8_): δ 108.8 (d, ^1^*J*_P–Se_ = 794 Hz).

#### *p*-(OMe)C_6_H_4_N_2_P(Se)^*t*^Bu_2_ (**3-OMe**)

Azophosphine-selenide **3-OMe** was synthesized from azophosphine **1-OMe** (0.02 mmol, 6 mg, 1 equiv) to obtain 100% conversion to **3-OMe**. ^31^P{^1^H} (202.4 MHz, tol-*d*_8_): δ 112.1 (d, ^1^*J*_P–Se_ = 801 Hz).

#### *p*-(Me)C_6_H_4_N_2_P(Se)^*t*^Bu_2_ (**3-Me**)

Azophosphine-selenide **3-Me** was synthesized from azophosphine **1-Me** (0.02 mmol, 6 mg, 1 equiv) to obtain 100% conversion to **3-Me**. ^31^P{^1^H} (202.4 MHz, tol-*d*_8_): δ 113.3 (d, ^1^*J*_P–Se_ = 804 Hz).

#### C_6_H_4_N_2_P(Se)^*t*^Bu_2_ (**3-H**)

Azophosphine-selenide **3-H** was synthesized from azophosphine **1-H** (0.02 mmol, 6 mg, 1 equiv) to obtain 100% conversion to **3-H**. ^31^P{^1^H} (202.4 MHz, tol-*d*_8_): δ 114.1 (d, ^1^*J*_P–Se_ = 806 Hz).

#### *p*-(F)C_6_H_4_N_2_P(Se)^*t*^Bu_2_ (**3-F**)

Azophosphine-selenide **3-F** was synthesized from azophosphine **1-F** (0.02 mmol, 6 mg, 1 equiv) to obtain 100% conversion to **3-F**. ^31^P{^1^H} (202.4 MHz, tol-*d*_8_): δ 114.4 (d, ^1^*J*_P–Se_ = 806 Hz).

#### *p*-(CF_3_)C_6_H_4_N_2_P(Se)^*t*^Bu_2_ (**3-CF_3_**)

Azophosphine-selenide **3-CF**_**3**_ was synthesized from azophosphine **1-CF**_**3**_ (0.02 mmol, 6 mg, 1 equiv) to obtain 100% conversion to **3-CF**_**3**_. ^31^P{^1^H} (202.4 MHz, tol-*d*_8_): δ 116.3 (d, ^1^*J*_P–Se_ = 810 Hz).

### Synthesis of Coordination Complex κ^1^-*P* Ru(*p*-cymene)Cl_2_(**1-OMe**) (**4-OMe**)

**1-OMe** (0.14 mmol, 40 mg, 1 equiv) was added to a 25 mL round-bottomed Schlenk flask and dissolved in chlorobenzene. To this solution, [Ru(*p*-cymene)Cl_2_]_2_ (0.07 mmol, 44 mg, 0.5 equiv, 1 Ru to 1 azophosphine) was added, and the mixture was stirred at room temperature for 3 h, giving full κ^1^-*P* coordination of the azophosphine. Dropwise addition to hexane gave a dark brown precipitate. This was collected by filtration, washed with further hexane, and dried in vacuo to exclusively obtain **4-OMe** as a dark brown powder (53 mg, 64%). The complexes were stored in vials in the glovebox freezer. The flask was then rinsed with CD_2_Cl_2_ to prepare an NMR sample. Single crystals of **4-OMe** suitable for single-crystal X-ray diffraction were grown from a saturated chlorobenzene solution of the product at −35 °C. ^1^H (400.1 MHz, CD_2_Cl_2,_ 298 K): δ 7.92 (d, ^3^*J*_H–H_ = 9 Hz, 2H; *H*_Ar_ (*p*-anisyl)), 7.10 (d, ^3^*J*_H–H_ = 9 Hz, 2H; *H*_Ar_ (*p*-anisyl)), 5.41 (d, ^3^*J*_H–H_ = 6 Hz, 2H; *H*_Ar_ (*p*-cymene)), 5.18 (d, ^3^*J*_H–H_ = 6 Hz, 2H; *H*_Ar_ (*p*-cymene)), 3.93 (s, 3H; *p*-OC*H*_3_), 2.86 (sept., ^3^*J*_H–H_ = 7 Hz, 1H; C*H*(CH_3_)_2_ (*p*-cymene)), 2.07 (s, 3H; *p*-C*H*_3_ (*p*-cymene)), 1.50 (d, ^3^*J*_H–P_ = 13 Hz, 18H; ^*t*^Bu), 1.05 (d, ^3^*J*_H–H_ = 7 Hz, 6H; CH(C*H*_3_)_2_ (*p*-cymene)). ^13^C{^1^H} (100.6 MHz, CD_2_Cl_2_): δ 164.0 (s; *p*-*C*_Ar_ (*p*-anisyl)), 148.3 (d, ^3^*J*_C–P_ = 33 Hz; *i*-*C*_Ar_ (*p*-anisyl)), 124.4 (s; *m*-*C*_Ar_ (*p*-anisyl)), 115.0 (s; *o*-*C*_Ar_ (*p*-anisyl)), 105.7 (s; *i*-*C*_Ar_ (*p*-cymene)), 97.7 (s; *p*-*C*_Ar_ (*p*-cymene)), 88.0 (s; *o*-*C*_Ar_ (*p*-cymene)), 87.6 (s; *m*-*C*_Ar_ (*p*-cymene)), 56.2 (s; *p*-O*C*H_3_), 42.1 (d, ^1^*J*_C–P_ = 8 Hz; *C*(CH_3_)_3_), 30.6 (s; C(*C*H_3_)_3_), 29.6 (s; *C*H(CH_3_)_2_), 21.8 (s; C(*C*H_3_)_2_), 17.7 (s; *p*-CH_3_ (*p*-cymene)). ^31^P{^1^H} (161.7 MHz, CD_2_Cl_2_): δ 115.2 (s). ^31^P (161.7 MHz, CD_2_Cl_2_): δ 115.2 (br m).: 340 (s), 400–600 (w). ESI-HRMS *m*/*z* for C_25_H_39_ClN_2_OPRu [M – H]^+^: calcd (found) 556.1551 (556.1551), 555.1526 (555.1527), 554.1563 (554.1568), 553.1533 (553.1539). UV–vis (DCM, nm) λ_max_: 552.1548 (552.1554), 551.1536 (551.1542), 550.1544 (550.1549), 549.1541 (549.1548), 548.1550 (548.1558), 547.1544 (547.1555), 545.1564 (545.1568). IR (cm^–1^) ν_max_: 3035 (w), 2967 (w), 2838 (w), 1597 (m, N=N). Elemental Analysis: calcd (found) C% 51.19 (51.00), H% 6.70 (6.63), N% 4.78 (4.79).

### Synthesis of Coordination Complex κ^2^-*P,N* Ru(*p*-cymene)Cl(**1-OMe**) (**5-OMe**)

**1-OMe** (0.29 mmol, 81 mg, 1 equiv) was added to a 25 mL round-bottomed Schlenk flask and dissolved in chlorobenzene. To this solution, [Ru(*p*-cymene)Cl_2_]_2_ (0.14 mmol, 89 mg, 0.5 equiv, 1 Ru to 1 azophosphine) was added, and the mixture was stirred at room temperature for 3 h. Addition of NaBPh_4_ (0.29 mmol, 99 mg, 1 equiv) followed by filtration left behind a white precipitate (NaCl). Chlorobenzene was removed in vacuo, and the dark brown precipitate was washed with hexane to exclusively obtain the κ^2^-*P,N* complex **5-OMe** as a dark brown powder (180 mg, 71%), which was stored in a vial in the glovebox freezer. The flask was then rinsed with CD_2_Cl_2_ to prepare an NMR sample. Single crystals of **5-OMe** suitable for single-crystal X-ray diffraction were grown from a saturated chlorobenzene solution of the product at −35 °C. ^1^H (400.1 MHz, CD_2_Cl_2,_ 298 K): δ 7.87 (d, ^3^*J*_H–H_ = 9 Hz, 2H; *m*-*H*_Ar_ (*p*-anisyl)), 7.38–7.26 (m, 8H; *H*_Ar_ (BPh_4_)), 7.02 (d, 2H; *o*-C*H*_3_ (*p*-anisyl)), 7.02–7.00 (m, 8H; *H*_Ar_ (BPh_4_)), 6.88 (t, ^3^*J*_H–H_ = 7 Hz, 4H; *p*-*H*_Ar_ (BPh_4_)), 5.97 (d, ^3^*J*_H–H_ = 6 Hz, 1H; *H*_Ar_ (*p*-cymene)), 5.92 (d, ^3^*J*_H–H_ = 6 Hz, 1H; *H*_Ar_ (*p*-cymene)), 5.86 (d, ^3^*J*_H–H_ = 6 Hz, 1H; *H*_Ar_ (*p*-cymene)), 5.36 (d, ^3^*J*_H–H_ = 6 Hz, 1H; *H*_Ar_ (*p*-cymene)), 3.92 (s, 3H; *p*-OC*H*_3_ (*p*-anisyl)), 1.80 (s, 3H; *p*-CH_3_ (*p*-cymene)), 1.58 (d, ^3^*J*_H–P_ = 16 Hz, 9H; ^*t*^Bu), 1.49 (d, ^3^*J*_H–P_ = 15 Hz, 9H; ^*t*^Bu), 1.25 (d, ^3^*J*_H–H_ = 7 Hz, 3H; CH(C*H*_3_)_2_ (*p*-cymene)), 1.15 (d, ^3^*J*_H–H_ = 7 Hz, 3H; CH(C*H*_3_)_2_ (*p*-cymene)). ^13^C{^1^H} (125.8 MHz, CD_2_Cl_2_): δ 167.0 (s; *p*-*C*_Ar_ (*p*-anisyl)), 164.4 (q, ^1^*J*_C–B_ = 50 Hz; *i*-*C*_Ar_ (BPh_4_)), 148.8 (d, ^3^*J*_C–P_ = 23 Hz; *i*-*C*_Ar_ (*p*-anisyl)), 136.3 (br q, ^3^*J*_C–B_ = 1 Hz; *m*-*C*_Ar_ (BPh_4_)), 126.7 (d, ^5^*J*_C–P_ = 1 Hz; *m*-*C*_Ar_ (*p*-anisyl)), 126.0 (q, ^2^*J*_C–B_ = 3 Hz; *o*-*C*_Ar_ (BPh_4_)), 122.2 (s; *p*-*C*_Ar_ (BPh_4_)), 115.3 (s; *o*-*C*_Ar_ (*p*-anisyl)), 107.6 (s; *i*-*C*_Ar_ (*p*-cymene)), 102.7 (s; *p*-*C*_Ar_ (*p*-cymene)), 98.6 (d, ^2^*J*_C–P_ = 3 Hz, *C*_Ar_ (*p*-cymene)), 89.9 (d, ^2^*J*_C–P_ = 7 Hz; *C*_Ar_ (*p*-cymene)), 89.2 (s; *C*_Ar_ (*p*-cymene)), 86.0 (d, ^2^*J*_C–P_ = 3 Hz; *C*_Ar_ (*p*-cymene)), 57.0 (s; *p*-OCH_3_ (*p*-anisyl)), 42.9 (d, ^1^*J*_C–P_ = 4 Hz; *C*(CH_3_)_3_), 42.2 (d, ^1^*J*_C–P_ = 10 Hz; *C*(CH_3_)_3_), 31.6 (s; *C*(CH_3_)_2_ (*p*-cymene)), 31.4 (d, ^2^*J*_C–P_ = 4 Hz; C(*C*H_3_)_3_), 28.9 (d, ^1^*J*_C–P_ = 3 Hz; C(*C*H_3_)_3_), 23.0 (s; C(*C*H_3_)_2_ (*p*-cymene)), 22.3 (s; C(*C*H_3_)_2_ (*p*-cymene)), 18.8 (s; *p*-CH_3_ (*p*-cymene)). ^31^P{^1^H} (162.0 MHz, CD_2_Cl_2_): δ 67.4 (s). ^31^P (202.4 MHz, CD_2_Cl_2_): δ 67.4 (m). ^11^B{^1^H} (128.4 MHz, CD_2_Cl_2_): δ −6.6 (s). UV–vis (DCM, nm) λ_max_: 392 (s), 460–650 (br w). ESI-HRMS *m*/*z* for C_25_H_39_ClN_2_OPRu [M – BPh_4_]^+^: calcd (found) 556.1551 (556.1537), 555.1526 (555.1522), 554.1563 (554.1558), 553.1533 (553.1530), 552.1548 (552.1547), 551.1536 (551.1534), 550.1544 (550.1541), 549.1541 (549.1538), 548.1550 (548.1545), 547.1544 (547.1533), 545.1564 (545.1562). ESI-HRMS *m*/*z* for C_24_H_20_B[BPh_4_]^−^: calcd (found) 321.1727 (321.1715), 320.1694 (320.1700), 319.1662 (319.1660), 318.1694 (318.1690). IR (cm^–1^) ν_max_: 3054 (w), 2968 (w), 1593 (m, N=N), 1579 (m). Elemental Analysis: calcd (found) C% 67.62 (67.56), H% 6.83 (6.94), N% 3.22 (3.01).

### Procedure for In Situ ReactIR Measurements

Standard addition was used to quantify in situ FTIR reaction data according to literature procedures without the need for offline sampling and analysis.^35^ For this, acetophenone was added in two portions, allowing for five consecutive scans after each addition, and the average of these two calibrations was reported. The average temperature during the reactions was recorded by the FTIR probe. Changes in the IR were monitored over time via the solvent abstraction feature of the iCIR software. To select an appropriate peak to monitor, IR spectra of acetophenone, acetone, and phenylethanol in IPA were taken. Using the iCIR software, the reference spectra were stacked, and the most isolated peaks were selected for monitoring (Figure S102). The acetophenone was monitored using the height of the signal at 1267 cm^–1^ relative to a two-point baseline (1284 and 1252 cm^–1^). Due to a lack of isolated peaks for phenylethanol, we were unable to quantify the formation of the product by FTIR. Therefore, an aliquot of the reaction was taken for analysis by qNMR to confirm the presence and yield of phenylethanol.

### Procedure for Transfer Hydrogenation

A vial, equipped with stirrer bar and the ReactIR probe, was placed in an aluminum heating block, set at 80 °C, and charged with IPA (2.0 mL). Three consecutive spectra were collected after the addition of IPA. A stock solution of acetophenone (1 M in IPA) was added in two 0.5 mL portions (overall 1.0 mL, 1.0 mmol, 120.3 mg) to give 3.0 mL of solution. Five consecutive spectra were taken after both additions of stock acetophenone before **4-OMe** (1 mol %, 0.01 mmol, 5.9 mg) or **5-OMe** (1 mol %, 0.01 mmol, 8.7 mg) was added to initiate the reaction. Addition of the solid required the reaction vial to be briefly lowered from the FTIR probe; this FTIR data point was removed for clarity. Parafilm was secured around the vial and FTIR probe to minimize evaporation, and the reaction was stirred and heated at 80 °C for 5 h with spectra collected every 60 s until the reaction appeared to end by visual analysis. After monitoring by FTIR had ceased, the reaction was left stirring at 80 °C overnight (16 h) and an aliquot, filtered through Celite, was analyzed by ^1^H NMR spectroscopy.

### General Procedure for Transfer Hydrogenation (NMR Scale)

A premixed solution of hydrogen source (IPA or formic acid, 0.5 mL) and substrate (acetophenone or benzaldehyde, 0.17 mmol, 1 equiv) was added to a vial containing **5-OMe** (1 mol%, 1.7 μmol, 1.5 mg). The reaction vial was capped, parafilmed, and stirred at 80 °C in a sand bath overnight (16 h), after which an aliquot, filtered through Celite, was analyzed by ^1^H NMR spectroscopy.

### Computational Details

All computational details can be found in the Supporting Information. Cartesian coordinates are provided in a separate .xyz file.

## Supporting Information Available

Full characterization spectra for **1-OMe**, **2-R** (R = OMe, Me, H, F, CF_3_), **4-OMe**, and **5-OMe**; ^31^P{^1^H} NMR spectra of **3-R** (R = NMe_2_, OMe, Me, H, F, CF_3_); crystallographic data for **1-OMe**, **2-R** (R = OMe, Me, H, F, CF_3_), **4-OMe**, and **5-OMe**; ReactIR spectra; computational details (PDF)
Cartesian coordinates of all computed structures (XYZ)

## Supplementary Material

om4c00302_si_001.pdf

om4c00302_si_002.xyz
