# Supplementary material for: Tuning the Electronic Properties of Azophosphines as Ligands and Their Application in Base-Free Transfer Hydrogenation Catalysis
Source: Organometallics. 2024 Sep 6;43(20):2674–85. doi: 10.1021/acs.organomet.4c00302 (PMC11523462; doi:10.1021/acs.organomet.4c00302)
Supplement: Supplementary file 1 — om4c00302_si_001.pdf [file om4c00302_si_001.pdf]

Supporting Information for

Tuning the Electronic Properties of Azophosphines as Ligands and their Application in Base-Free Transfer Hydrogenation Catalysis

Emma J. Jordan, Ethan D. E. Calder, Bethan L. Greene, Holly V. Adcock, Louise Male, Paul W. Davies, Andrew R. Jupp*

a.jupp@bham.ac.uk

[a]School of Chemistry, University of Birmingham, Edgbaston, Birmingham, B15 2TT, UK

Table of Contents

S1. Synthetic Details 2
S1.1 General Information 2
S2. Characterisation Spectra 3
S2.1. NMR Spectra 3
S2.2. UV/Vis Spectra 34
S2.3. High-Resolution Mass Spectra 46
S2.4. IR Spectra 52
S3. Single Crystal X-ray Diffraction 59
S3.1. Tables of Crystallographic Data and Structure Refinement 59
S3.2. Crystal Structure Determinations 60
S4. Catalysis 62
S4.1. Spectra 62
S5. Computational Details 66
S5.1. General Information for Optimisations of Free Azophosphines 66
S5.2. Computational Data for Free Azophosphines 66
S5.3. General Information for Optimisations of Ru-Azophosphine Complexes 67
S5.4. Computational Data for Ru-Azophosphine Complexes 68
References 70

S1. Synthetic Details

S1.1 General Information

Thin layer chromatography (TLC) analysis was performed using Machery-Nagel aluminium-backed silica plates. Spots were visualised by the quenching of ultraviolet light. All flash column chromatography was performed using Fluorochem 60. silica gel (particle size 40–63 µm) with a column of appropriate size. All glassware and Teflon-coated stirrer bars were dried in a 180 °C oven overnight prior to use, unless otherwise stated. All molecular sieves are 3 Å and purchased from VWR chemicals and were activated by heating at 400 °C under vacuum prior to use. Unless otherwise stated, degassing refers to three freeze-pump-thaw cycles.

Commercial reagents were purchased, suitably stored as specified by the supplier, and used as received, unless otherwise stated, from Sigma-Aldrich (*p*-anisidine, 99%; aniline, 99.5%; *p*-(trifluoromethyl) aniline, 99%; *sec*-butyllithium, 1.42 M in cyclohexane; borane-di(*tert*-butyl)phosphine complex, 94%; pyrrolidine, anhydrous, > 99.5%, stored in an air-tight ampoule; sodium tetraphenylborate, ≥99.5%), Acros Organics (*N*,*N*-dimethyl-*p*-phenylenediamine, 97%; di-µ-chlorobis(*p*-cymene)chlororuthenium(II), 98%, stored and handled in the glovebox) or Alfa Aesar (*p*-toluidine, 99+%; *p*-fluoroaniline, 99%; sodium nitrite, 97%; tetrafluoroboric acid, ca. 50% w/w aq. sol.) Solvents were purchased and used as received, unless otherwise stated, from Sigma-Aldrich (hexane, puriss, p.a., ACS reagent, reag. Ph. Eur., 99+ (GC), degassed and stored in air-tight ampoules over 3 Å molecular sieves; diethyl ether, ACS reagent, 99.8%; acetonitrile, suitable for HPLC, gradient grade, 99.9%; acetonitrile-d_3, 99.8 atom % D, contains 0.03% (v/v) TMS; chloroform-*d*, 99.8 atom % D, dried by overnight reaction with CaH_2, degassed and stored in an air-tight ampoule over 3 Å molecular sieves; toluene-d_8, 99.6 atom % D, dried by overnight reaction with CaH_2, degassed and stored in an air-tight ampoule over 3 Å molecular sieves; dichloromethane-d_2, 99.5 atom % D, dried by overnight reaction with CaH_2, degassed and stored in an air-tight ampoule over 3 Å molecular sieves; bromobenzene-d_5, 99.5 atom % D), Fisher Scientific (*iso*-octane, laboratory reagent grade; dichloromethane (DCM), 99.8%, HPLC grade) or Acros Organics (chlorobenzene, 99.6%, ACS reagent, degassed and stored in an air-tight ampoule over 3 Å molecular sieves). Toluene was obtained from the laboratory solvent purification system, degassed, and stored in air-tight ampoules over 3 Å molecular sieves. THF was obtained from the laboratory solvent purification system, degassed, dried over Na/benzophenone, and stored in air-tight ampoules over 3 Å molecular sieves. Deionised water was obtained from an Elga DV35 Purelab. For recrystallisations and as eluent for column chromatography, hexane was used as received.

S2. Characterisation Spectra

S2.1. NMR Spectra

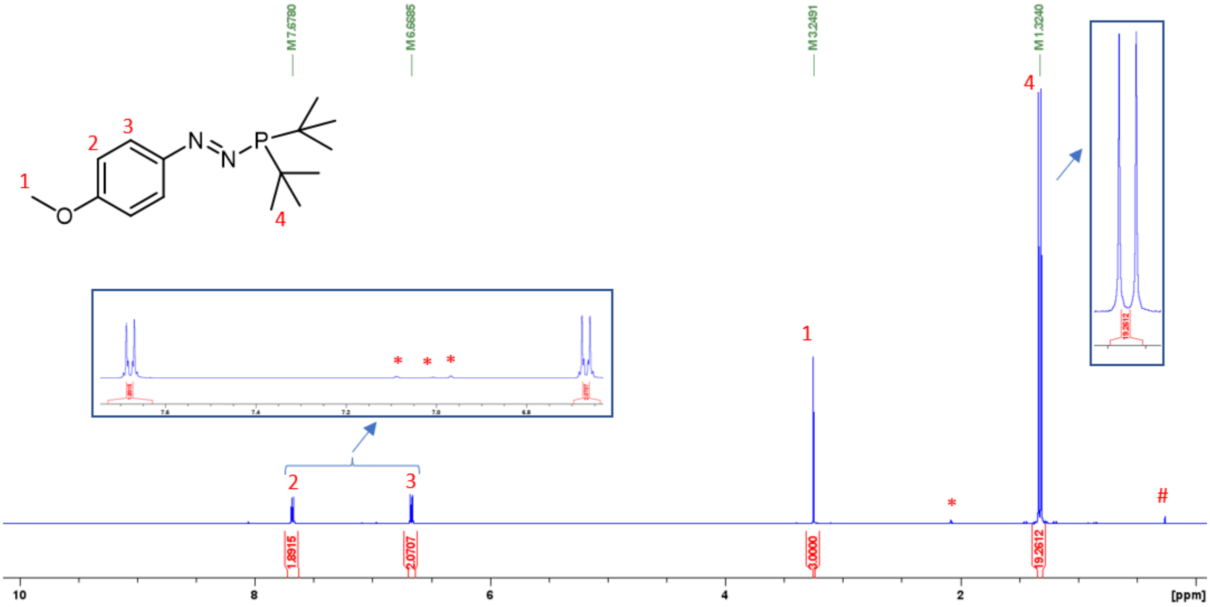

Figure S1. ^{1}H NMR spectrum of **1-OMe** in toluene-d_8. * = residual toluene-d_7H, # = silicon grease.

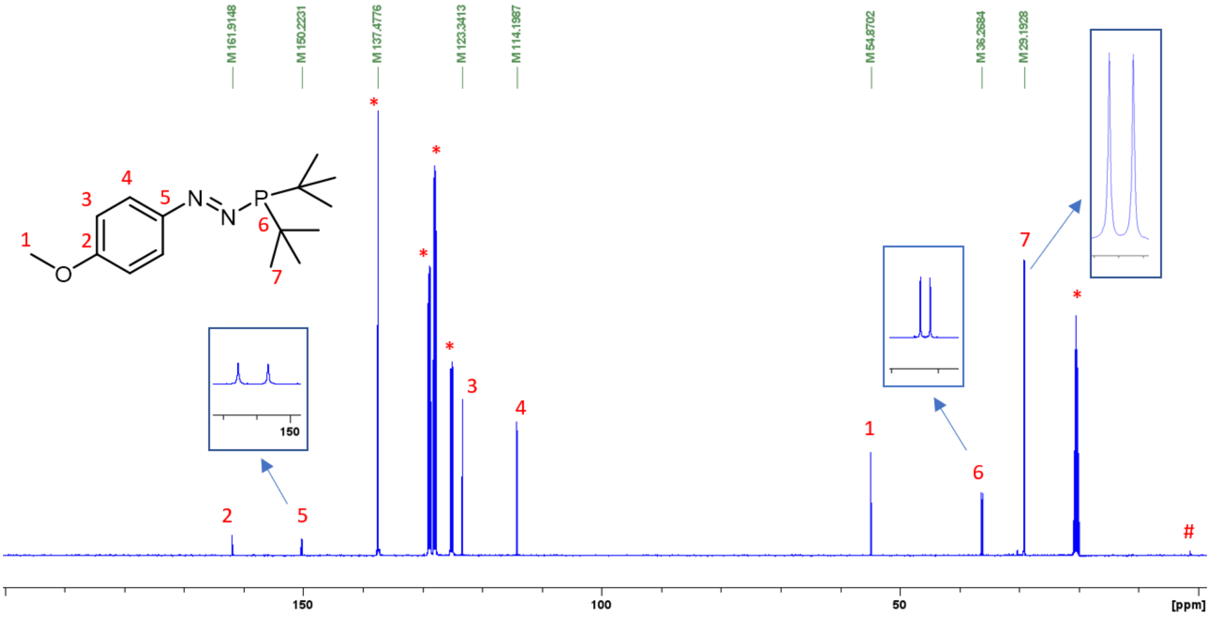

Figure S2. $^{13}C\{^{1}H\}$ NMR spectrum of **1-OMe** in toluene-d_8. * = toluene-d_8, # = silicon grease.

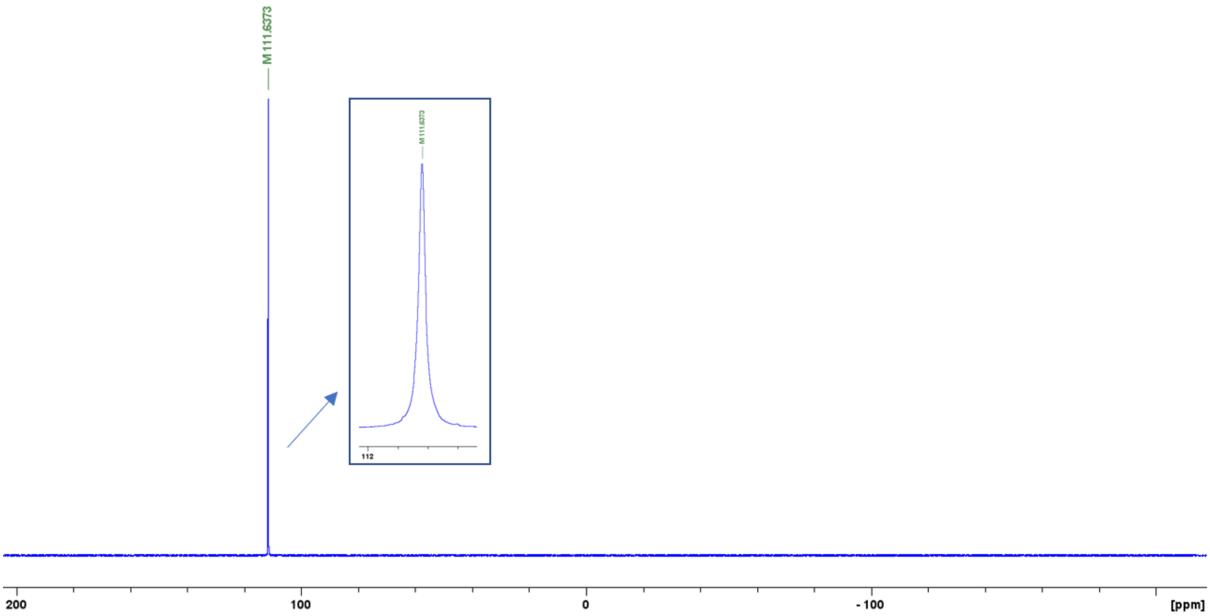

Figure S3. $^{31}P\{^{1}H\}$ NMR spectrum of **1-OMe** in toluene-d$_8$.

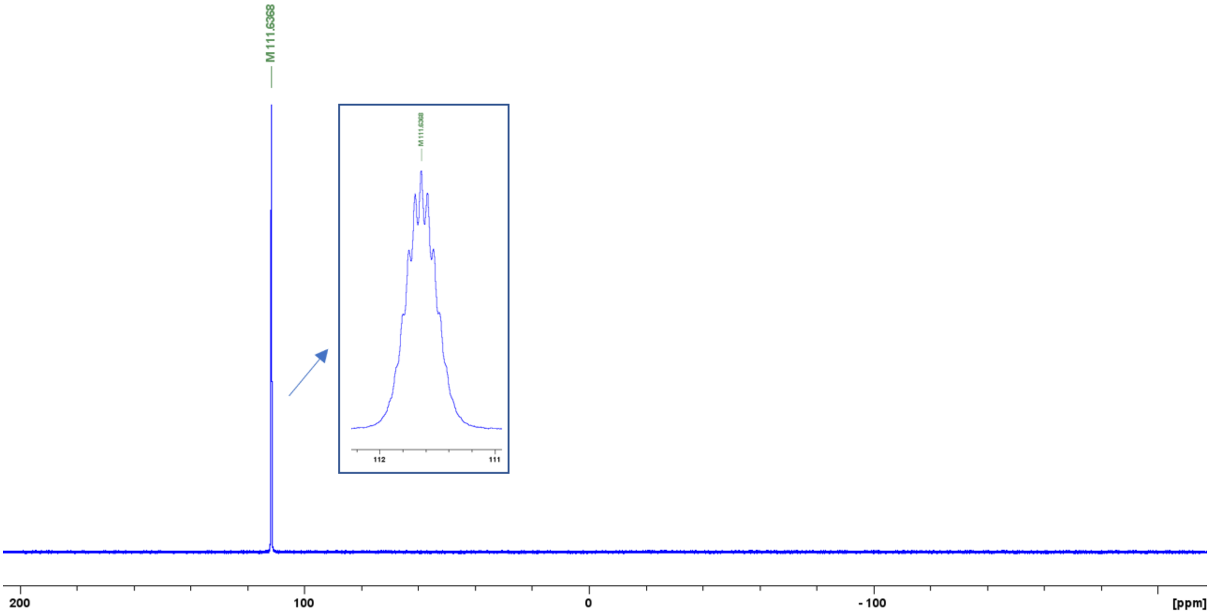

Figure S4. ^{31}P NMR spectrum of **1-OMe** in toluene-d$_8$.

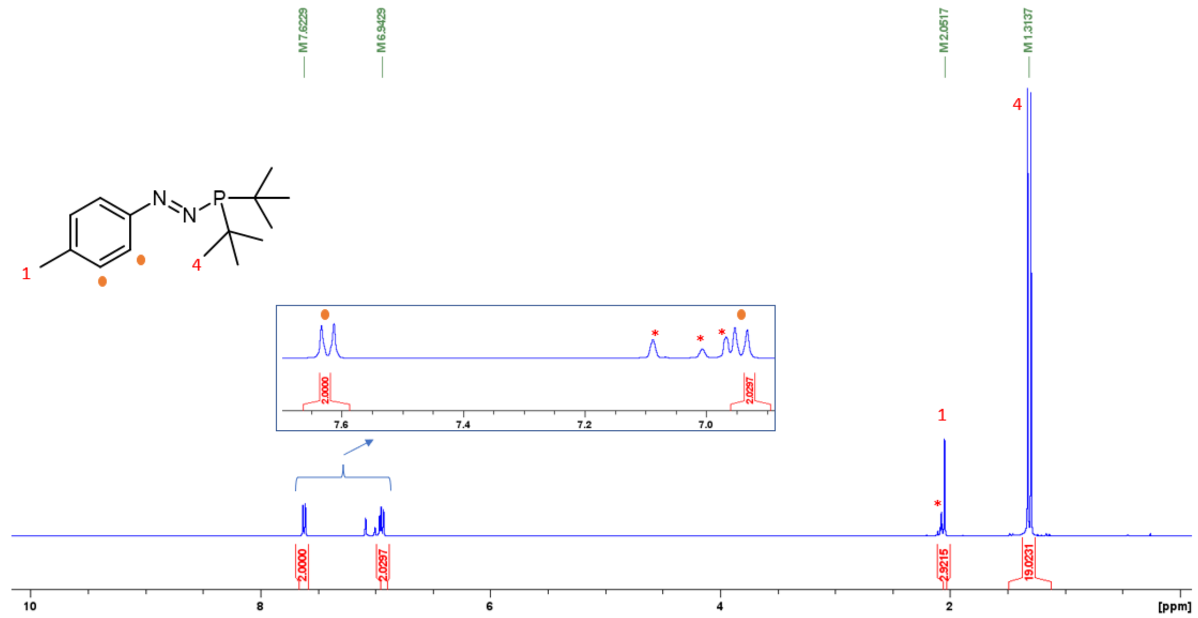

Figure S5. ^{1}H NMR spectrum of **1-Me** in toluene-d_8. * = residual toluene-d_7H.

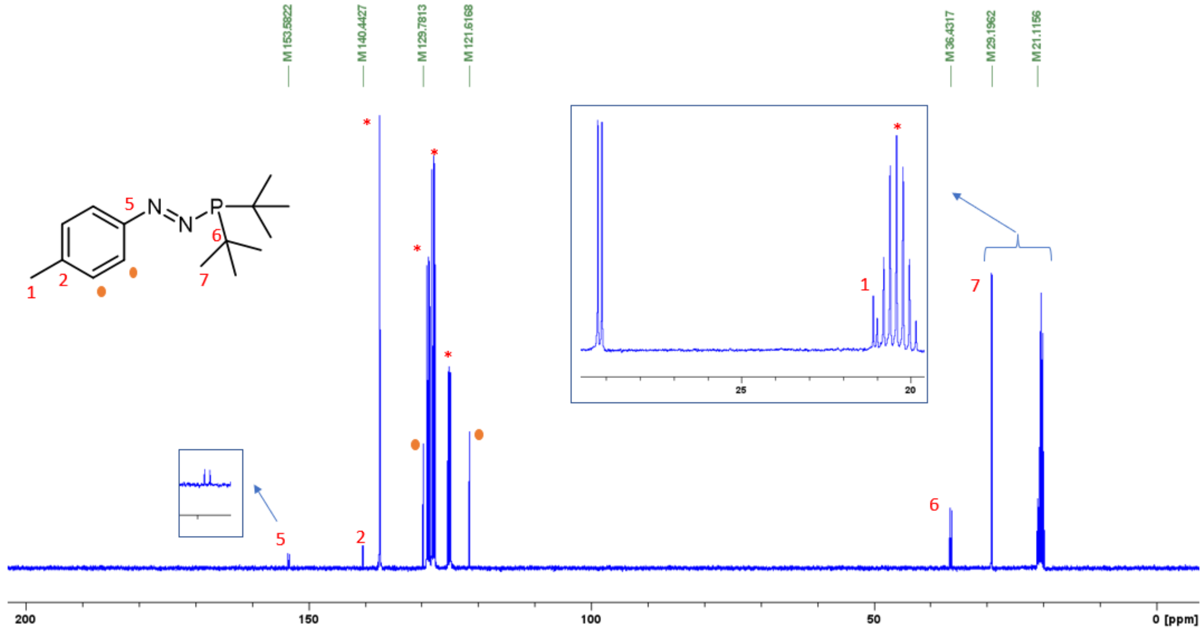

Figure S6. ^{13}C{^{1}H} NMR spectrum of **1-Me** in toluene_d_8. * = toluene-d_8.

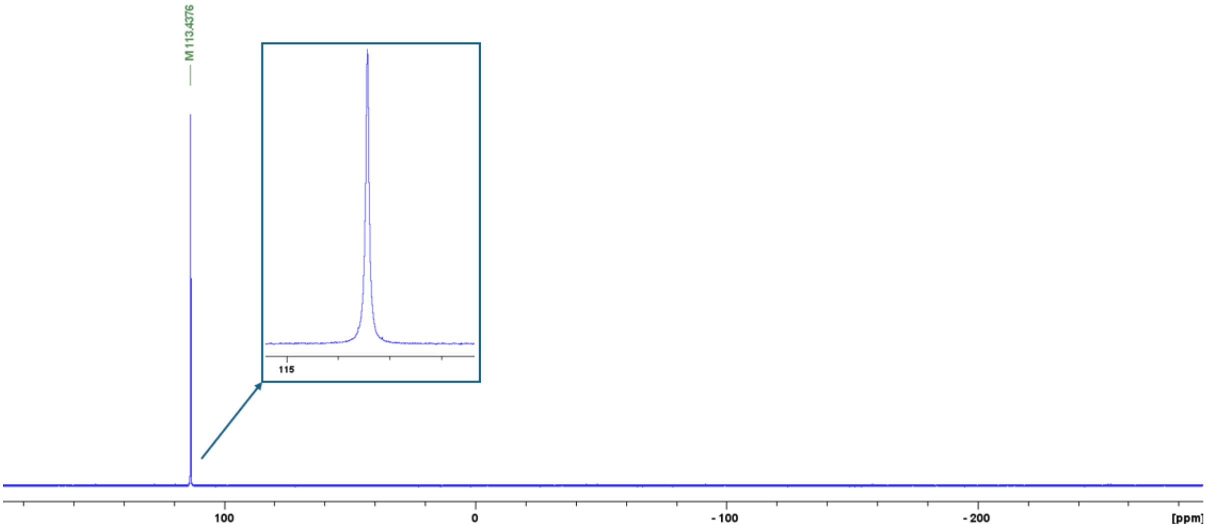

Figure S7. $^{31}P\{^{1}H\}$ NMR spectrum of **1-Me** in toluene-d8.

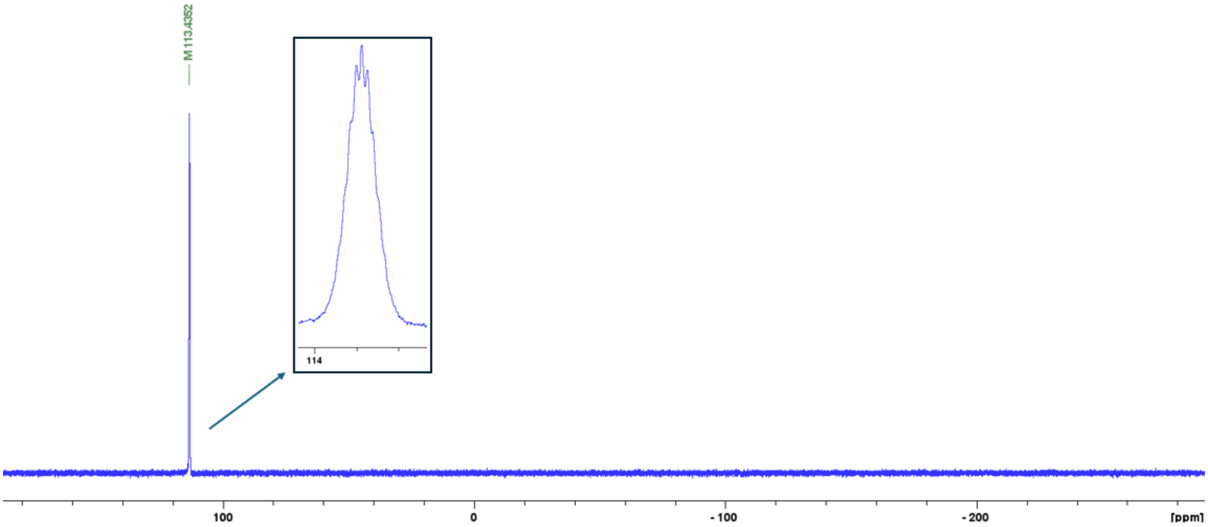

Figure S8. ^{31}P NMR spectrum of **1-Me** in toluene-d8.

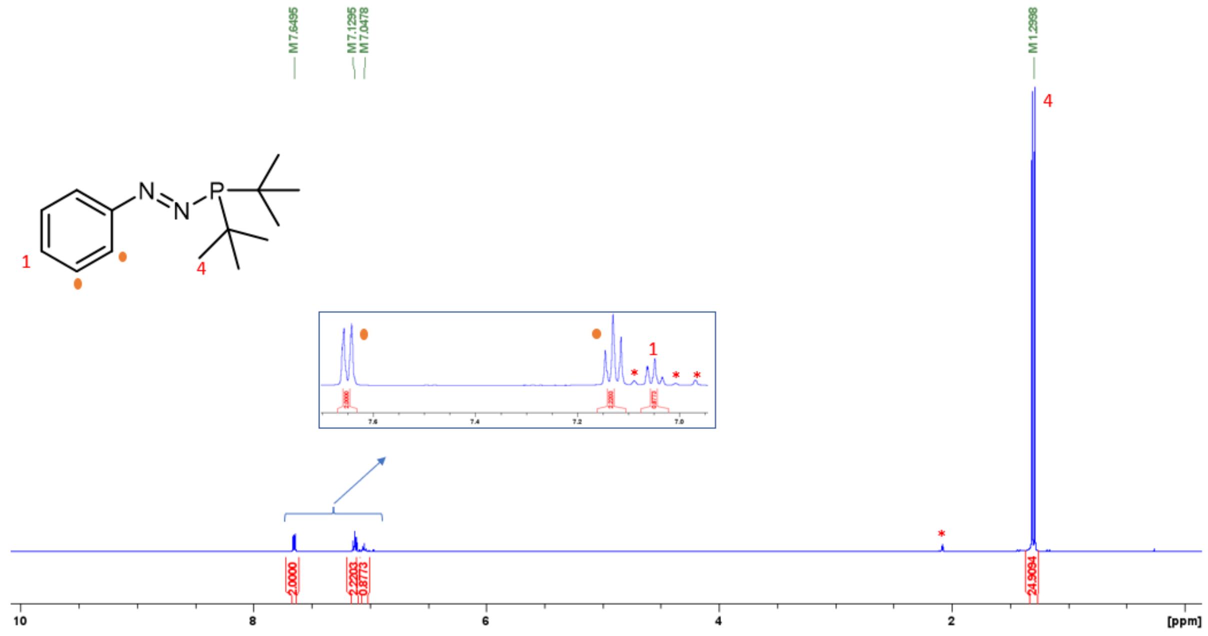

Figure S9. ^{1}H NMR spectrum of **1-H** in toluene-d_8. * = residual toluene-d_7H.

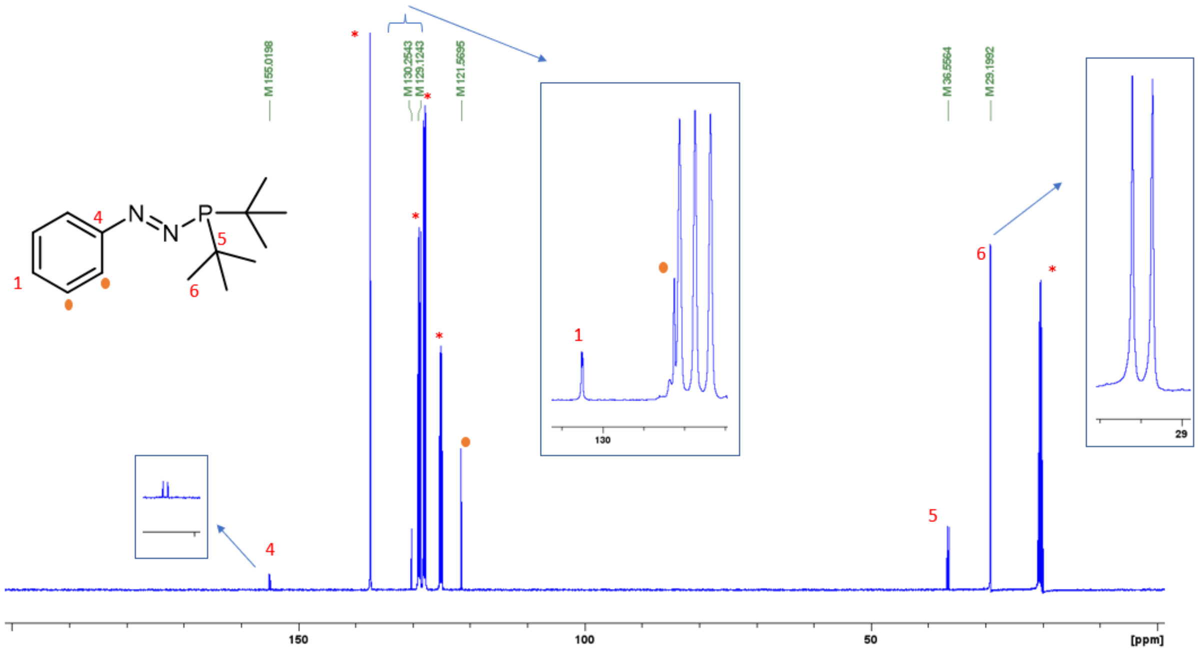

Figure S10. $^{13}C\{^{1}H\}$ NMR spectrum of **1-H** in toluene-d_8. * = toluene-d_8.

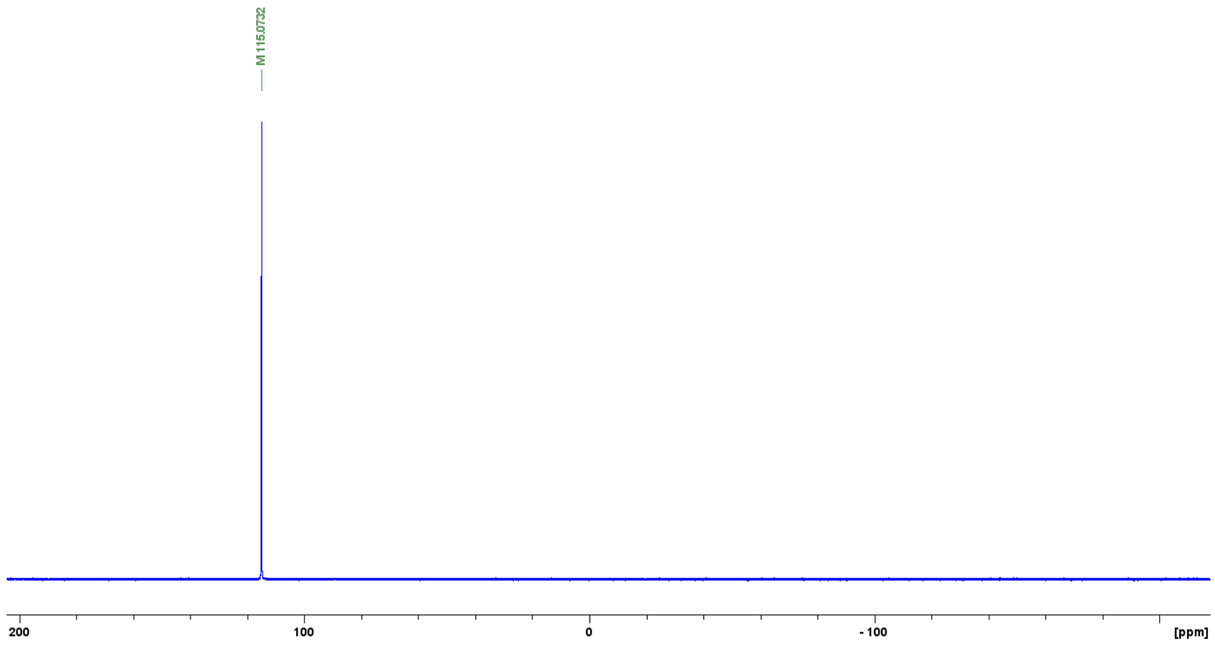

Figure S11. $^{31}P\{^{1}H\}$ NMR spectrum of **1-H** in toluene-d_8.

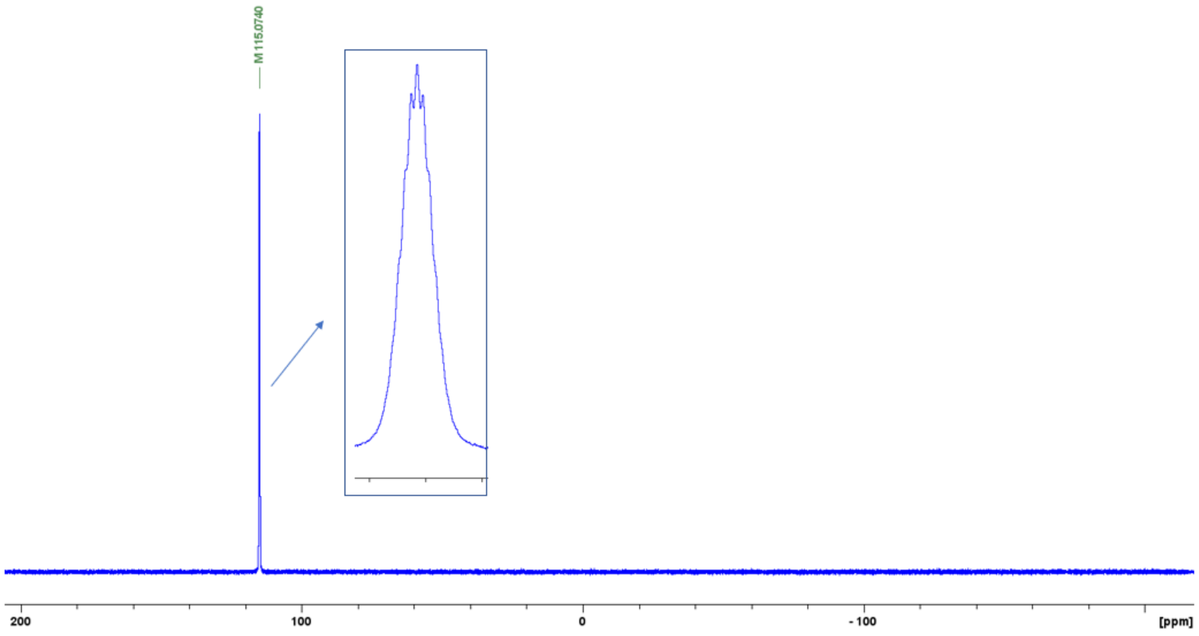

Figure S12. ^{31}P NMR spectrum of **1-H** in toluene-d_8.

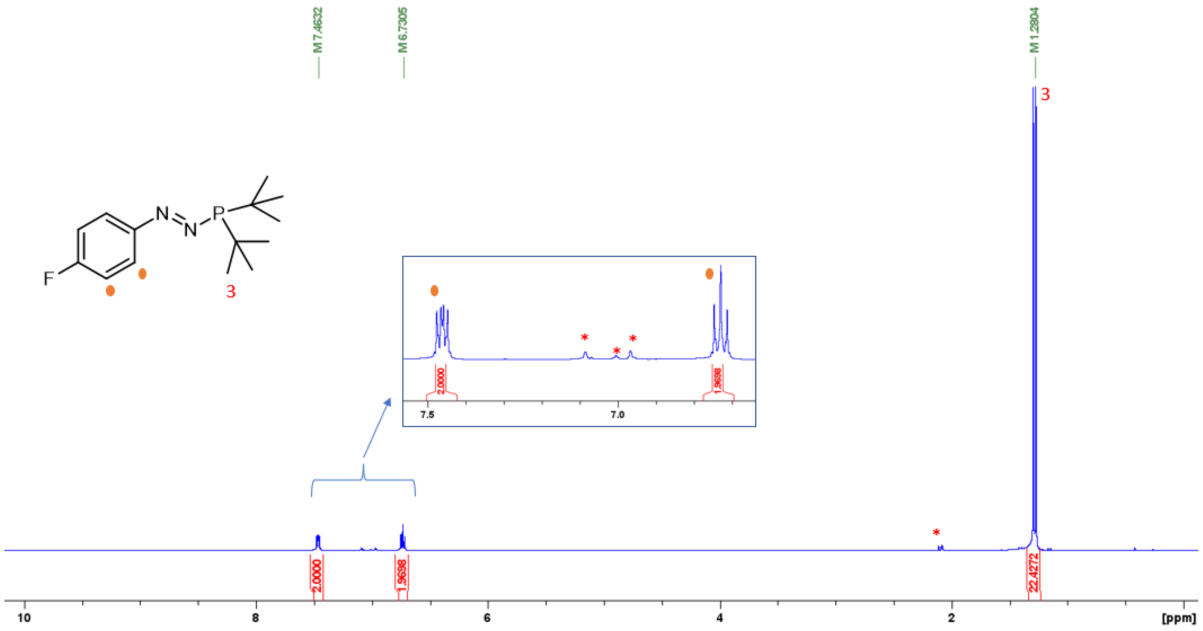

Figure S13. ^{1}H NMR spectrum of **1-F** in toluene-d_8. * = residual toluene-d_7H.

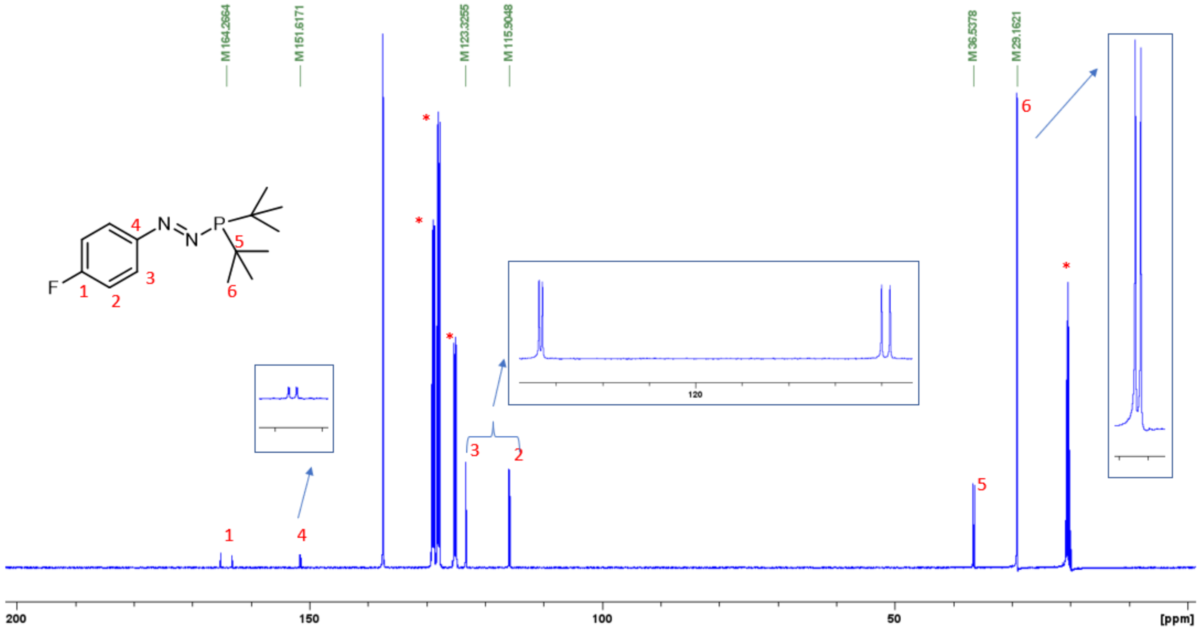

Figure S14. ^{13}C{^{1}H} NMR spectrum of **1-F** in toluene-d_8. * = toluene-d_8.

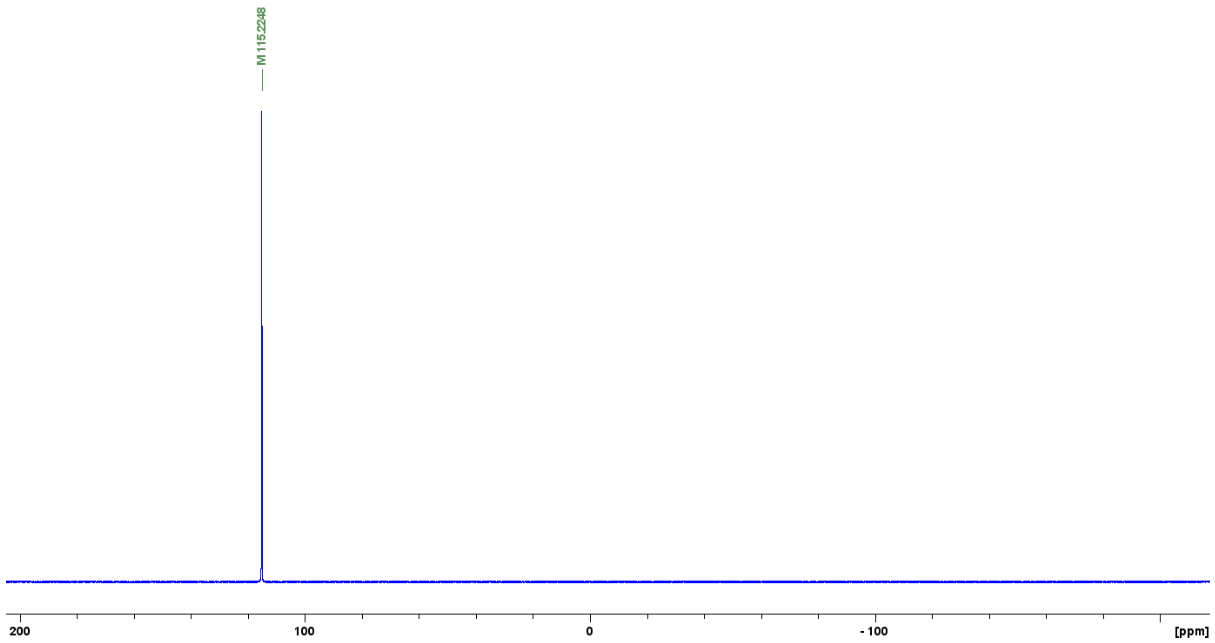

Figure S15. $^{31}P\{^{1}H\}$ NMR spectrum of **1-F** in toluene-d_8.

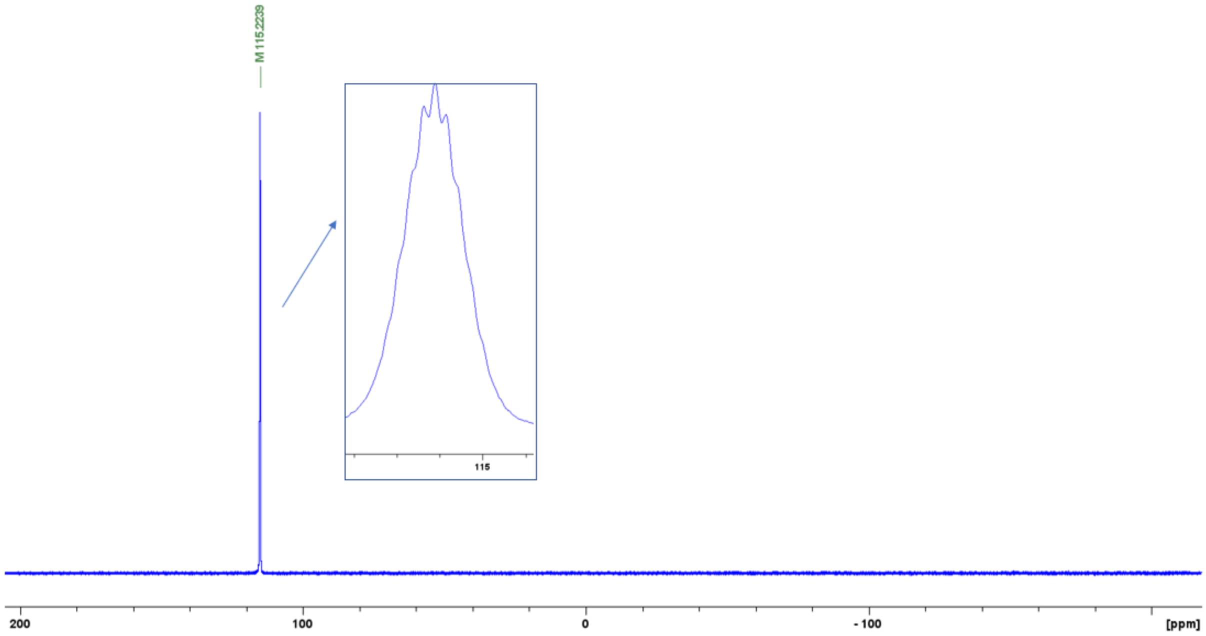

Figure S16. ^{31}P NMR spectrum of **1-F** in toluene-d_8.

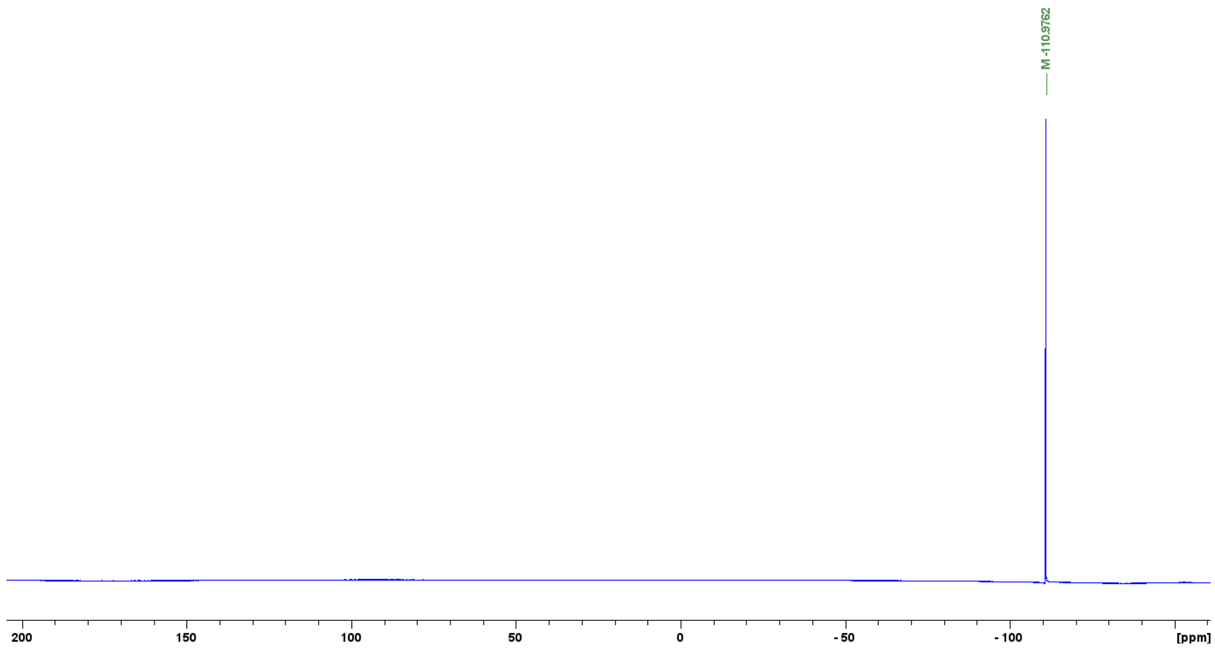

Figure S17. $^{19}F\{^1H\}$ NMR spectrum of **1-F** in toluene-d_8.

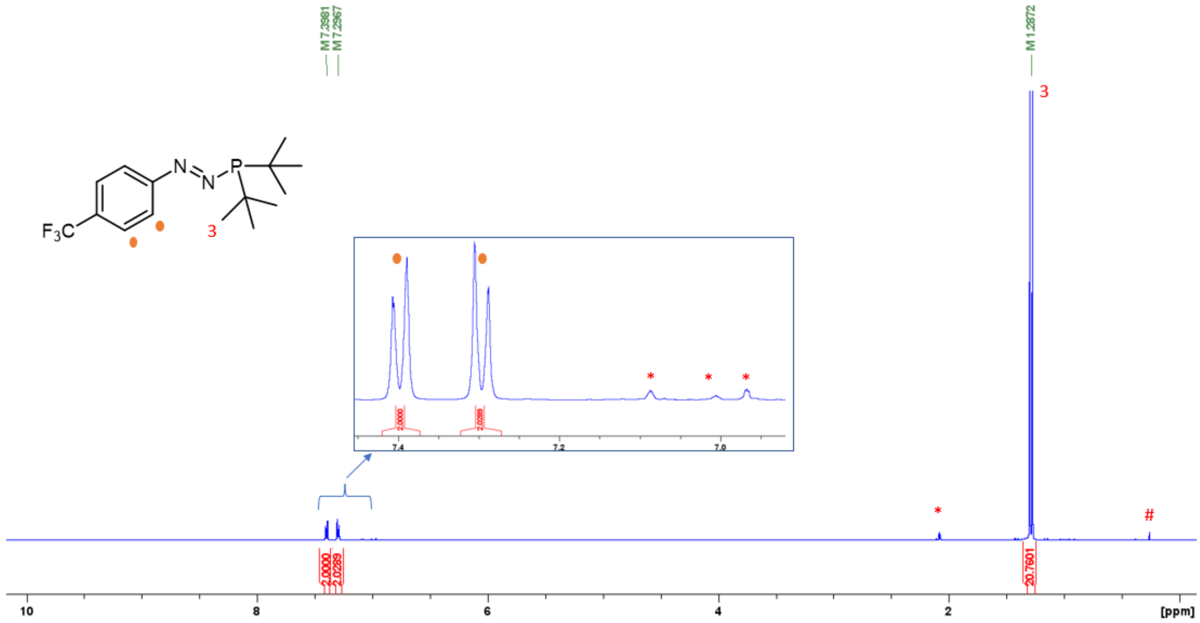

Figure S18. ^1H NMR spectrum of **1-CF$_3$** in toluene-d_8. * = residual toluene-d_7H, # = silicone grease.

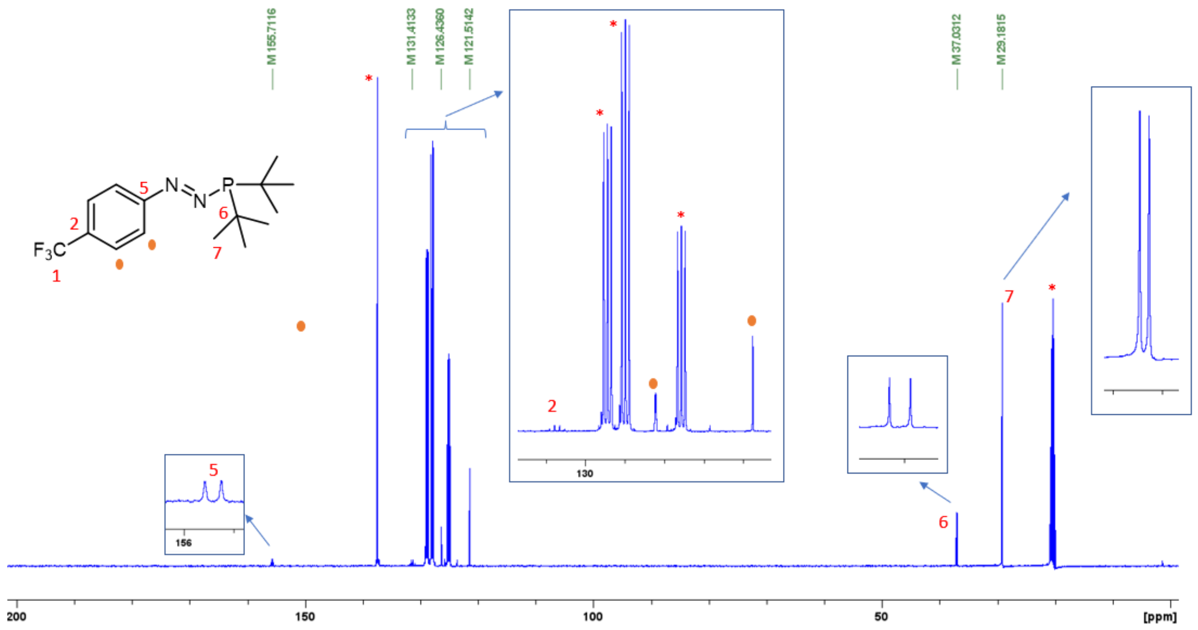

Figure S19. ^{31}C{^{1}H} NMR spectrum of **1-CF$_3$** in toluene-d$_8$. * = toluene-d$_8$.

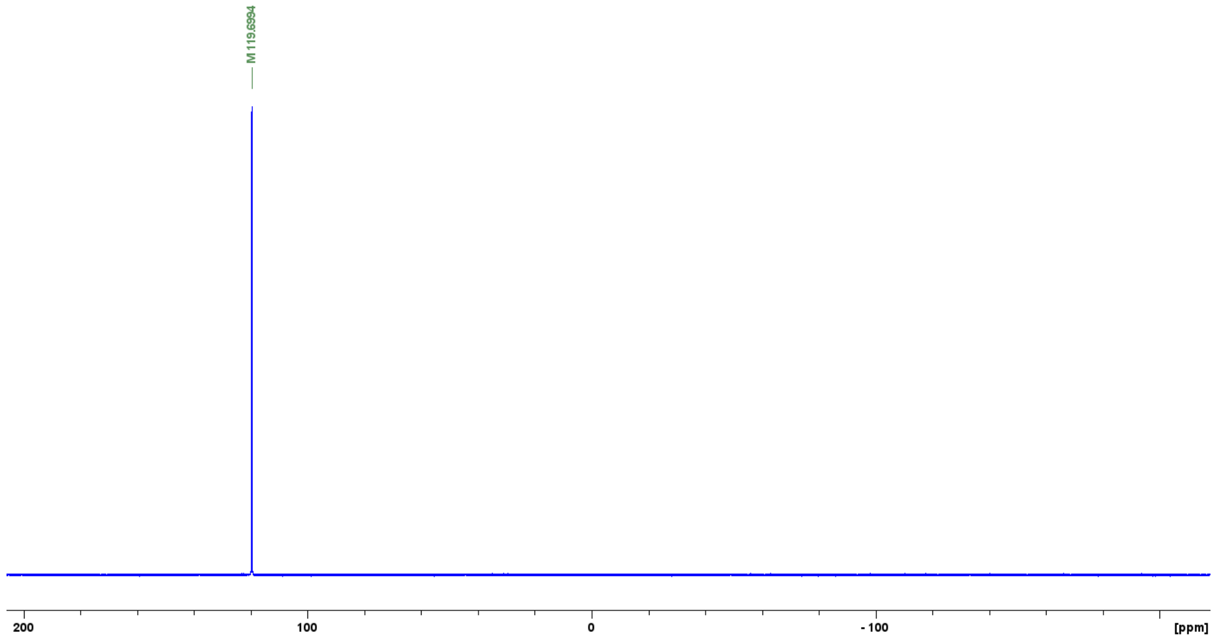

Figure S20. ^{31}P{^{1}H} NMR spectrum of **1-CF$_3$** in toluene-d$_8$.

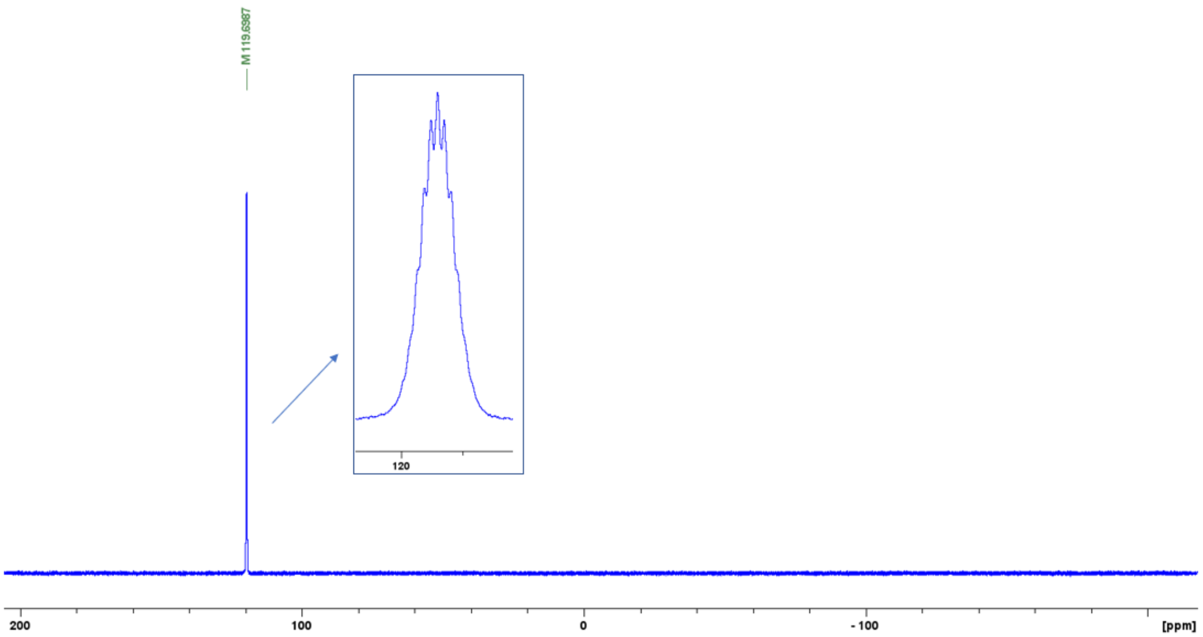

Figure S21. ^{31}P NMR spectrum of **1-CF$_3$** in toluene-d$_8$.

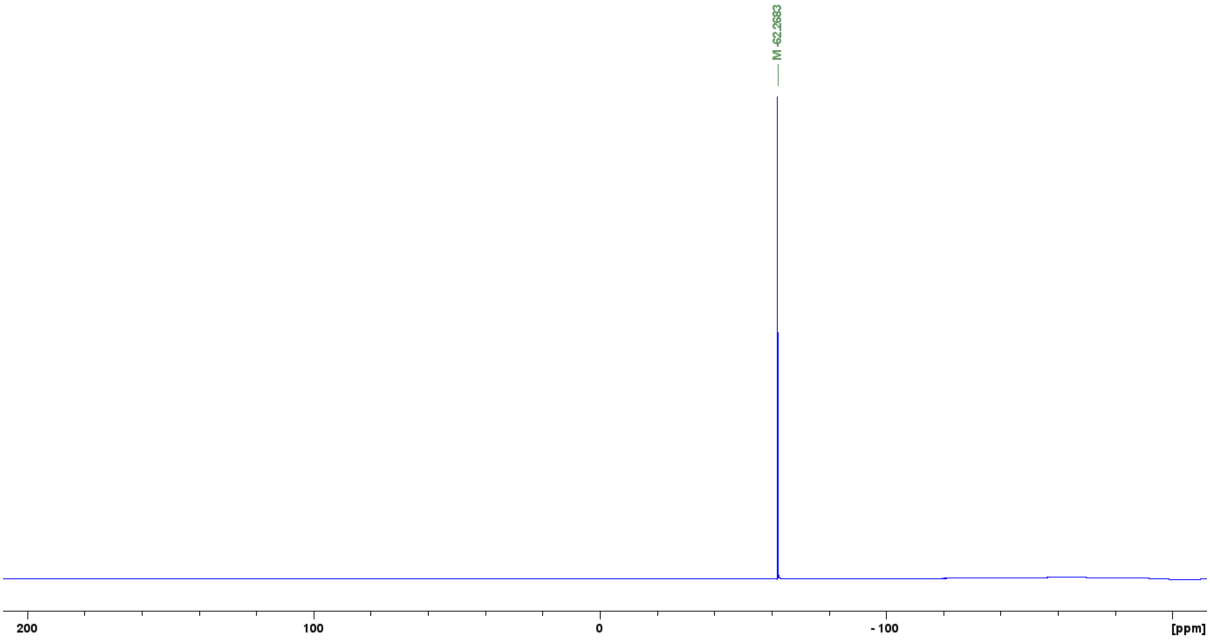

Figure S22. $^{19}F\{^{1}H\}$ NMR spectrum of **1-CF$_3$** in toluene-d$_8$.

.

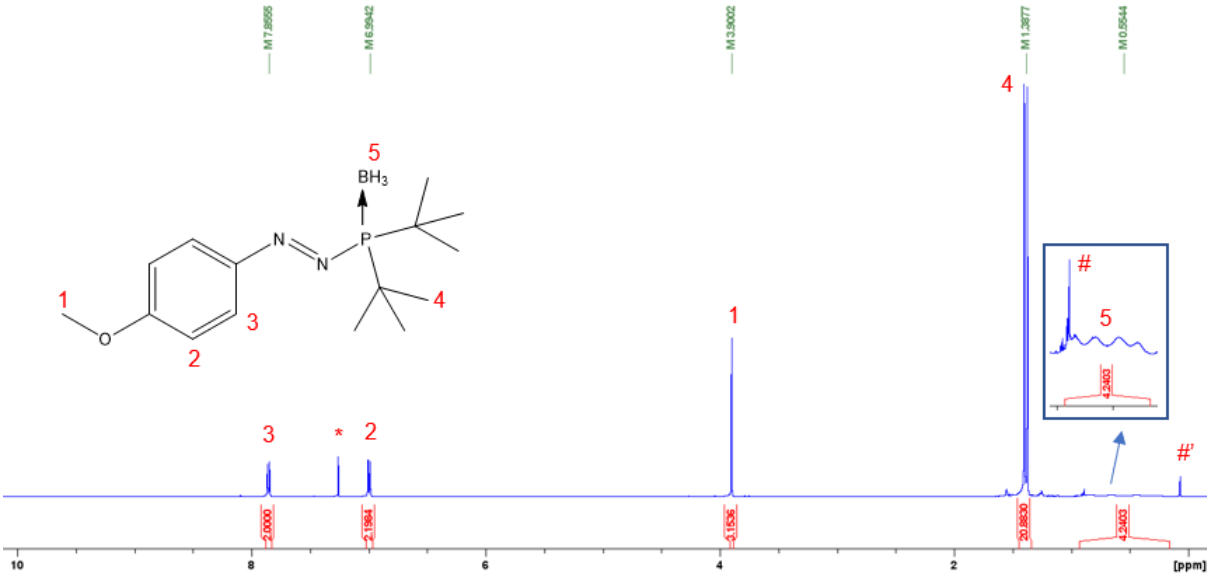

Figure S23. ^{1}H NMR spectrum of **2-OMe** in $CDCl_3$. * = residual $CHCl_3$; # = residual hexane; #' = silicon grease.

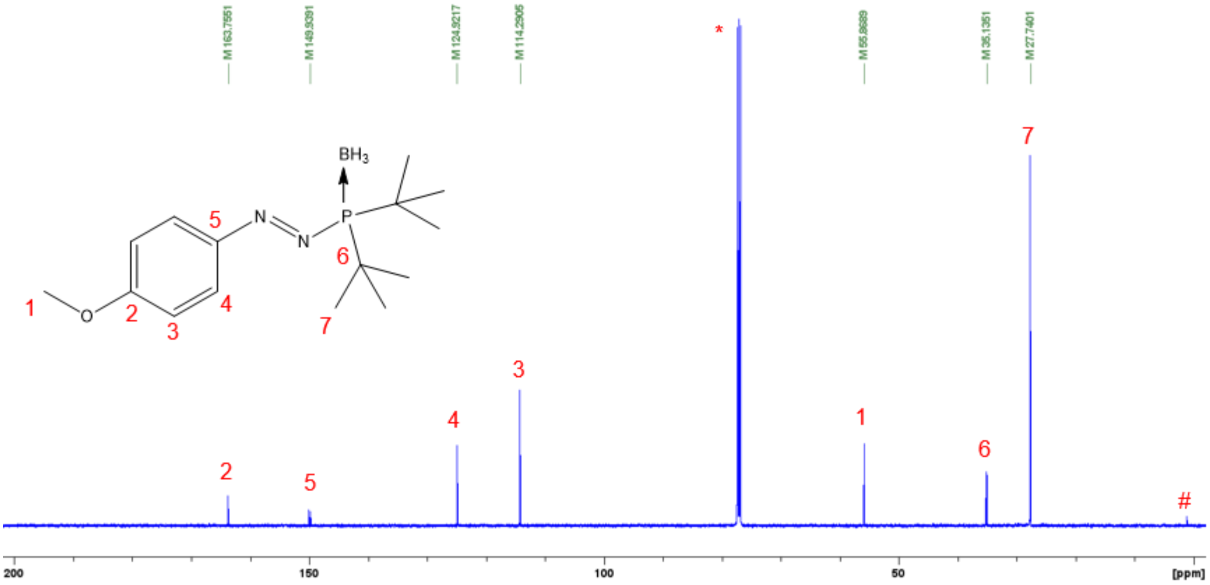

Figure S24. $^{13}C\{^{1}H\}$ spectrum of **2-OMe** in $CDCl_3$. * = $CDCl_3$; # = silicon grease.

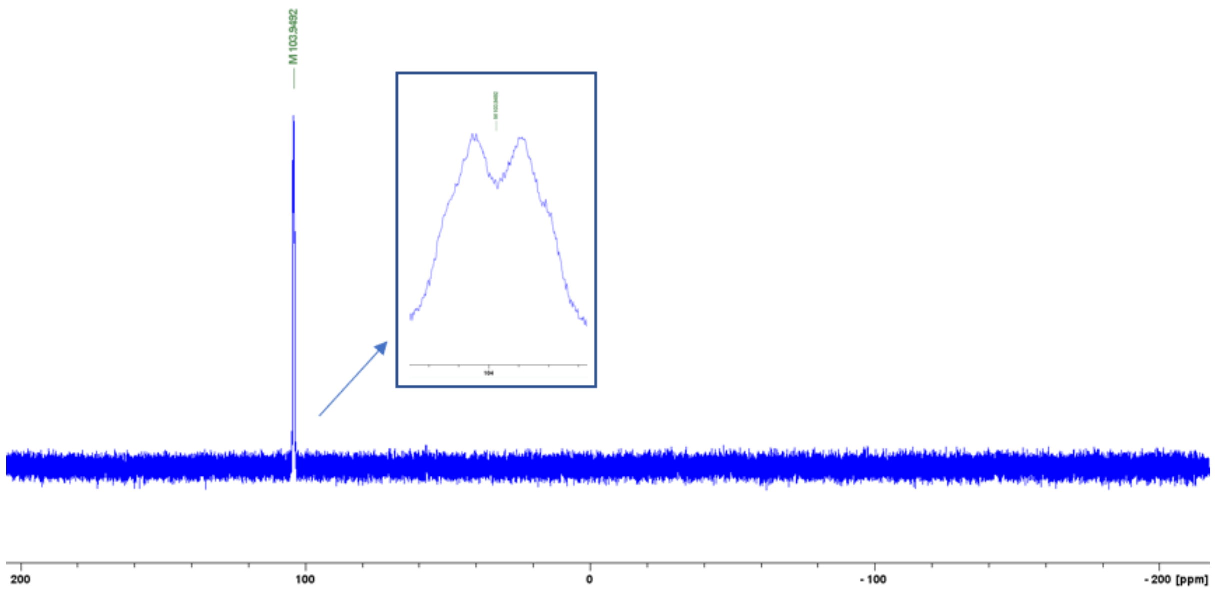

Figure S25. $^{31}P\{^1H\}$ spectrum of **2-OMe** in $CDCl_3$.

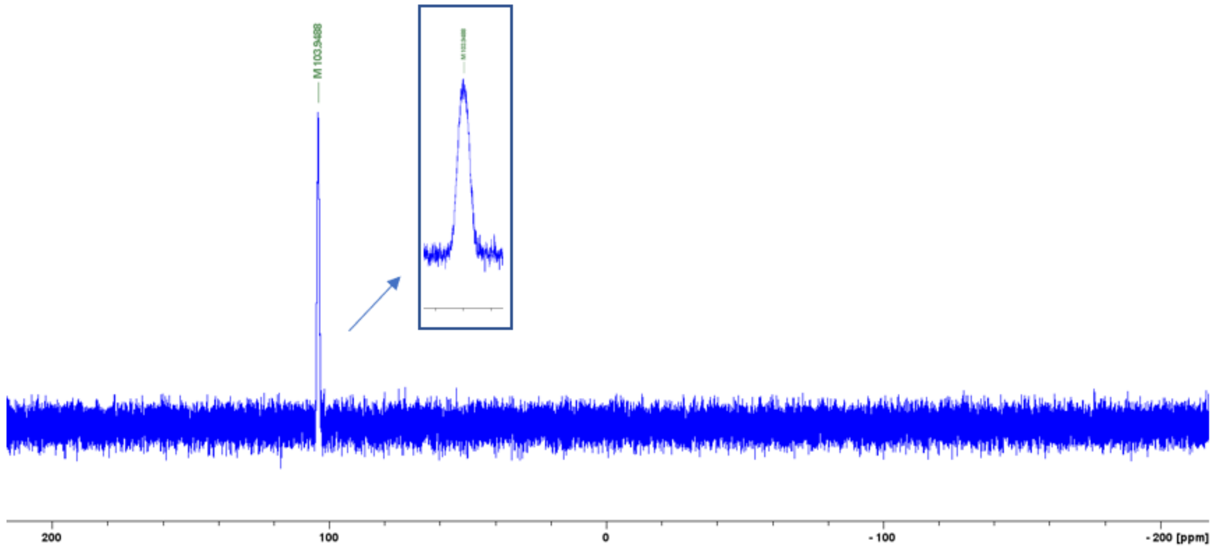

Figure S26. ^{31}P spectrum of **2-OMe** in $CDCl_3$.

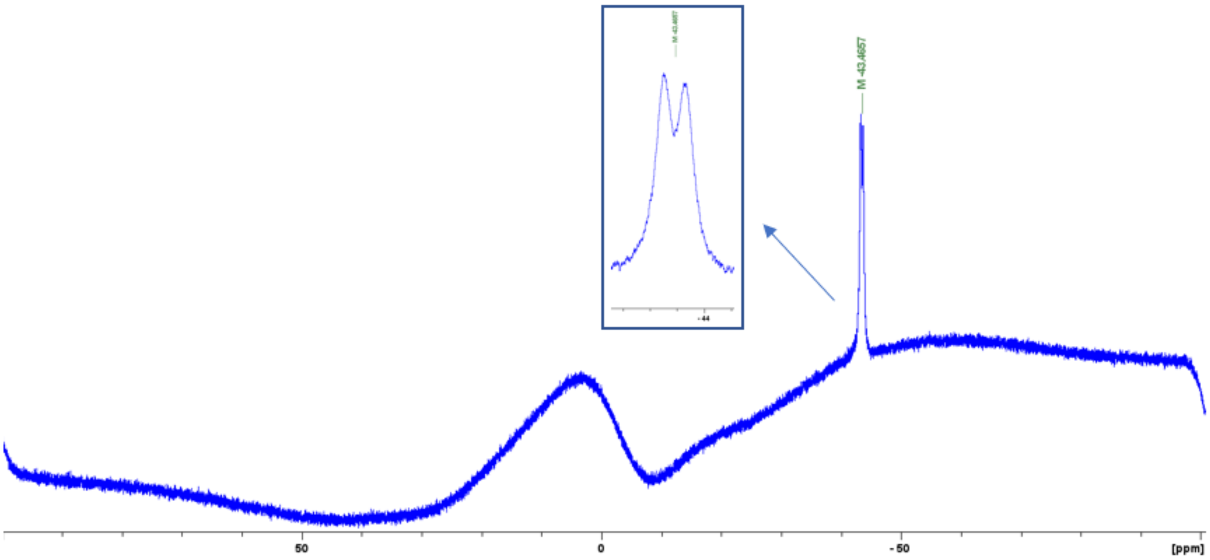

Figure S27. $^{11}B\{^1H\}$ spectrum of **2-OMe** in $CDCl_3$.

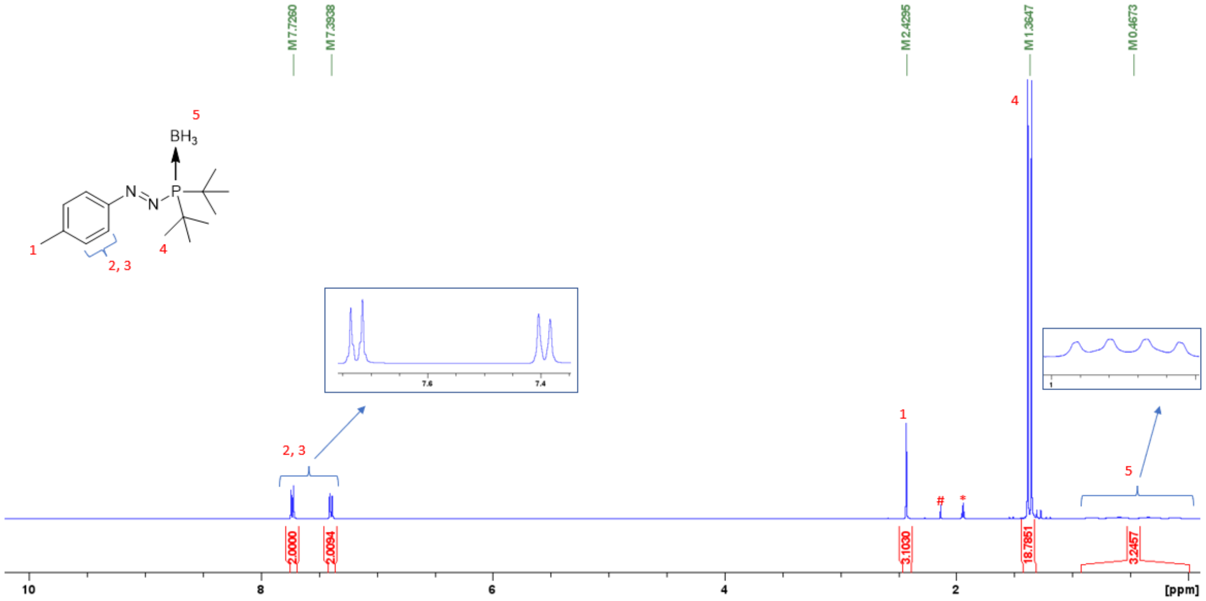

Figure S28. ^1H NMR spectrum of **2-Me** in CD_3CN. * = residual CHD_2CN; # = water in CD_3CN.

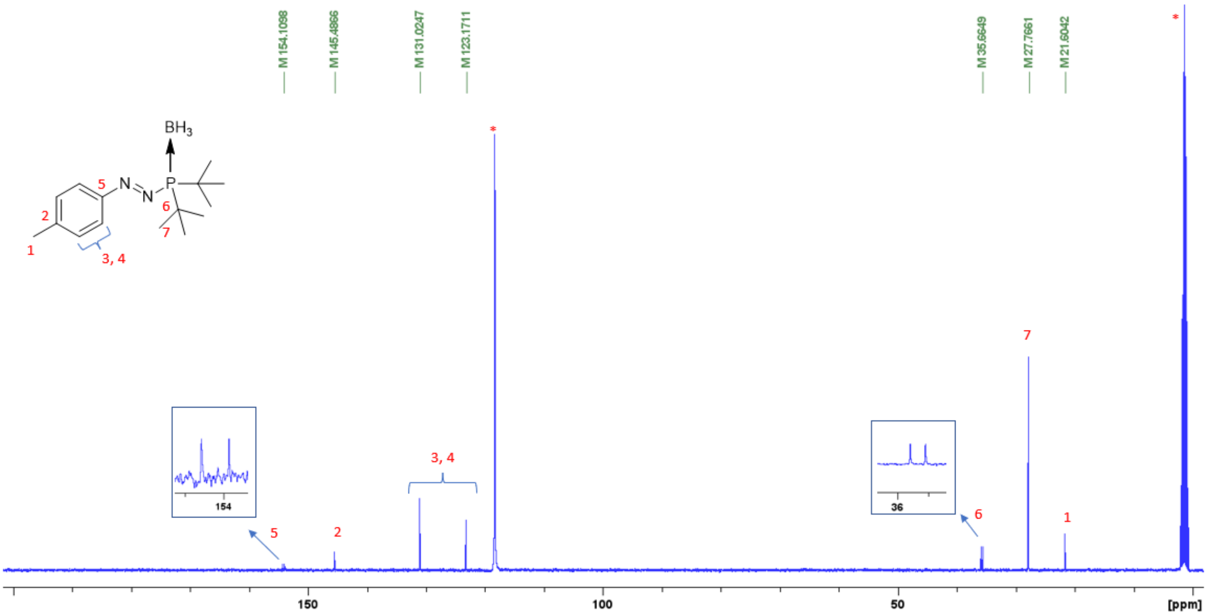

Figure S29. $^{13}C\{^{1}H\}$ spectrum of **2-Me** in CD_3CN. * = CD_3CN.

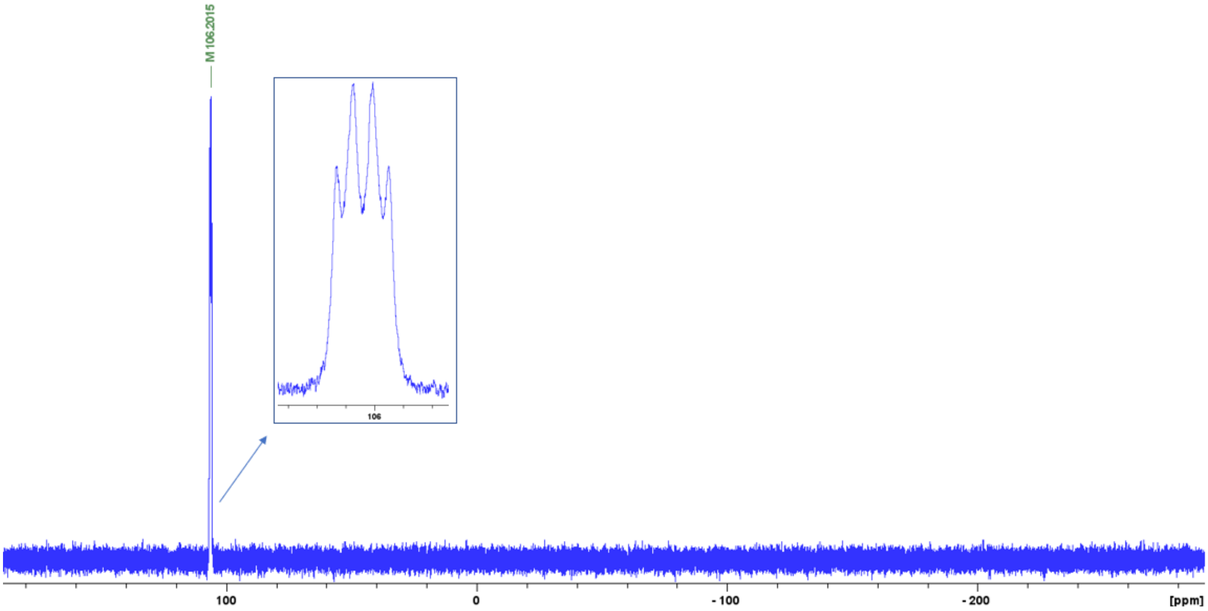

Figure S30. $^{31}P\{^{1}H\}$ spectrum of **2-Me** in CD_3CN.

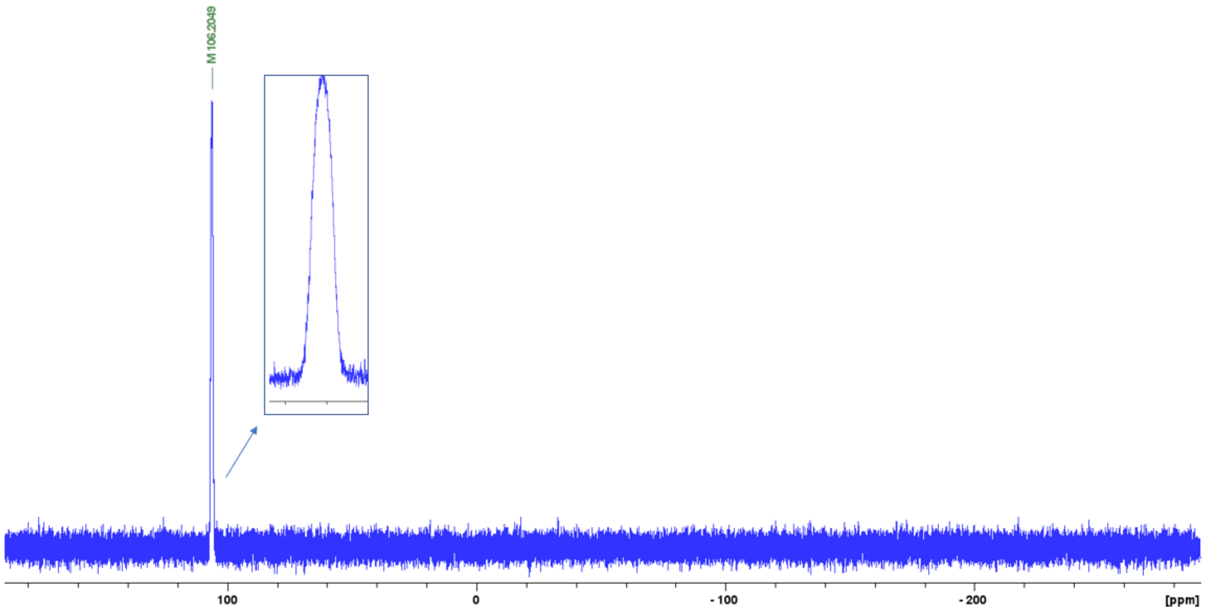

Figure S31. ^{31}P spectrum of **2-Me** in CD_3CN.

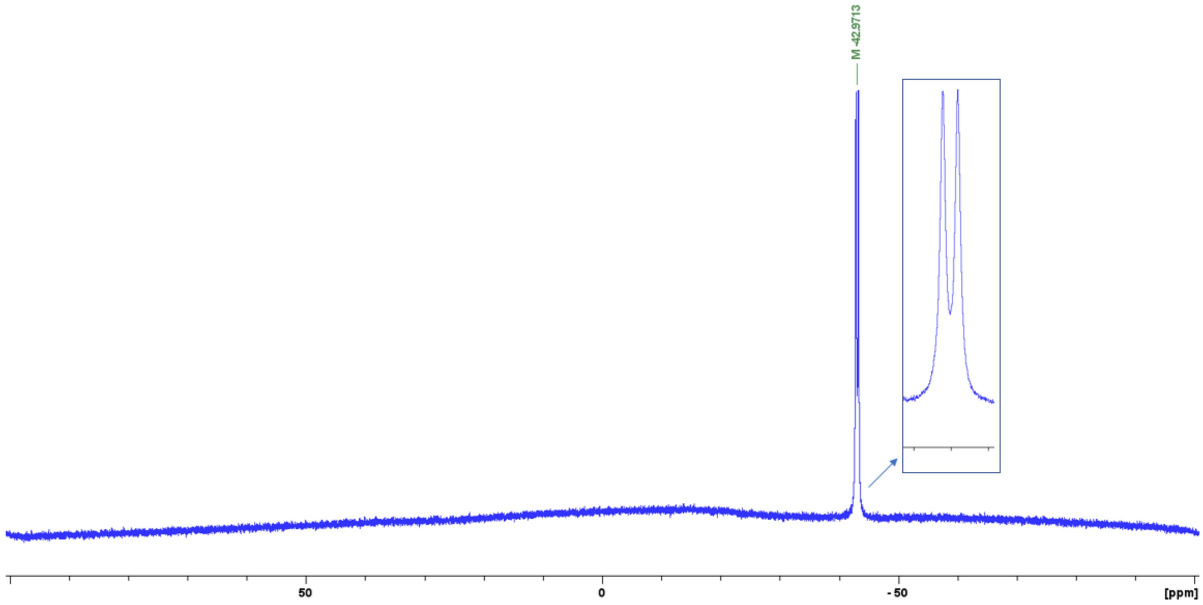

Figure S32. $^{11}B\{^1H\}$ spectrum of **2-Me** in CD_3CN.

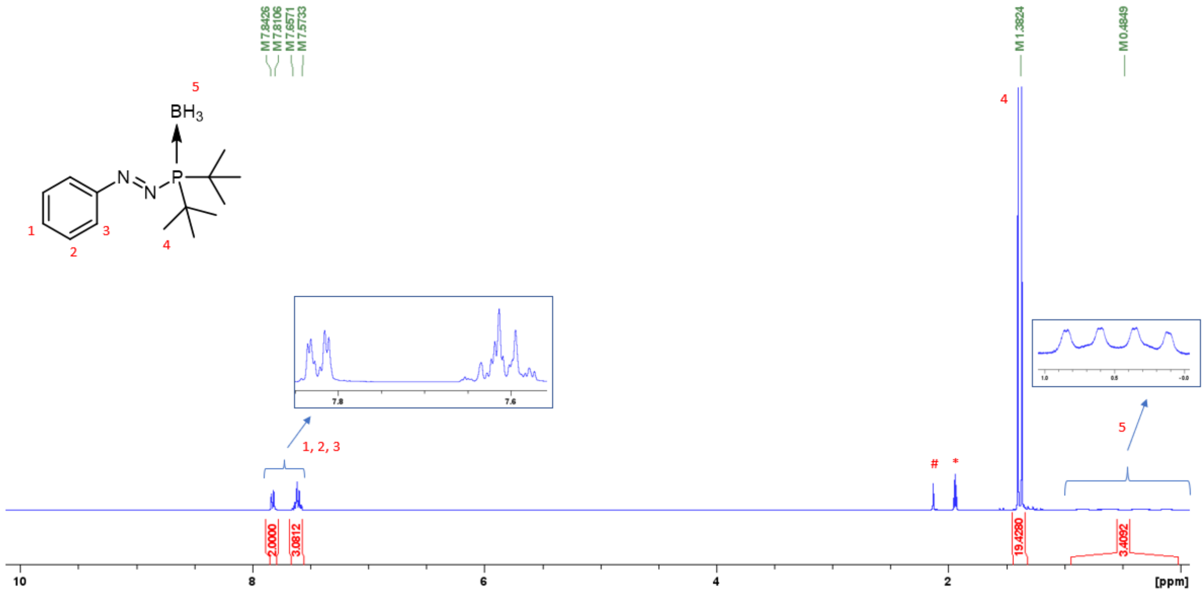

Figure S33. ^{1}H NMR spectrum of **2-H** in CD_3CN. * = residual CHD_2CN; # = water in CD_3CN.

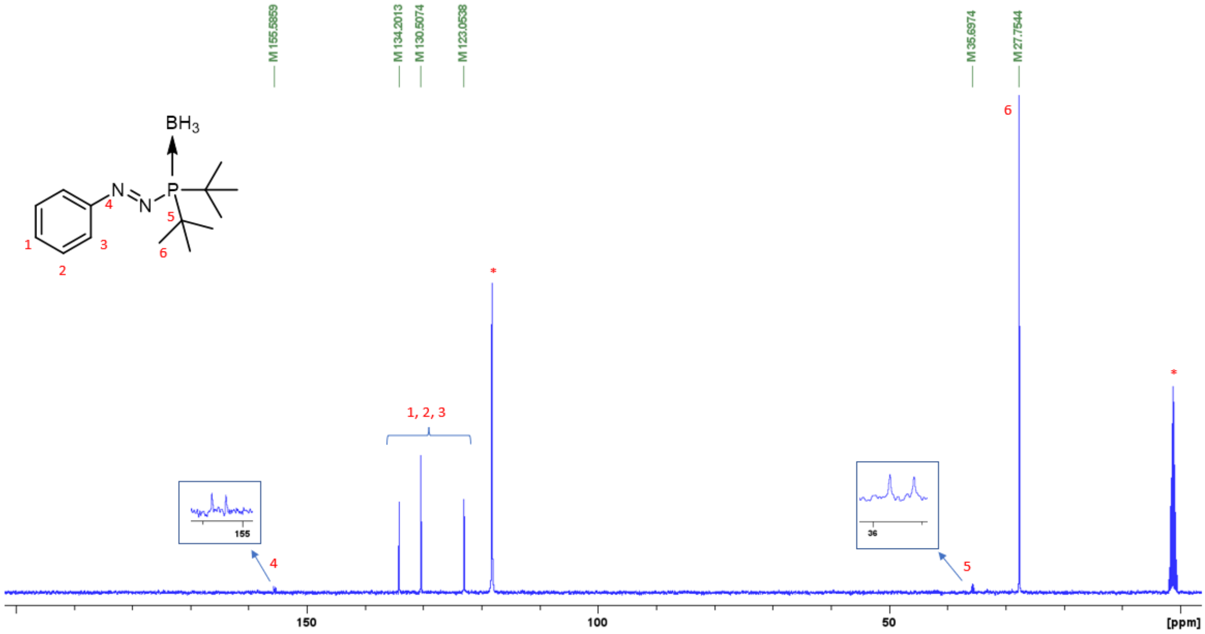

Figure S34. $^{13}C\{^{1}H\}$ spectrum of **2-H** in CD_3CN. * = CD_3CN.

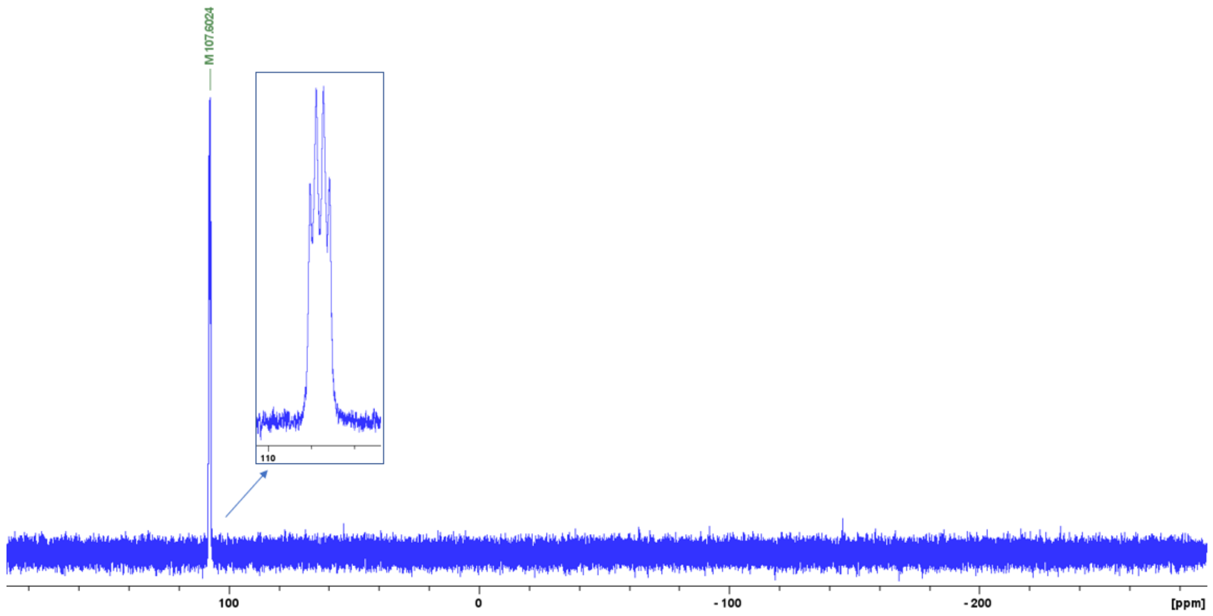

Figure S35. $^{31}P\{^{1}H\}$ spectrum of **2-H** in CD_3CN.

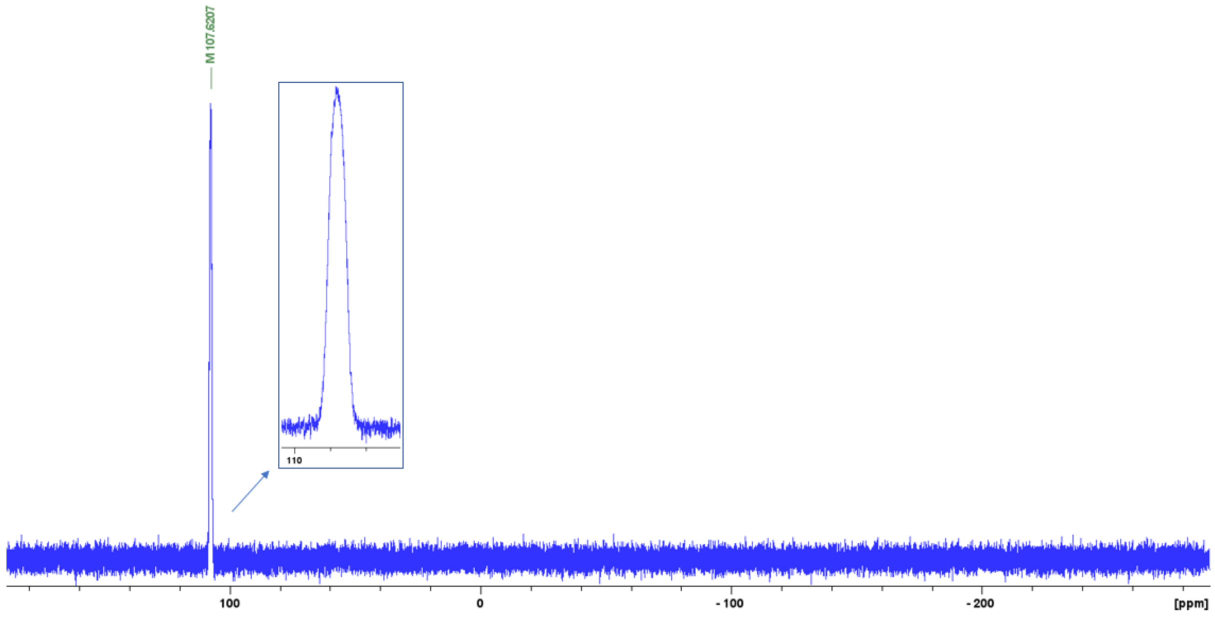

Figure S36. ^{31}P spectrum of **2-H** in CD_3CN.

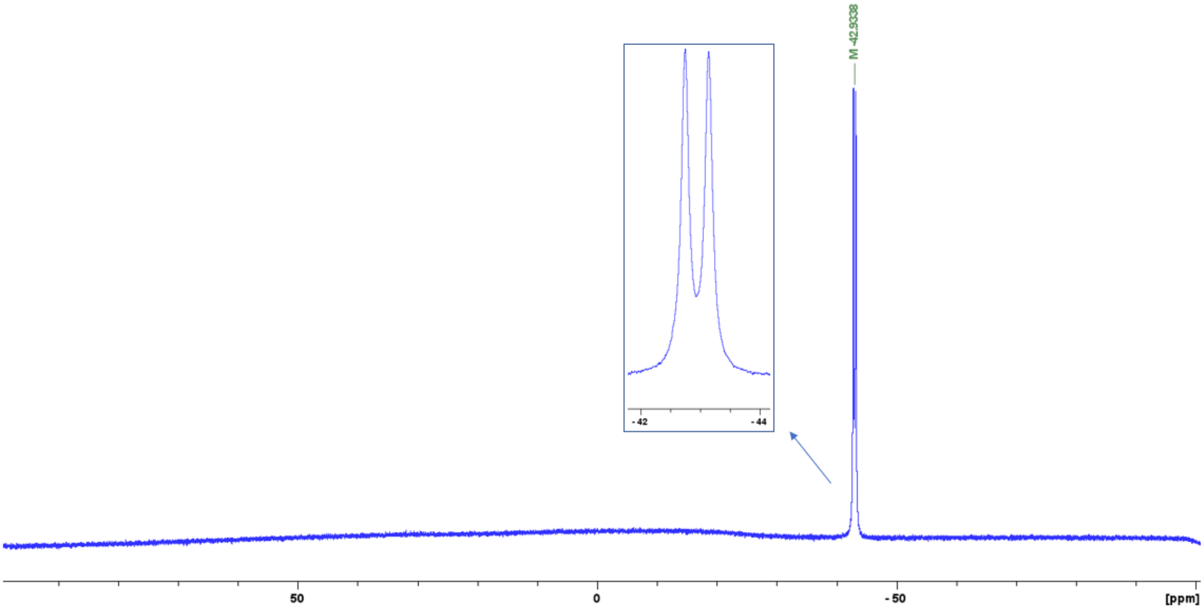

Figure S37. $^{11}B\{^{1}H\}$ spectrum of **2-H** in CD_3CN.

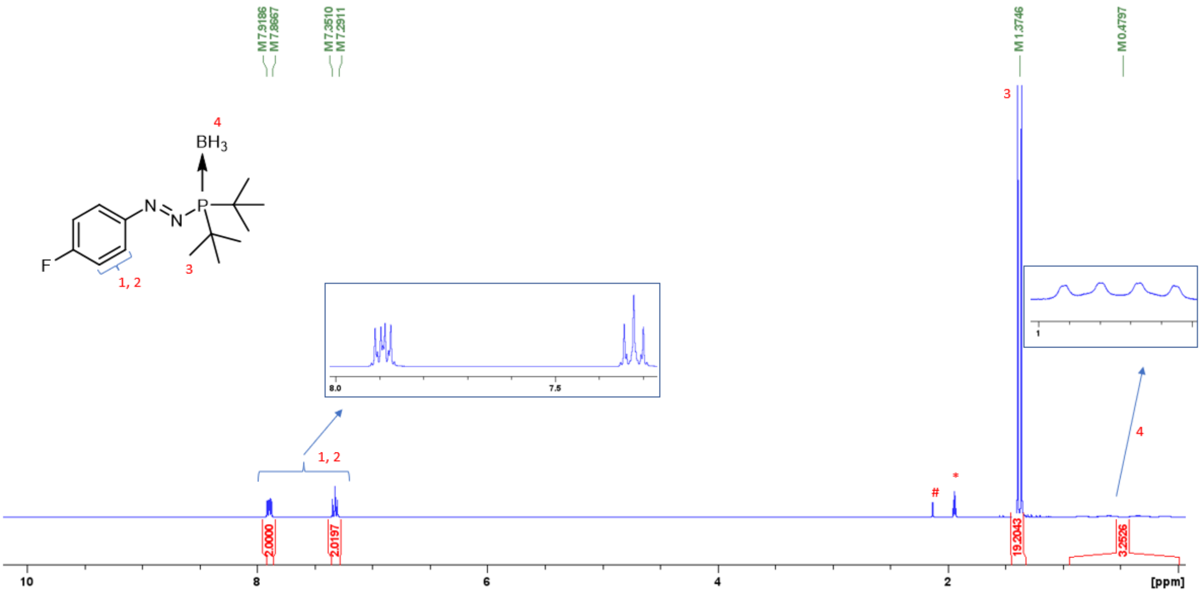

Figure S38. ^{1}H NMR spectrum of **2-F** in CD_3CN. * = residual CHD_2CN; # = water in CD_3CN.

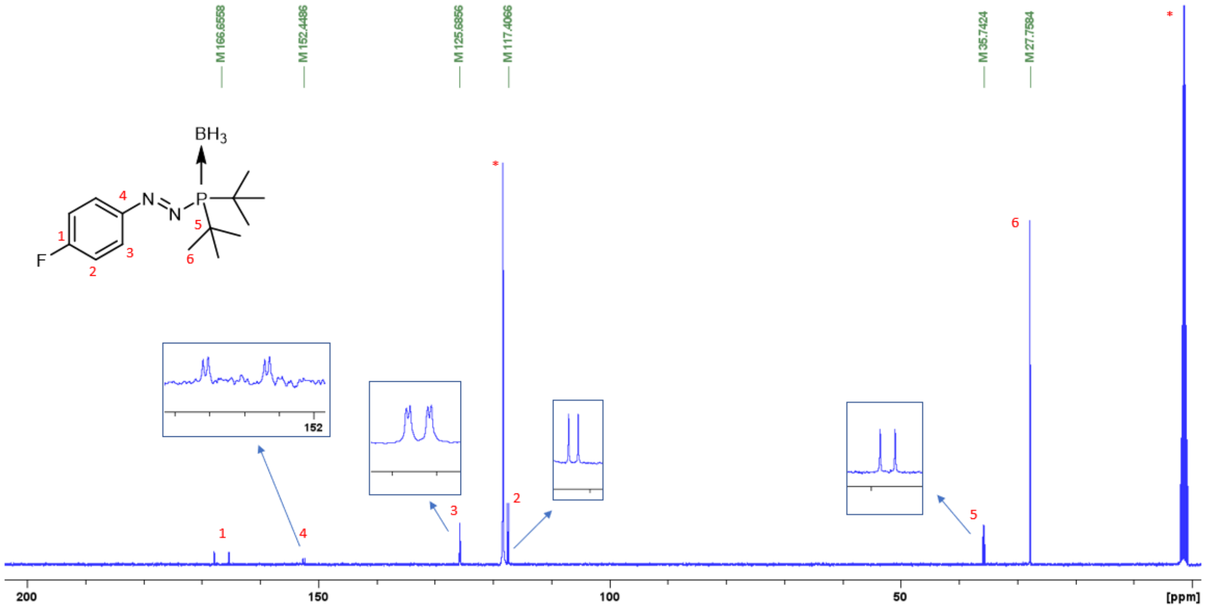

Figure S39. $^{13}C\{^{1}H\}$ spectrum of **2-F** in CD_3CN. * = CD_3CN.

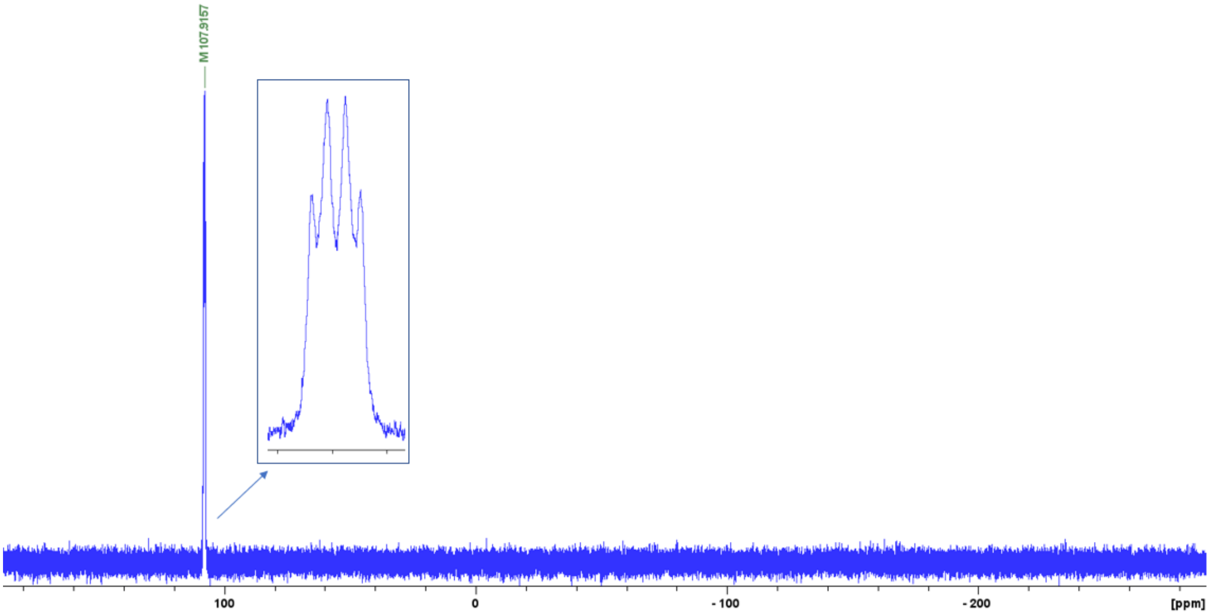

Figure S40. $^{31}P\{^{1}H\}$ spectrum of **2-F** in CD_3CN.

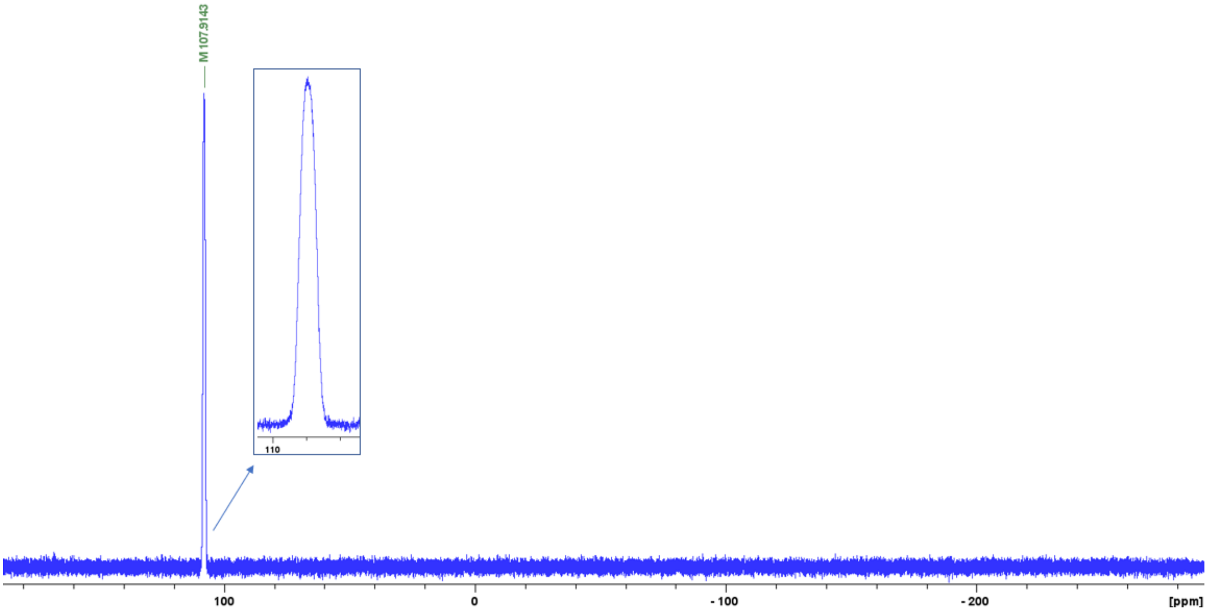

Figure S41. ^{31}P spectrum of **2-F** in CD_3CN.

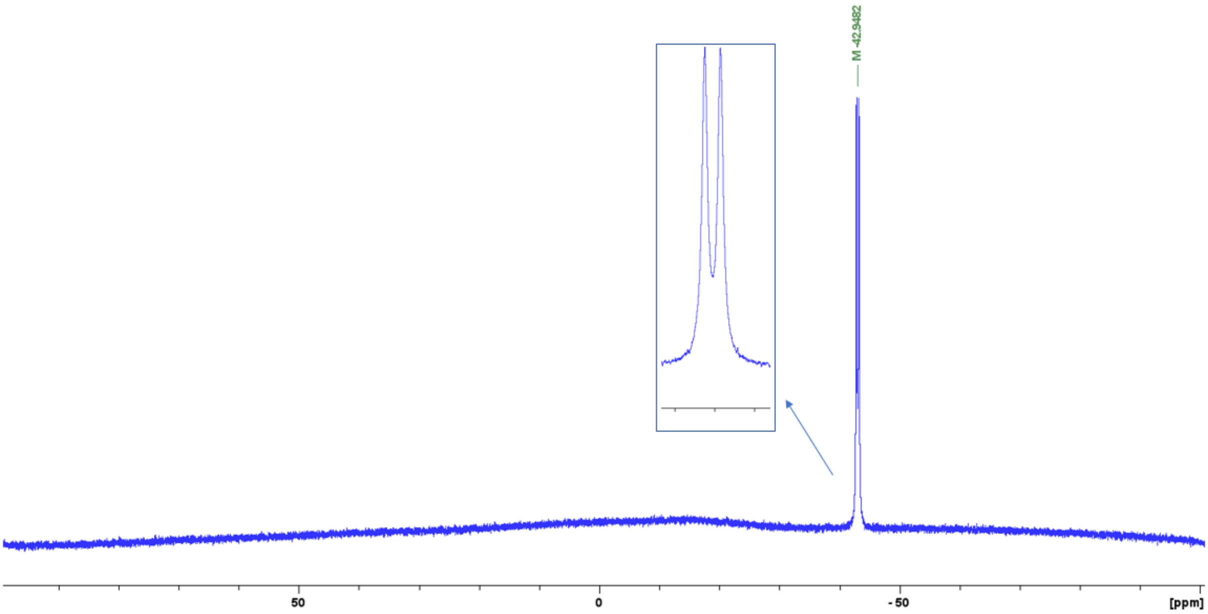

Figure S42. $^{11}B\{^1H\}$ spectrum of **2-F** in CD_3CN.

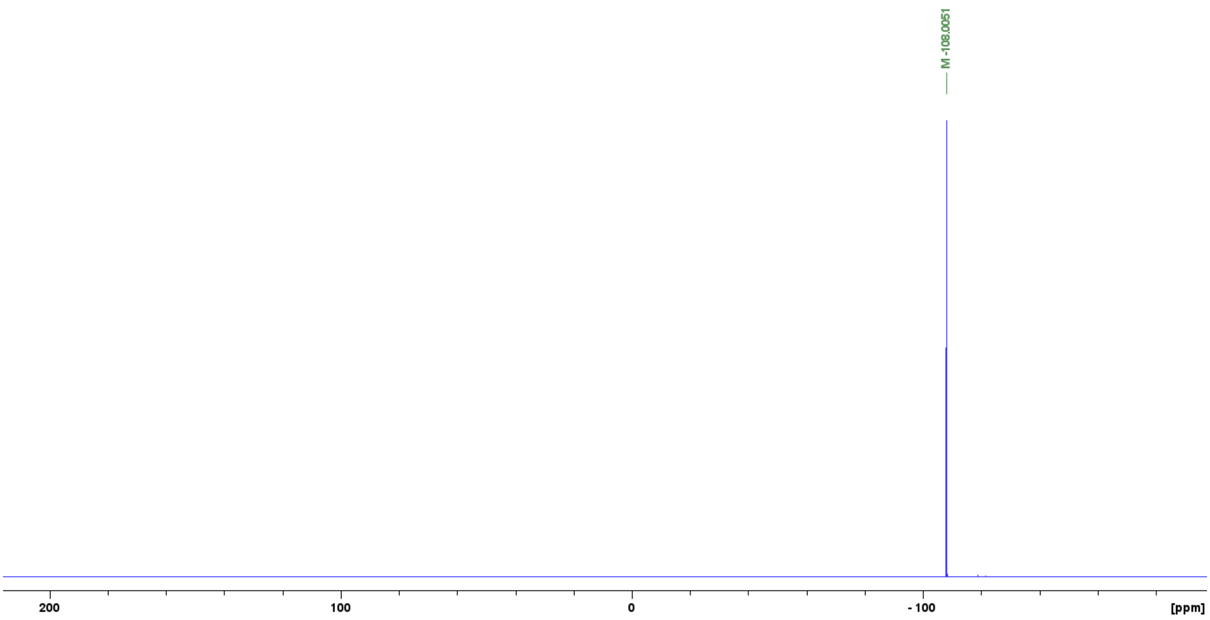

Figure S43. $^{19}F\{^{1}H\}$ spectrum of **2-F** in CD_3CN.

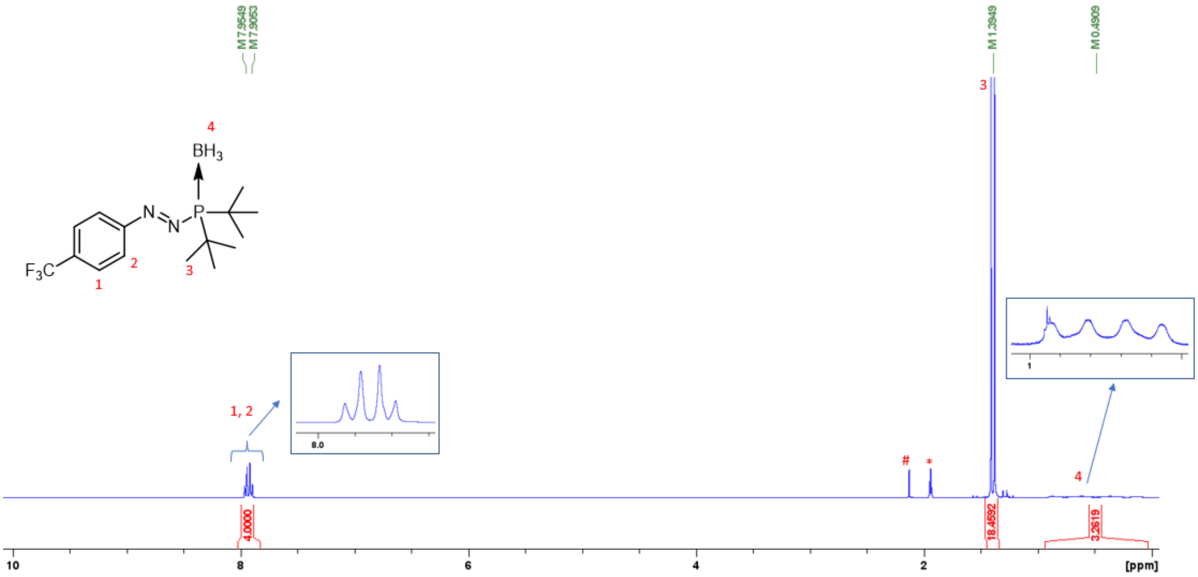

Figure S44. ^{1}H NMR spectrum of **2-CF$_3$** in CD_3CN. * = residual CHD_2CN; # = water in CD_3CN.

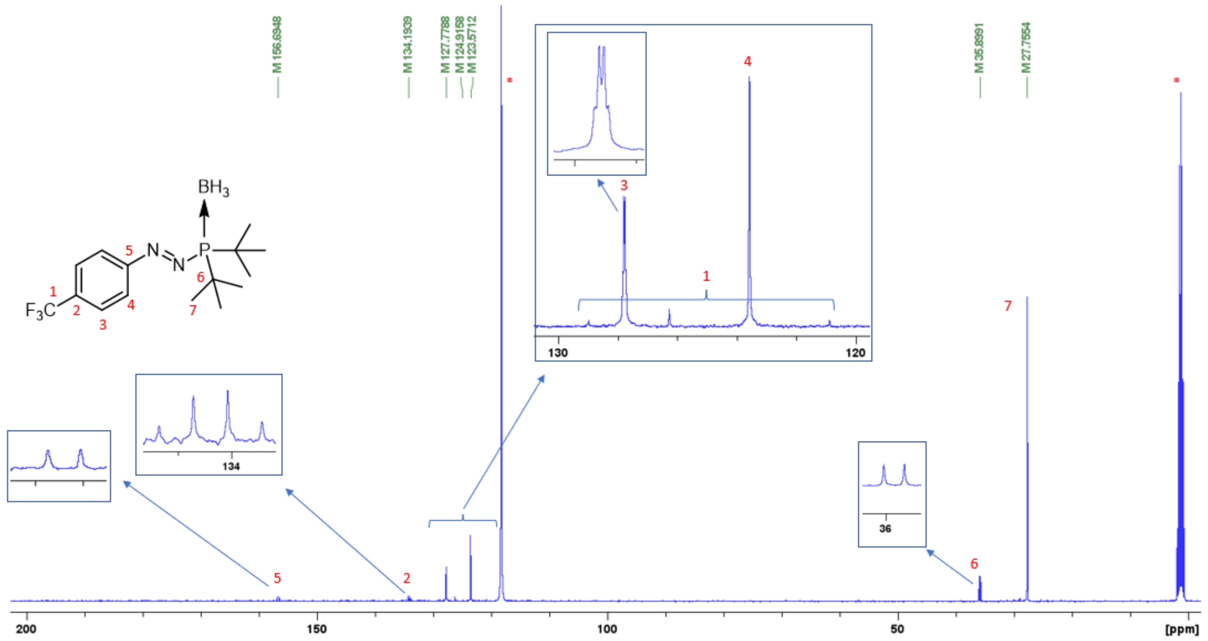

Figure S45. $^{13}C\{^1H\}$ spectrum of **2-CF$_3$** in CD_3CN. * = CD_3CN.

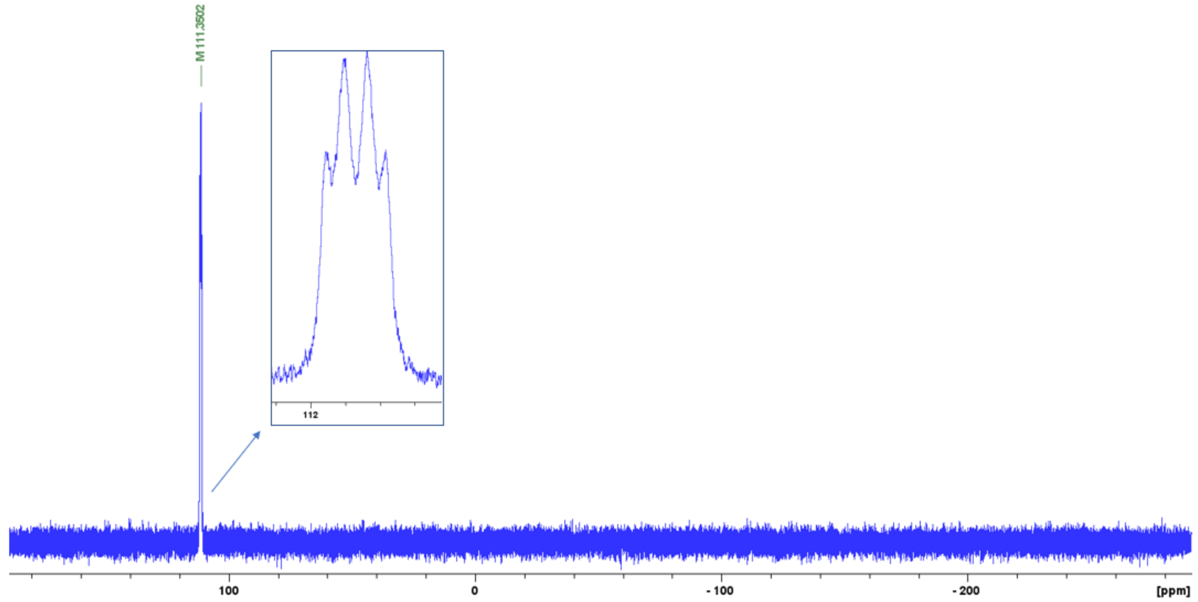

Figure S46. $^{31}P\{^1H\}$ spectrum of **2-CF$_3$** in CD_3CN.

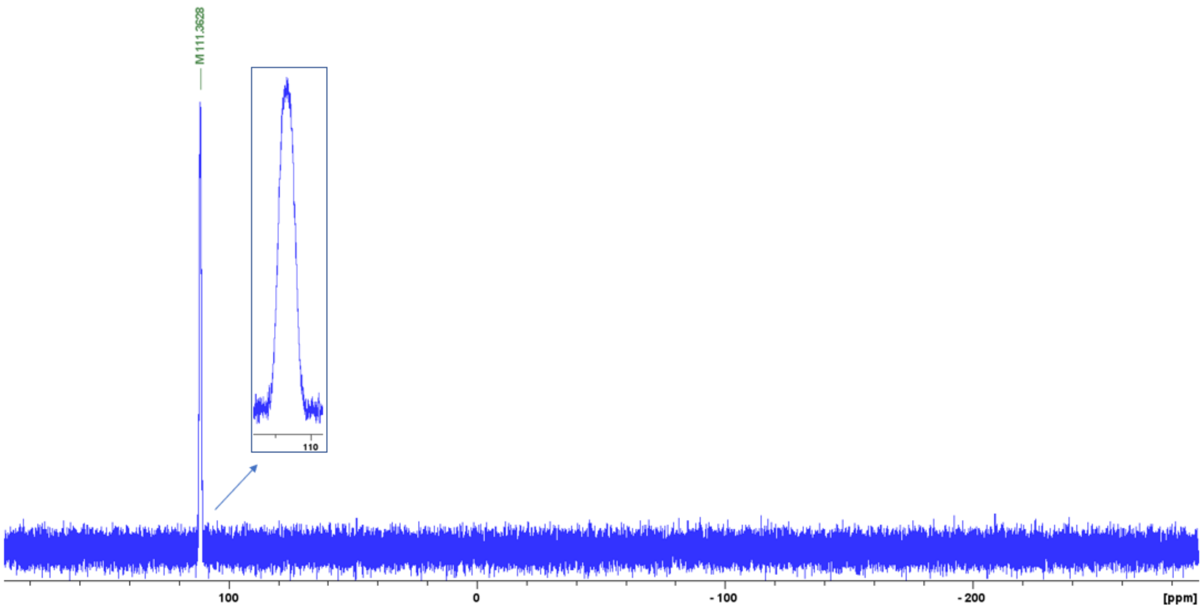

Figure S47. ^{31}P spectrum of **2-CF$_3$** in CD_3CN.

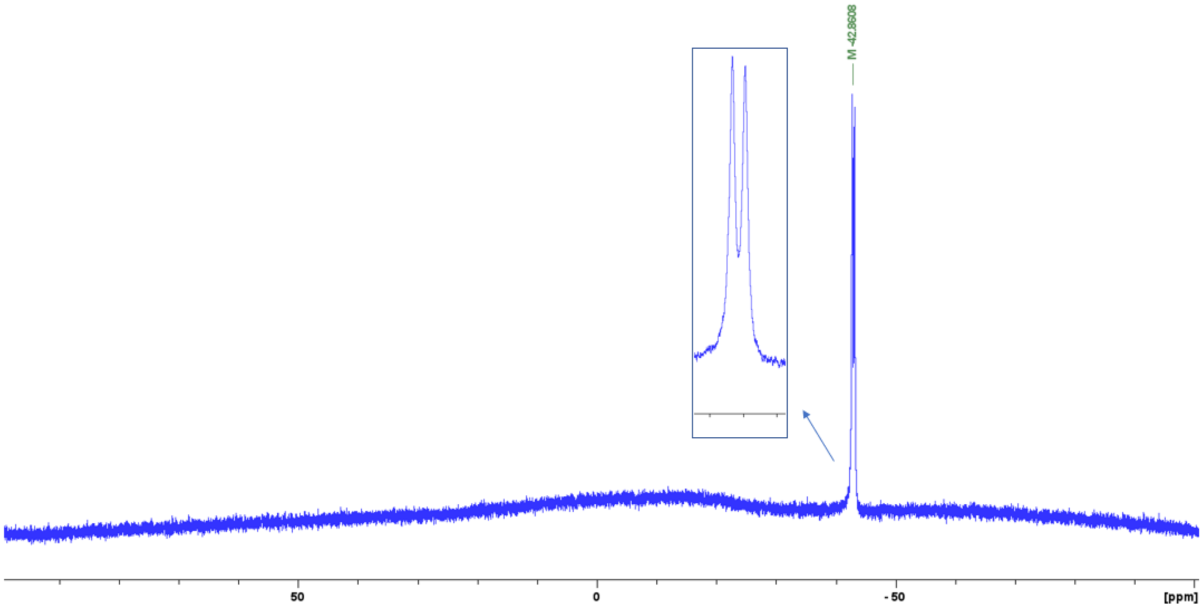

Figure S48. $^{11}B\{^1H\}$ spectrum of **2-CF$_3$** in CD_3CN.

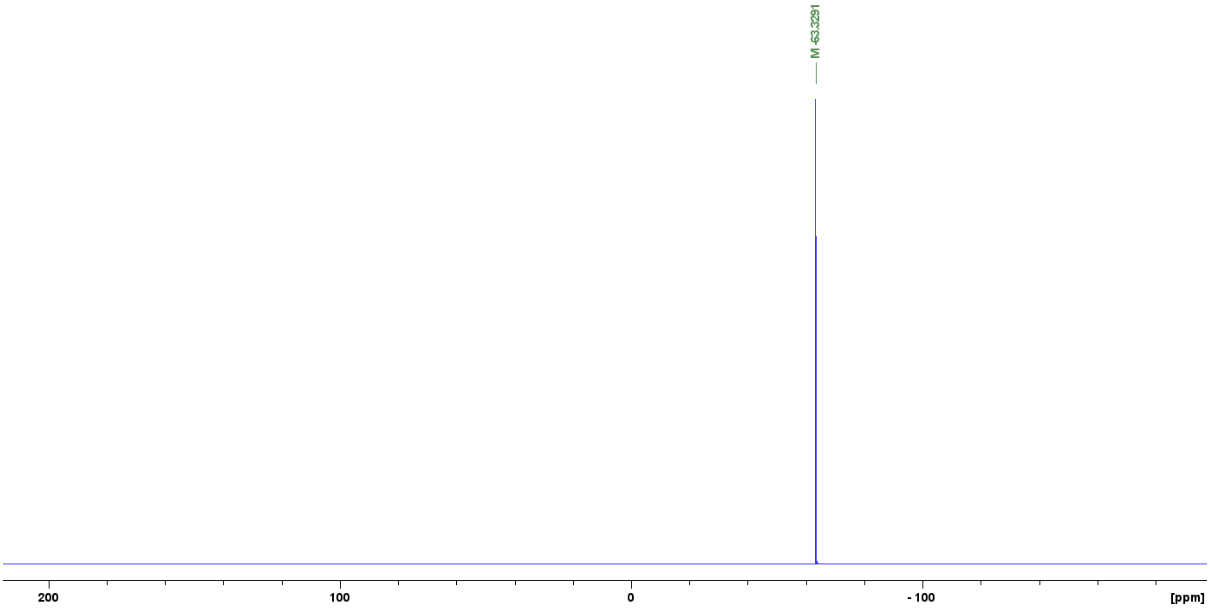

Figure S49. $^{19}F\{^{1}H\}$ spectrum of **2-CF$_3$** in CD_3CN.

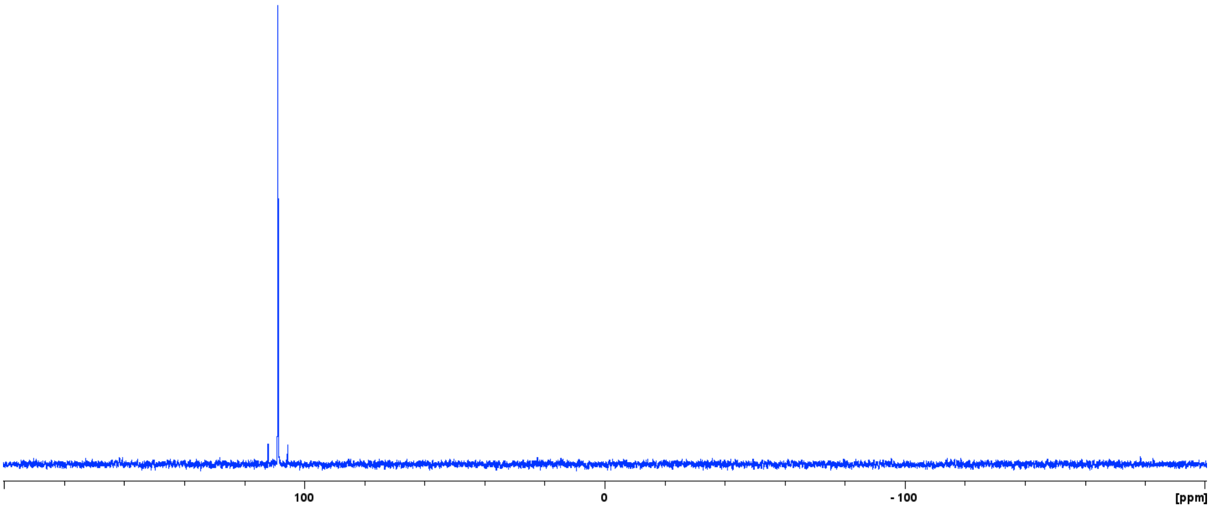

Figure S50. Crude $^{31}P\{^{1}H\}$ NMR spectrum of **3-NMe$_2$** in toluene. $^{1}J_{P-Se}$ = 794 Hz.

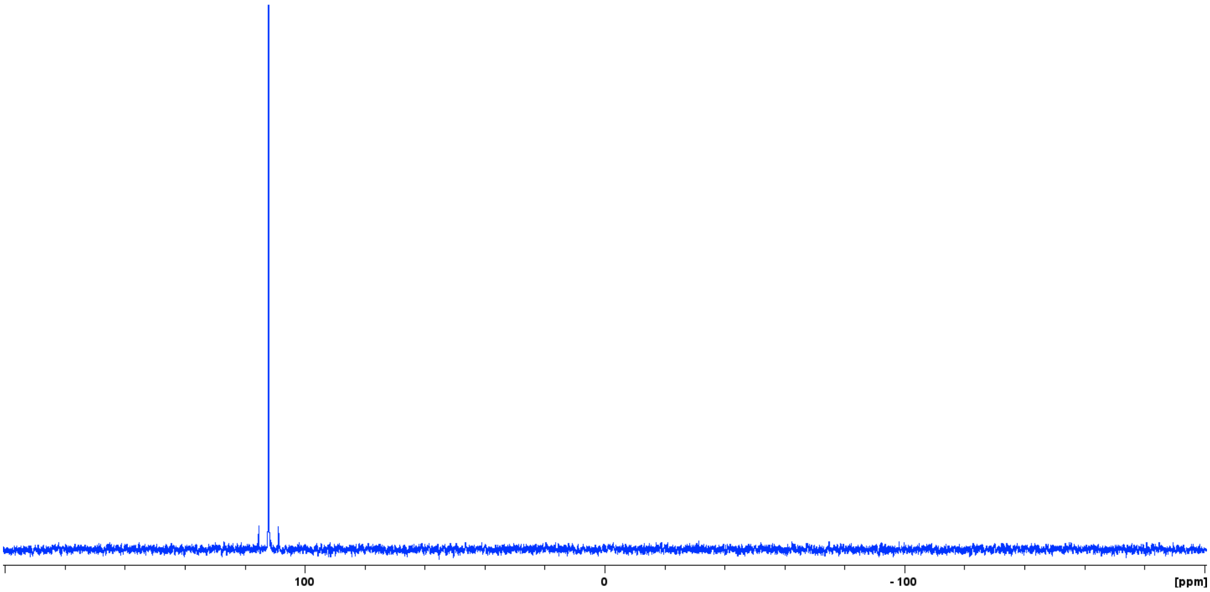

Figure S51. $^{31}P\{^1H\}$ NMR spectrum of **3-OMe** in toluene. $^1J_{P\text{–}Se}$ = 801 Hz.

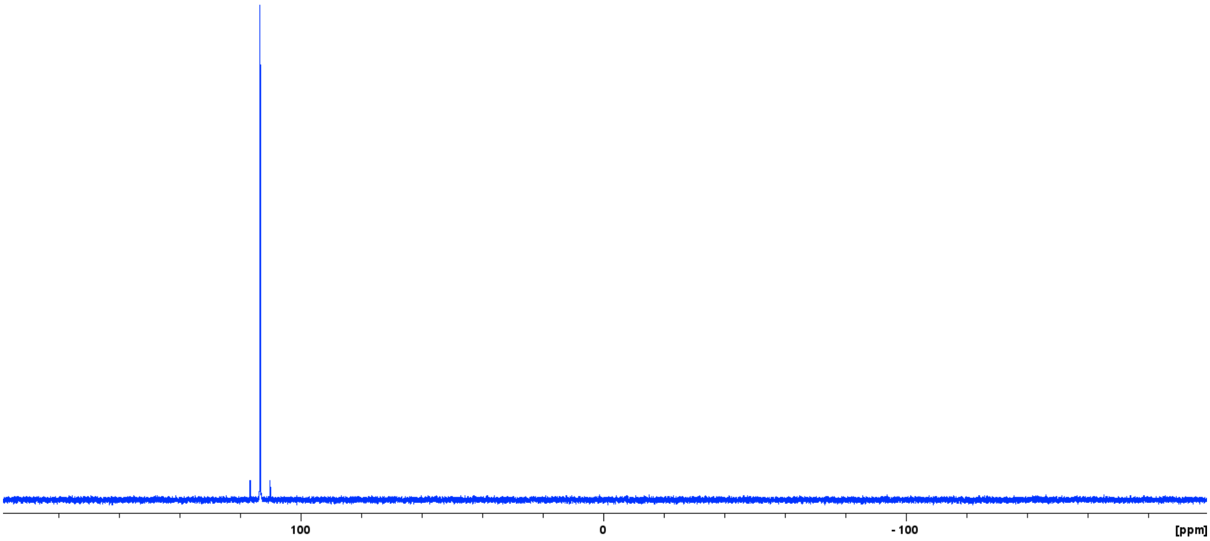

Figure S52. $^{31}P\{^1H\}$ NMR spectrum of **3-Me** in toluene. $^1J_{P\text{–}Se}$ = 804 Hz.

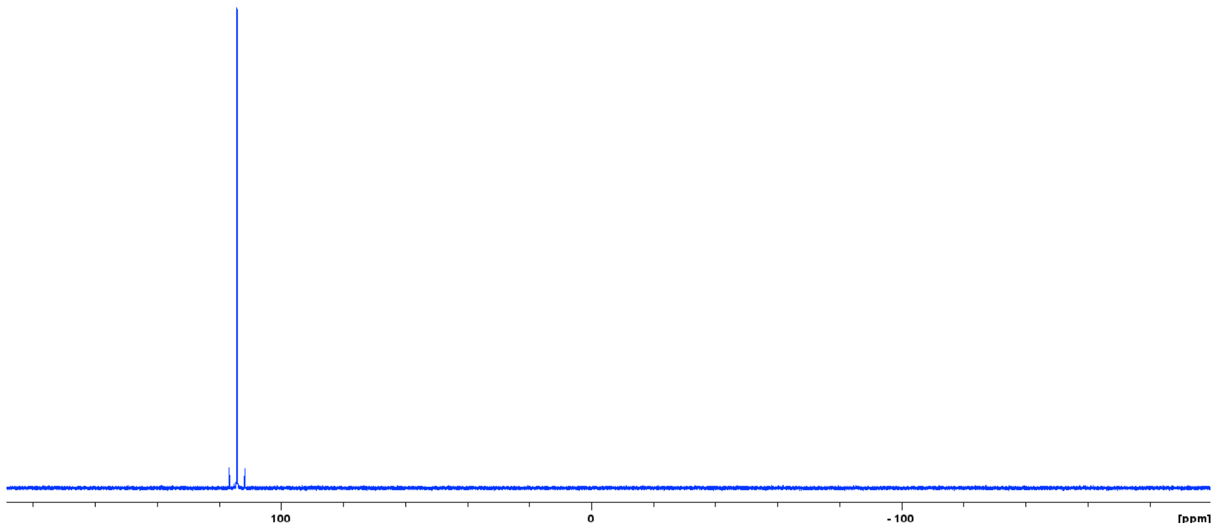

Figure S53. $^{31}P\{^1H\}$ NMR spectrum of **3-H** in toluene. $^1J_{P-Se}$ = 806 Hz.

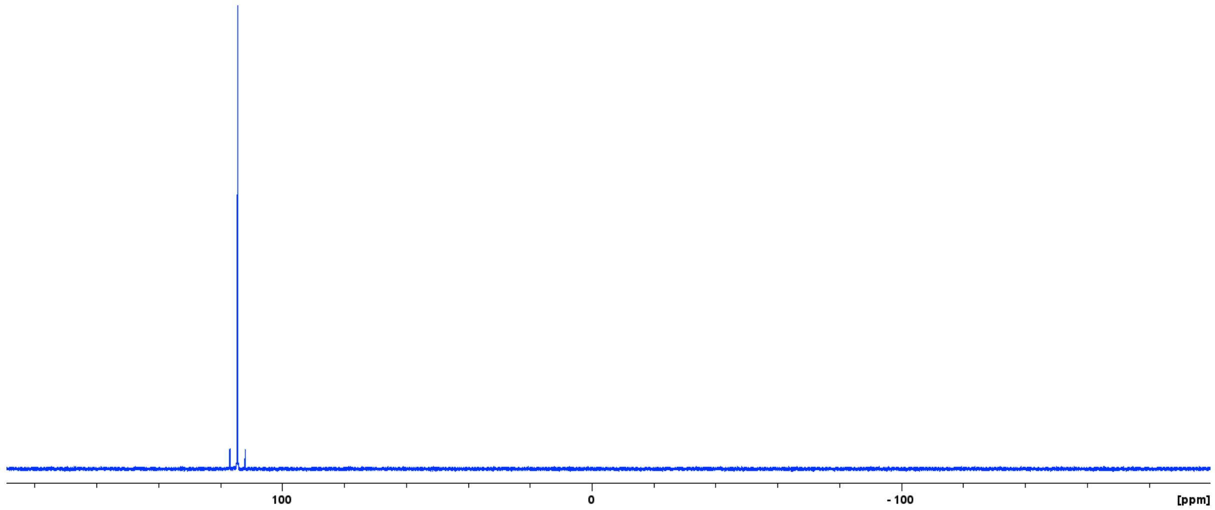

Figure S54. $^{31}P\{^1H\}$ NMR spectrum of **3-F** in toluene. $^1J_{P-Se}$ = 806 Hz.

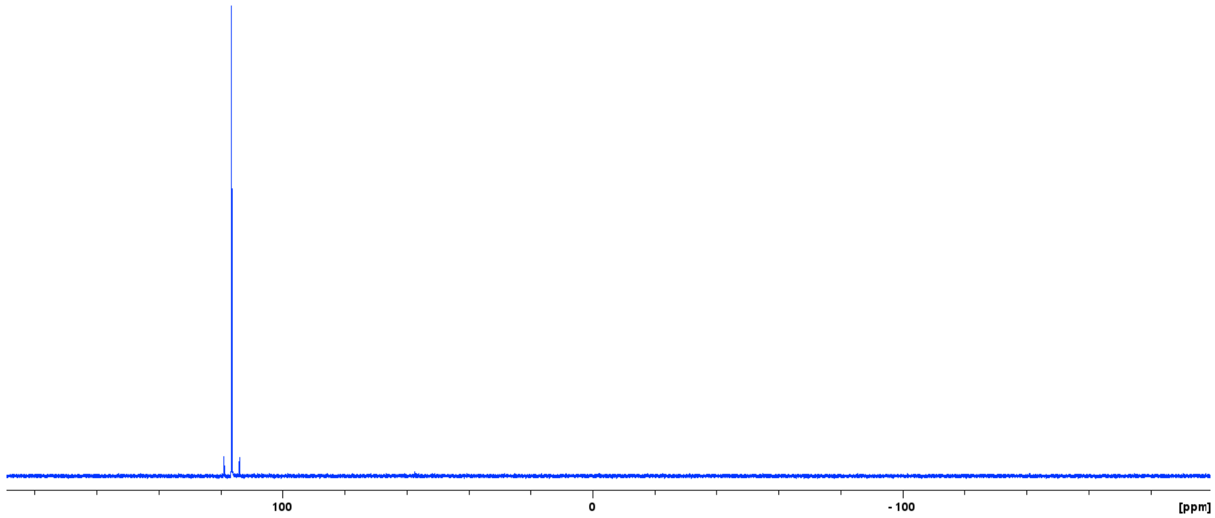

Figure S55. $^{31}P\{^1H\}$ NMR spectrum of **3-CF$_3$** in toluene. $^1J_{P-Se}$ = 810 Hz.

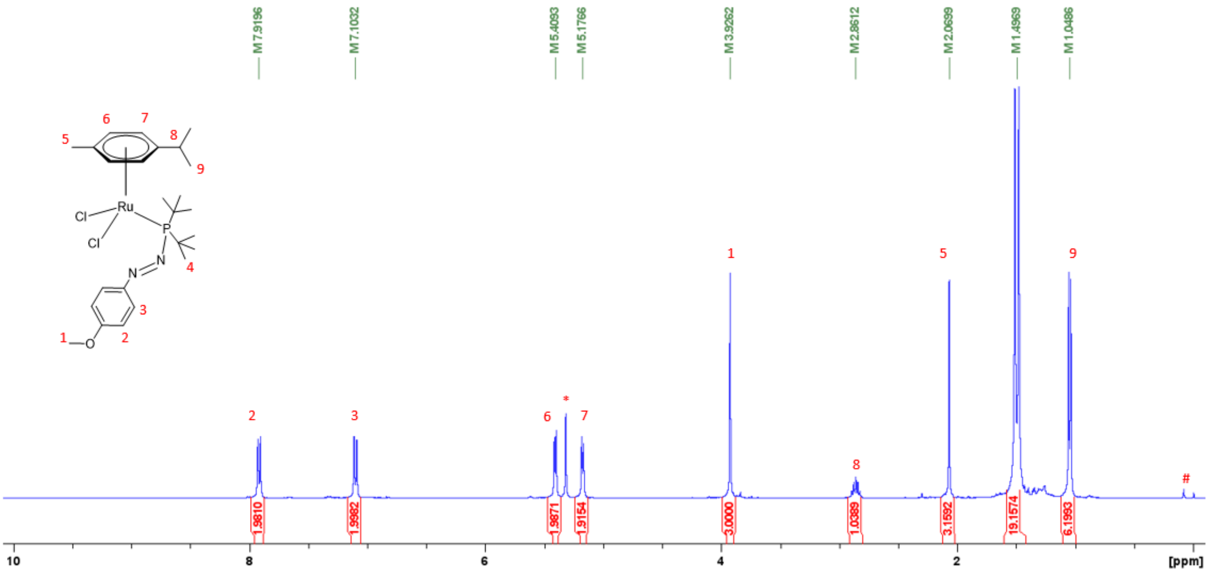

Figure S56. ^{1}H NMR spectrum of **4-OMe** in CD_2Cl_2. * = residual $CDHCl_2$, # = silicone grease and TMS internal reference.

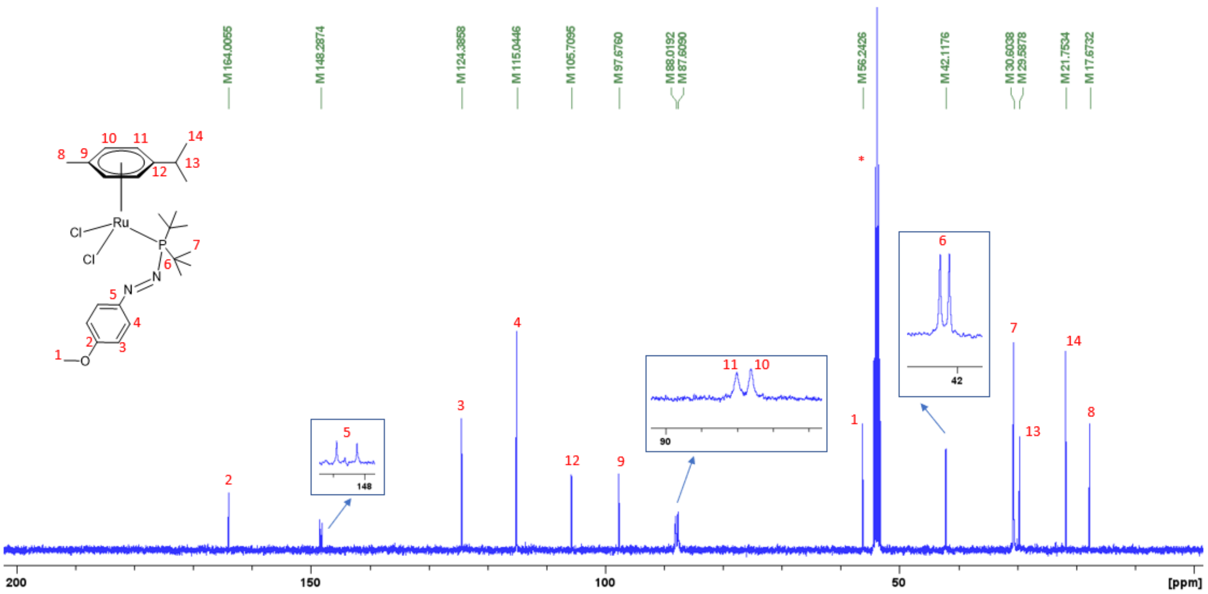

Figure S57. $^{13}C\{^{1}H\}$ NMR spectrum of **4-OMe** in CD_2Cl_2. * = CD_2Cl_2.

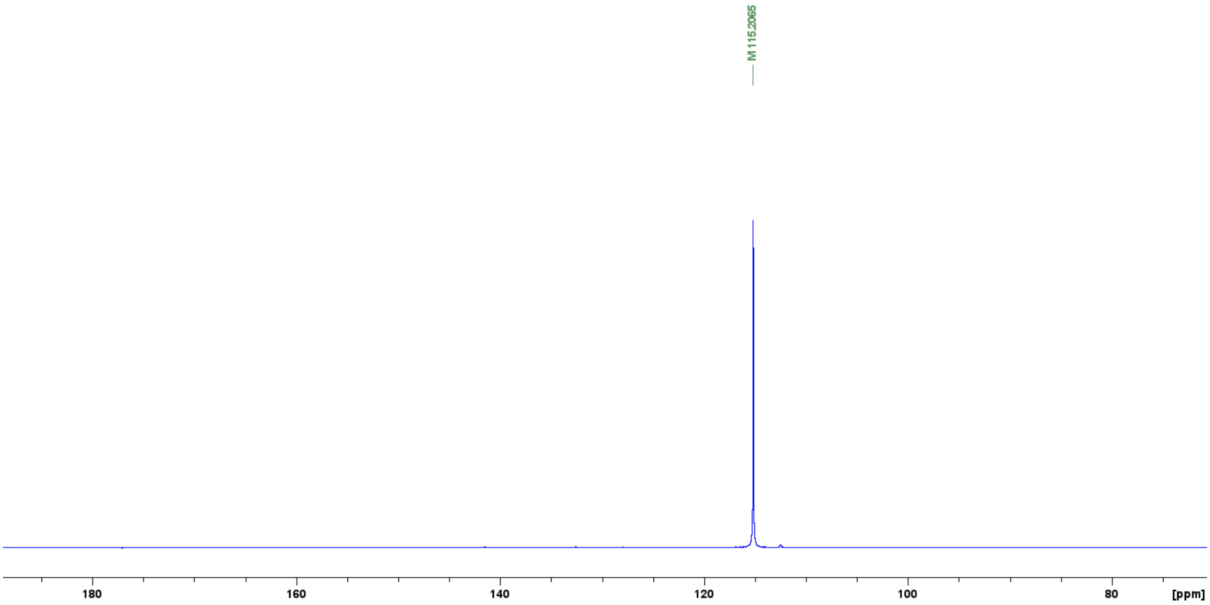

Figure S58. $^{31}P\{^{1}H\}$ NMR spectrum of **4-OMe** in CD_2Cl_2.

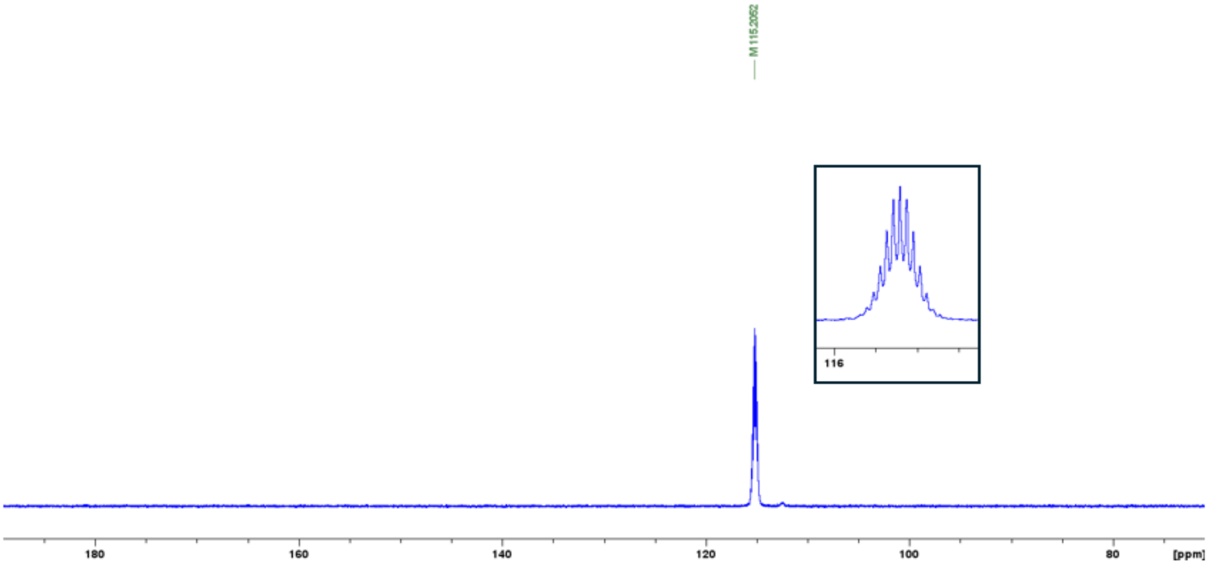

Figure S59. ^{31}P NMR spectrum of **4-OMe** in CD_2Cl_2.

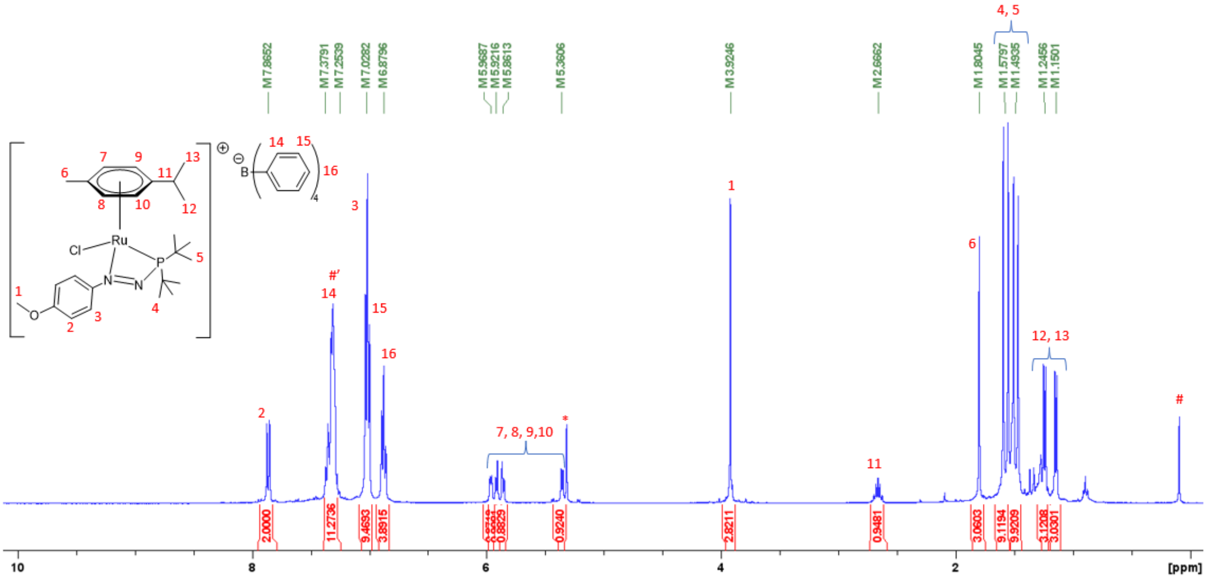

Figure S60. ^{1}H NMR spectrum of **5-OMe** in CD2Cl2. * = residual $CDHCl_2$, # = silicone grease, #' = residual chlorobenzene.

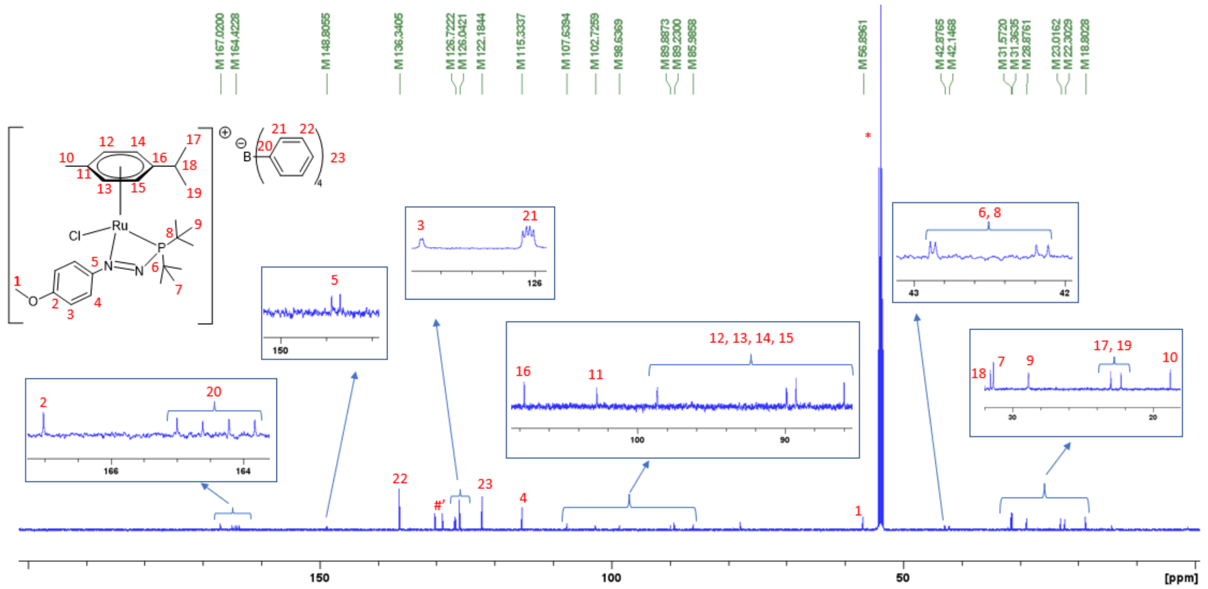

Figure S61. $^{13}C\{^{1}H\}$ NMR spectrum of **5-OMe** in CD_2Cl_2. * = CD_2Cl_2, #' = residual chlorobenzene.

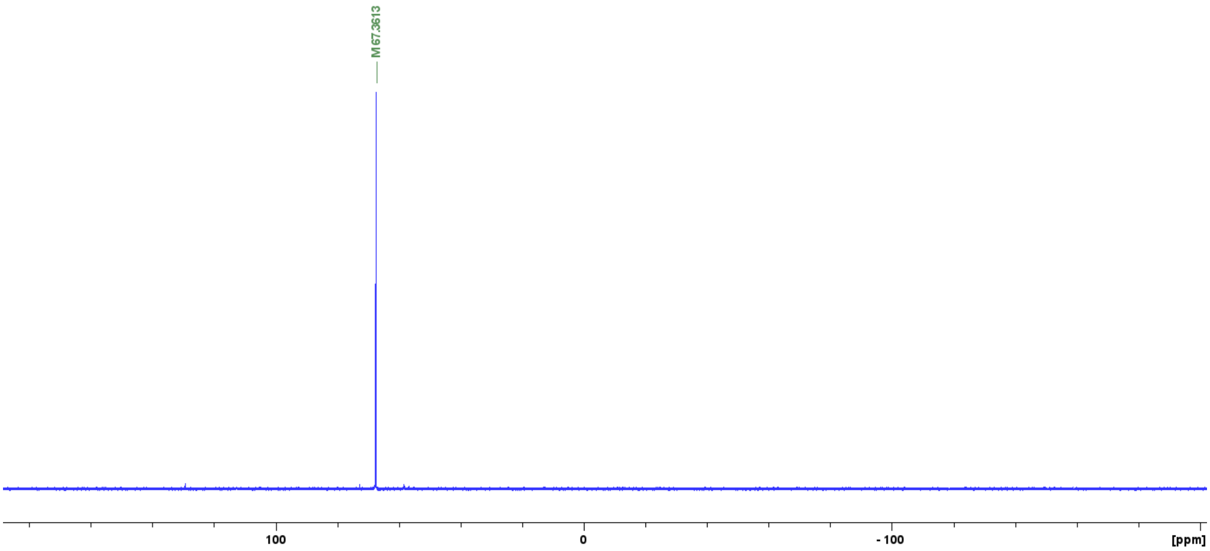

Figure S62. $^{31}P\{^{1}H\}$ NMR spectrum of **5-OMe** in CD_2Cl_2.

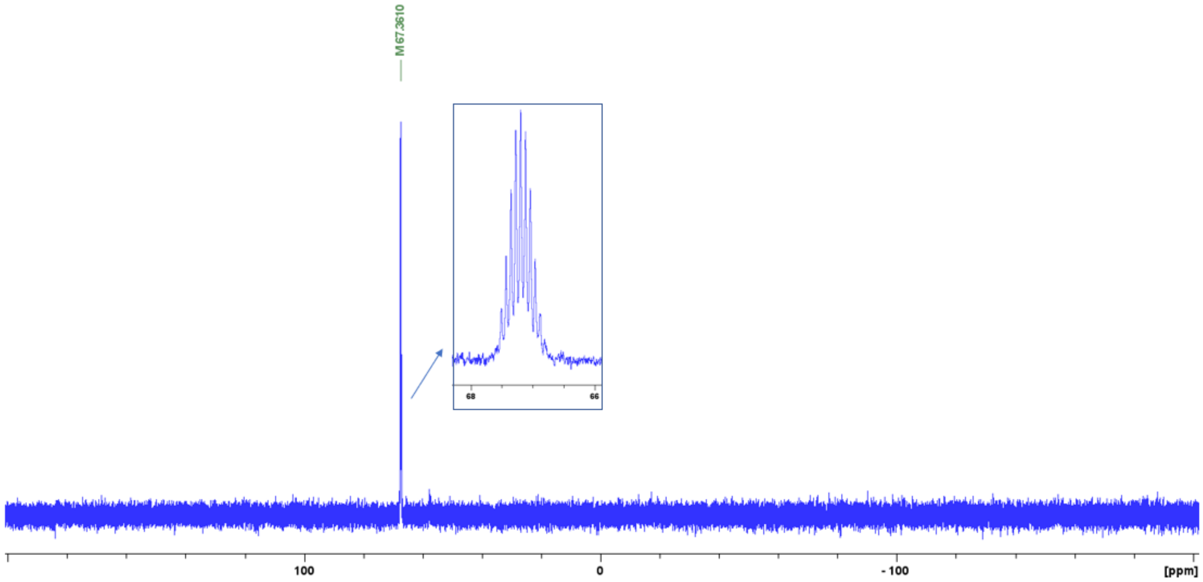

Figure S63. ^{31}P NMR spectrum of **5-OMe** in CD_2Cl_2.

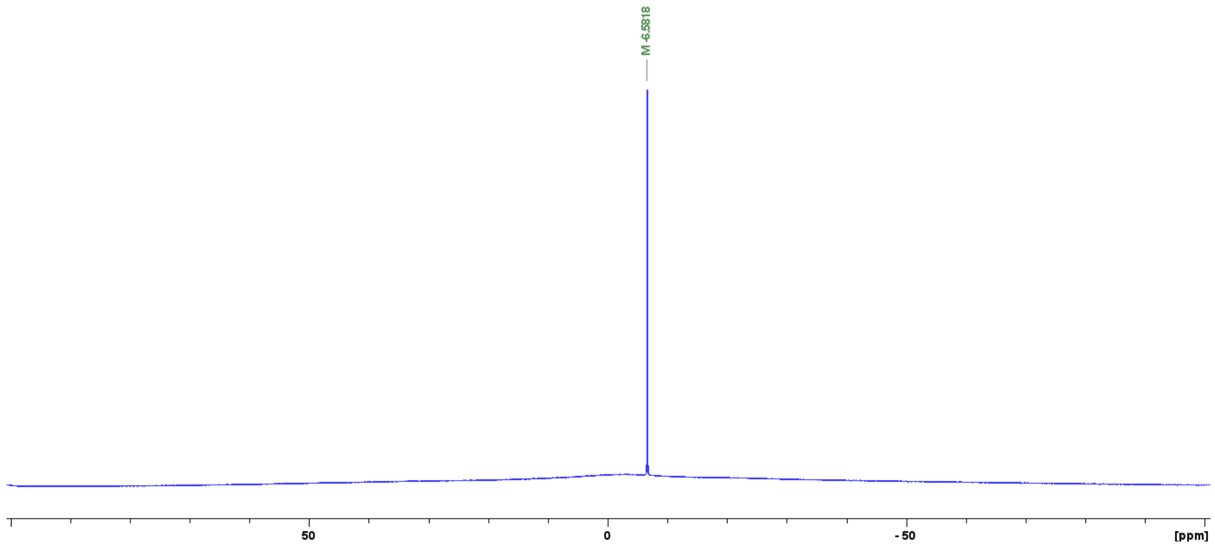

Figure S64. $^{11}B\{^{1}H\}$ NMR spectrum of **5-OMe** in CD_2Cl_2.

S2.2. UV/Vis Spectra

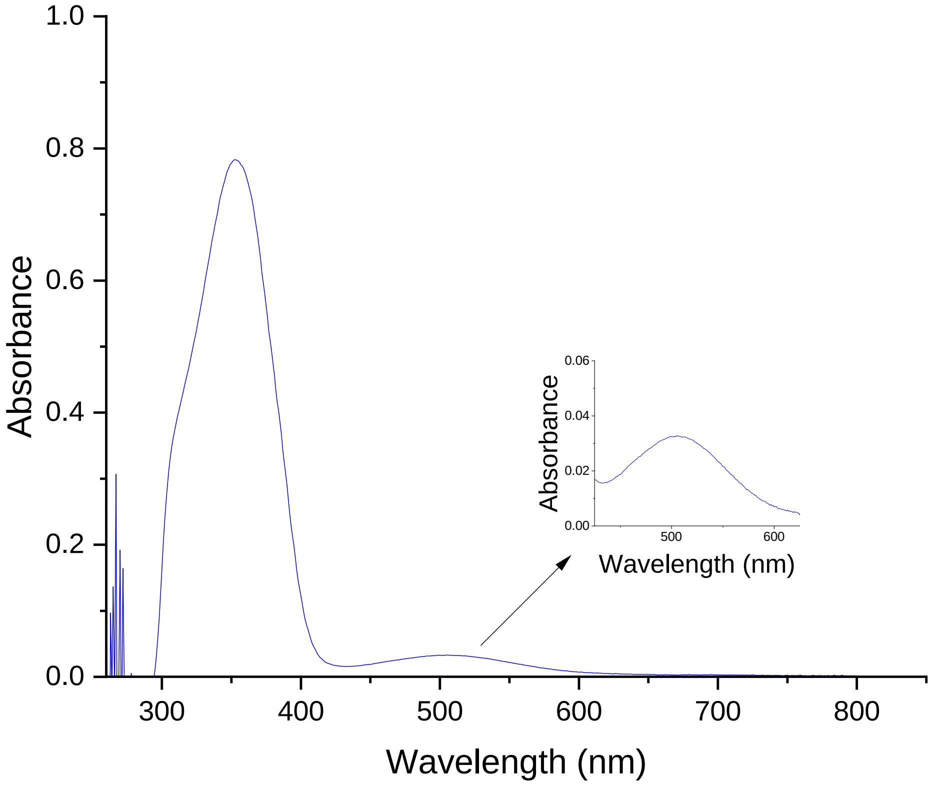

Figure S65. UV/Vis spectrum of **1-OMe** (5×10^{-5} M in toluene)

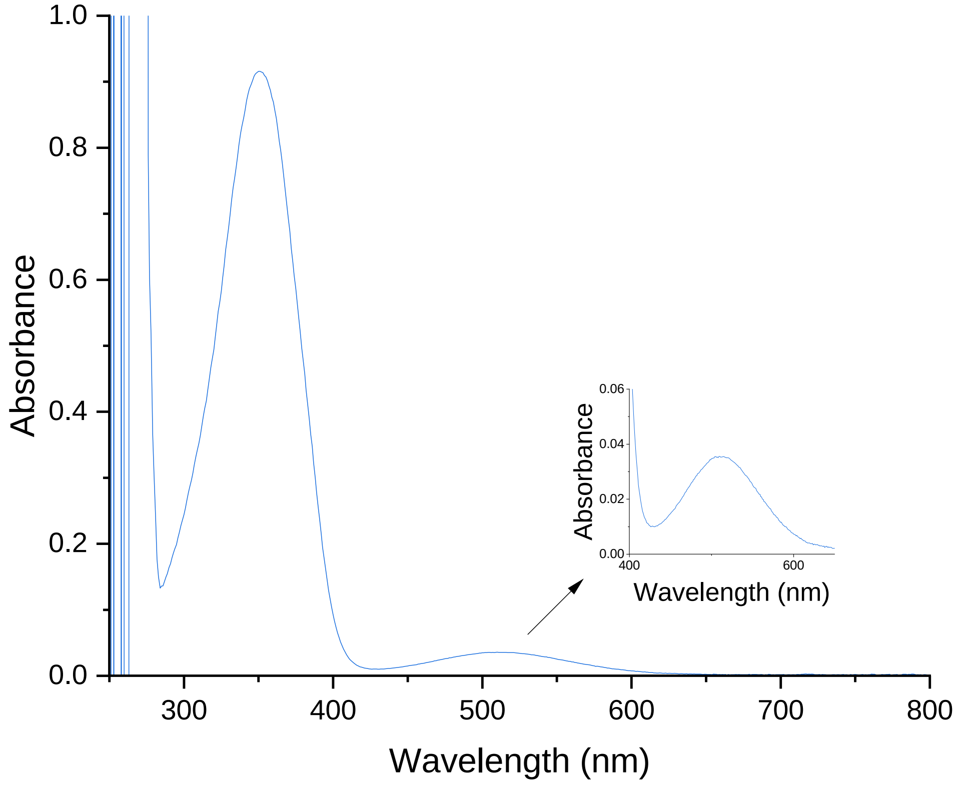

Figure S66. UV/Vis spectrum of **1-Me** (5 x 10^{-5} M in toluene)

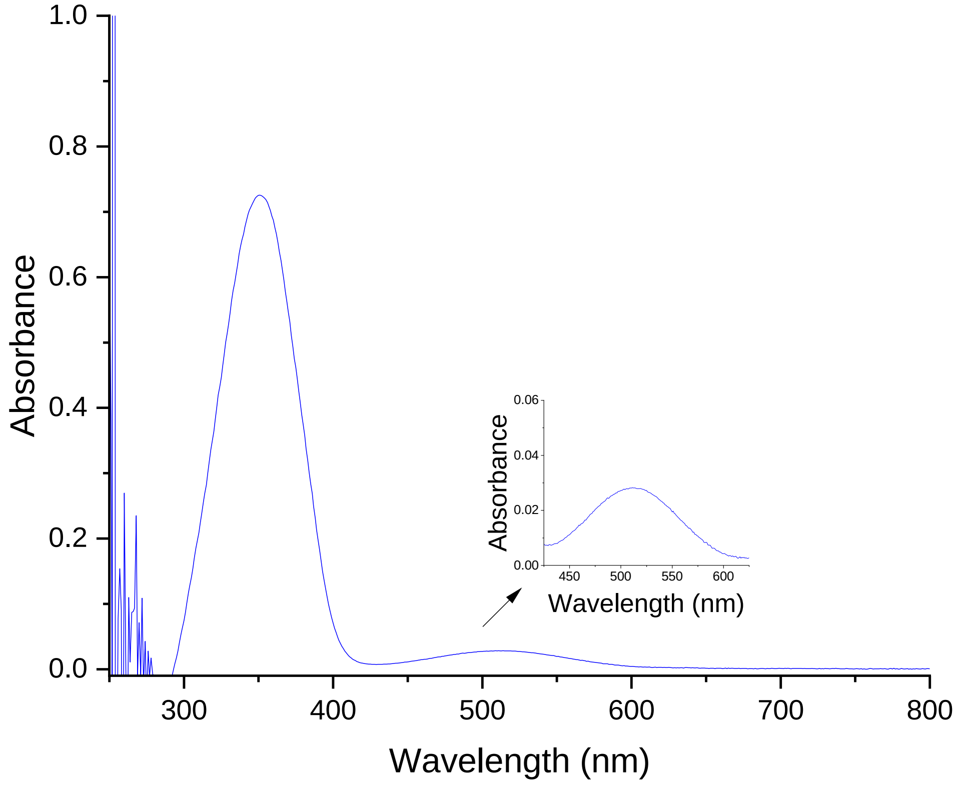

Figure S67. UV/Vis spectrum of **1-H** (5 x 10^{-5} M in toluene)

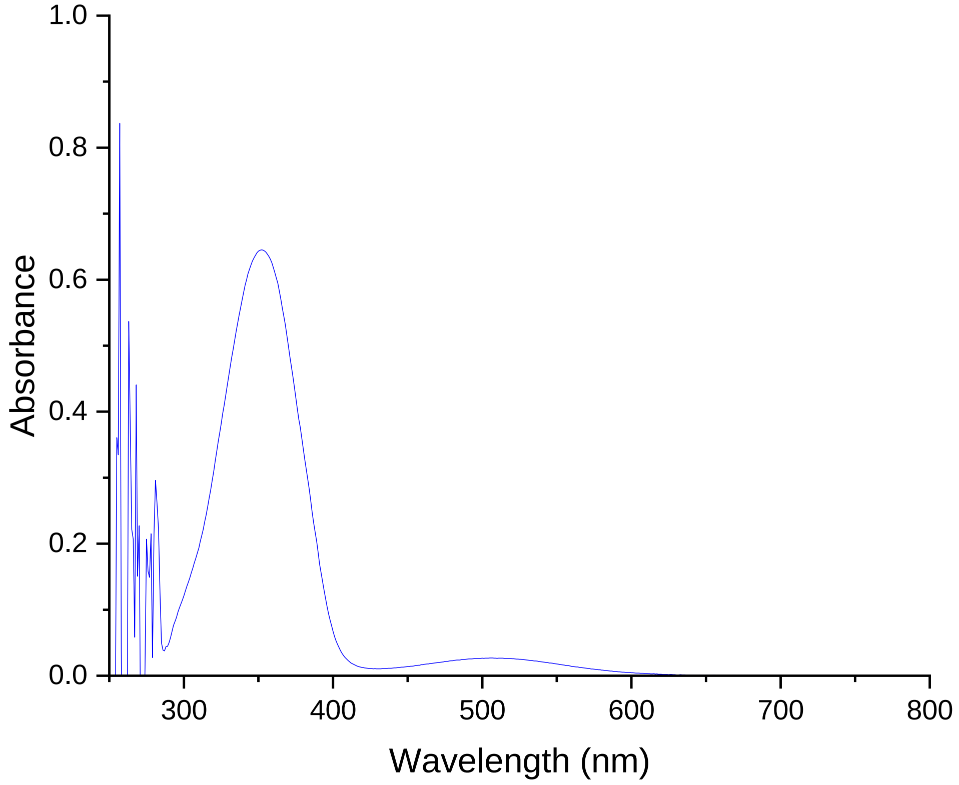

Figure S68. UV/Vis spectrum of **1-F** (5 x 10^{-5} M in toluene)

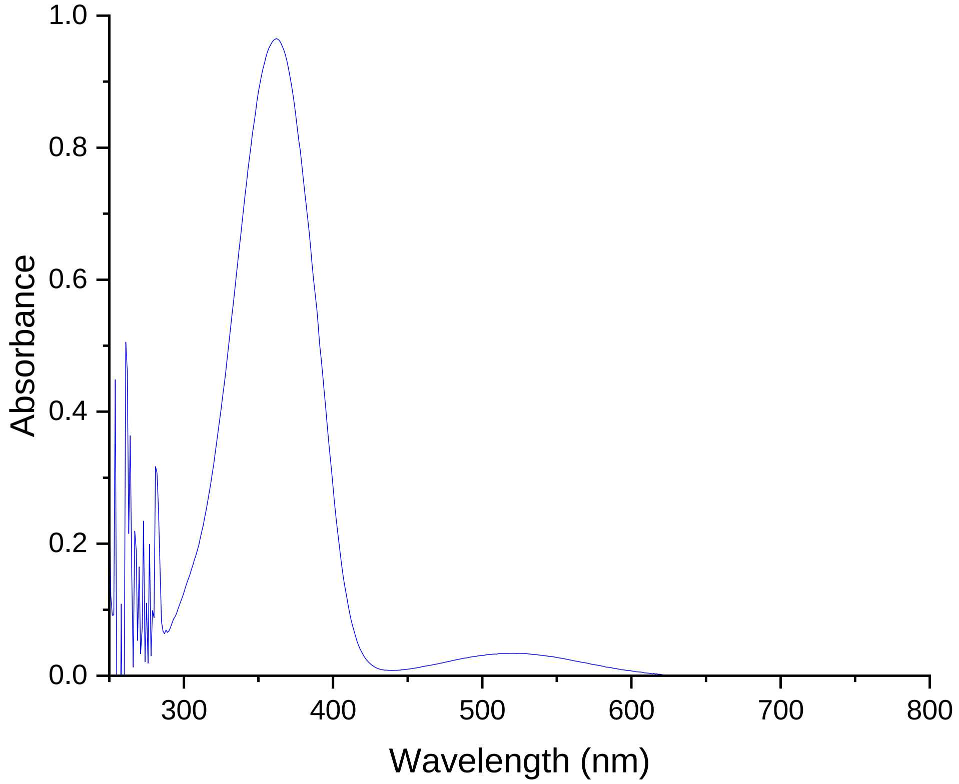

Figure S69. UV/Vis spectrum of **1-CF$_3$** (5 x 10^{-5} M in toluene)

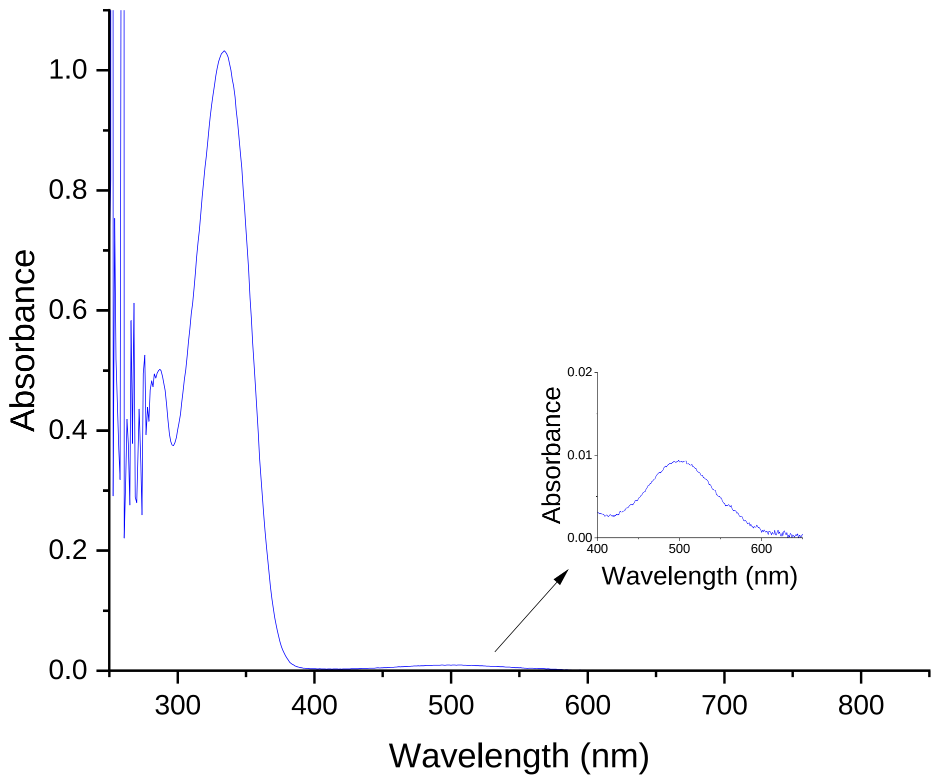

Figure S70. UV/Vis spectrum of **2-OMe** (5 x 10^{-5} M in toluene)

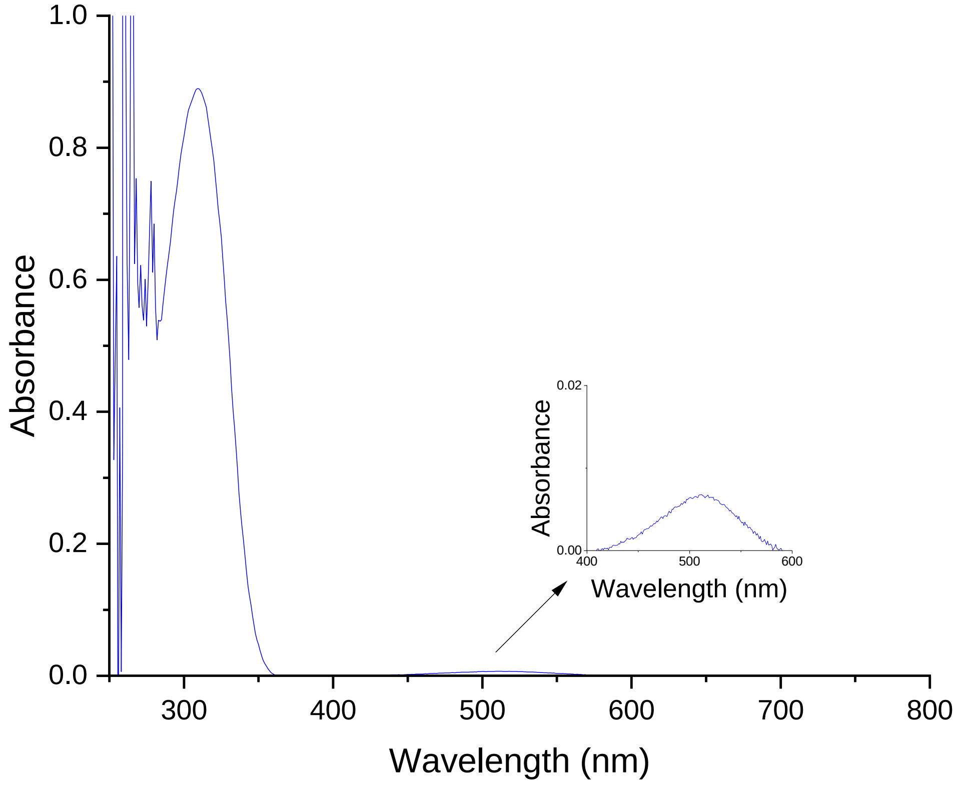

Figure S71. UV/Vis spectrum of **2-Me** (5 x 10^{-5} M in toluene)

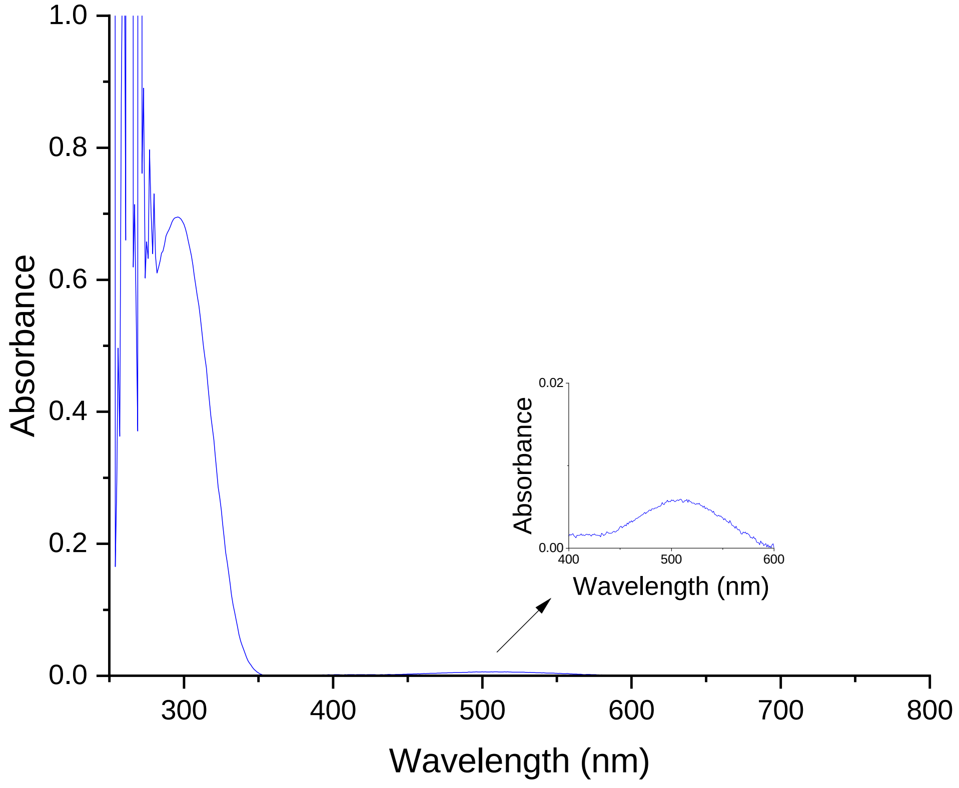

Figure S72. UV/Vis spectrum of **2-H** (5 x 10^{-5} M in toluene)

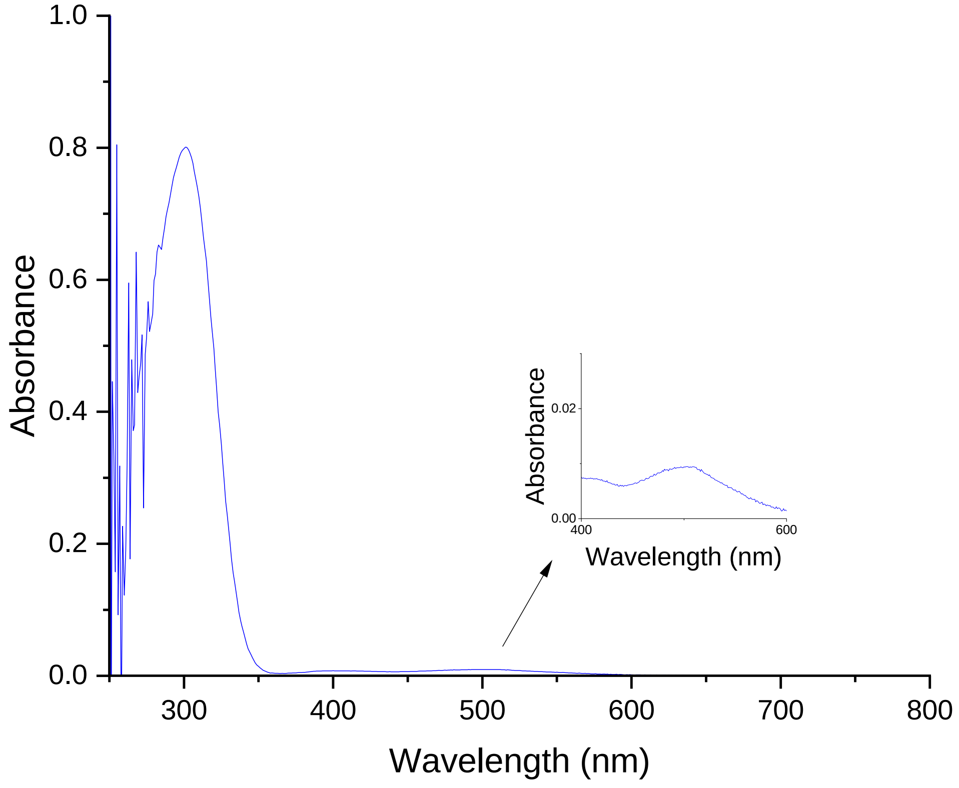

Figure S73. UV/Vis spectrum of **2-F** (5 x 10^{-5} M in toluene)

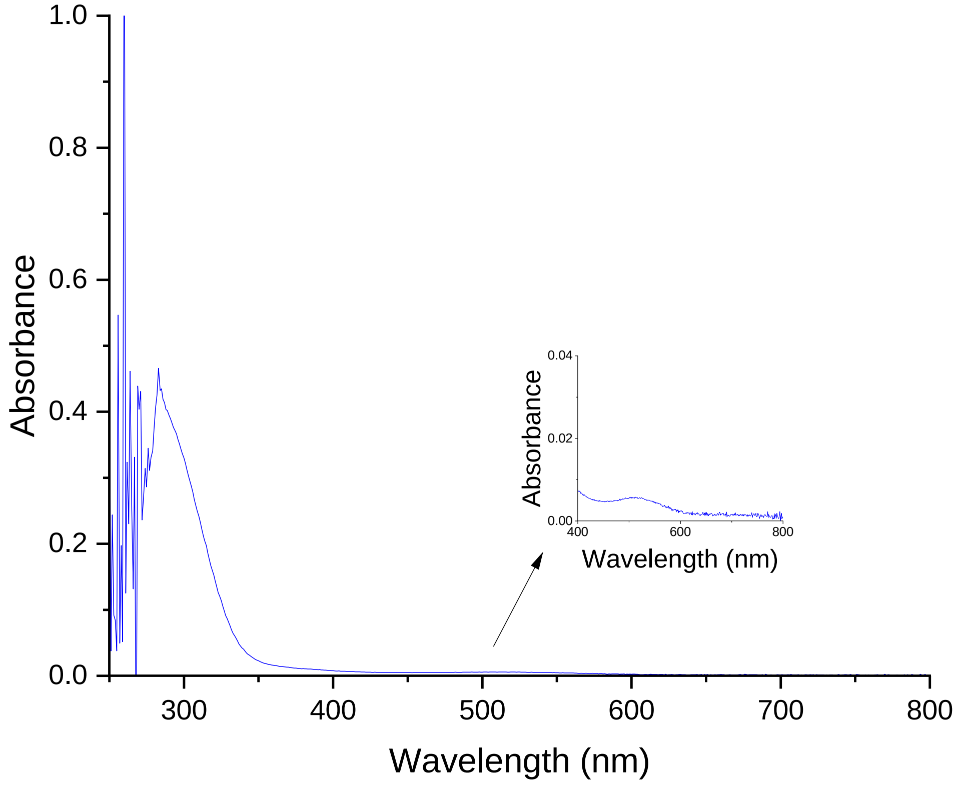

Figure S74. UV/Vis spectrum of **2-CF_3** (5 x 10^{-5} M in toluene

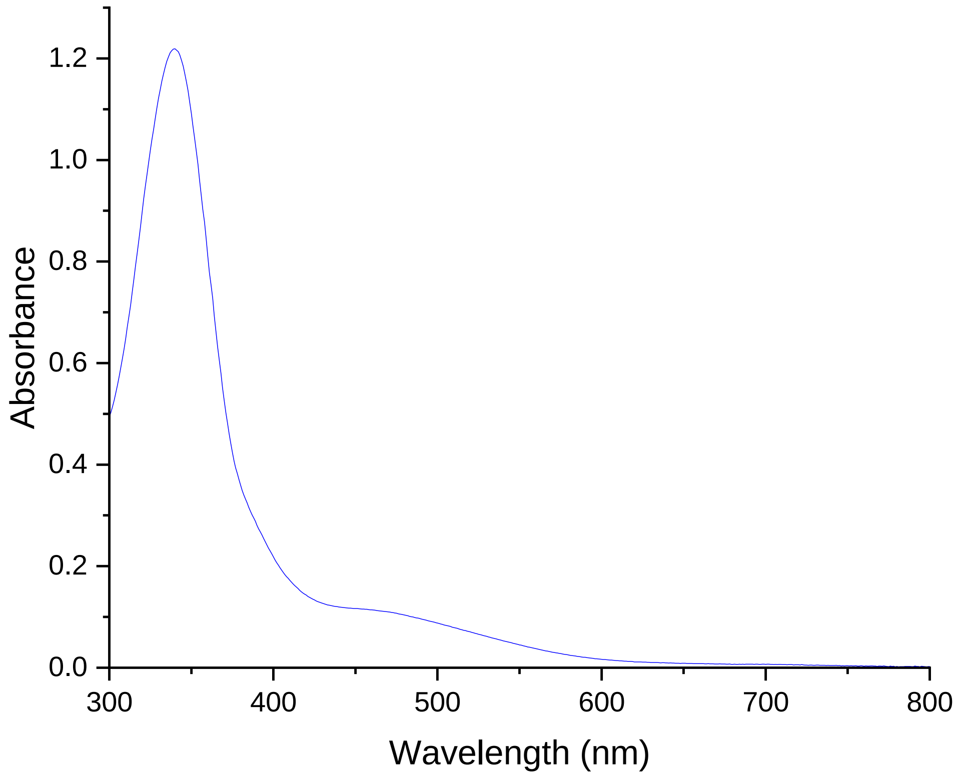

Figure S75. UV/Vis spectrum of **4-OMe** (5 x 10^{-5} M in DCM)

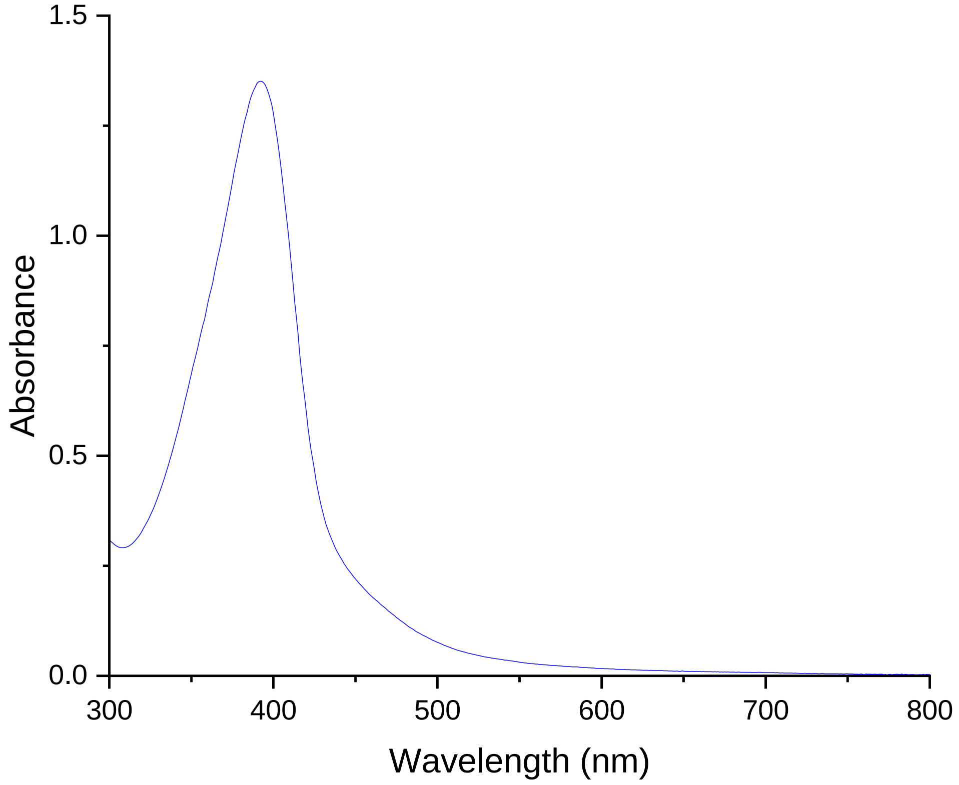

Figure S76. UV/Vis spectrum of **5-OMe** (5 x 10^{-5} M in DCM)

S2.3. High-Resolution Mass Spectra

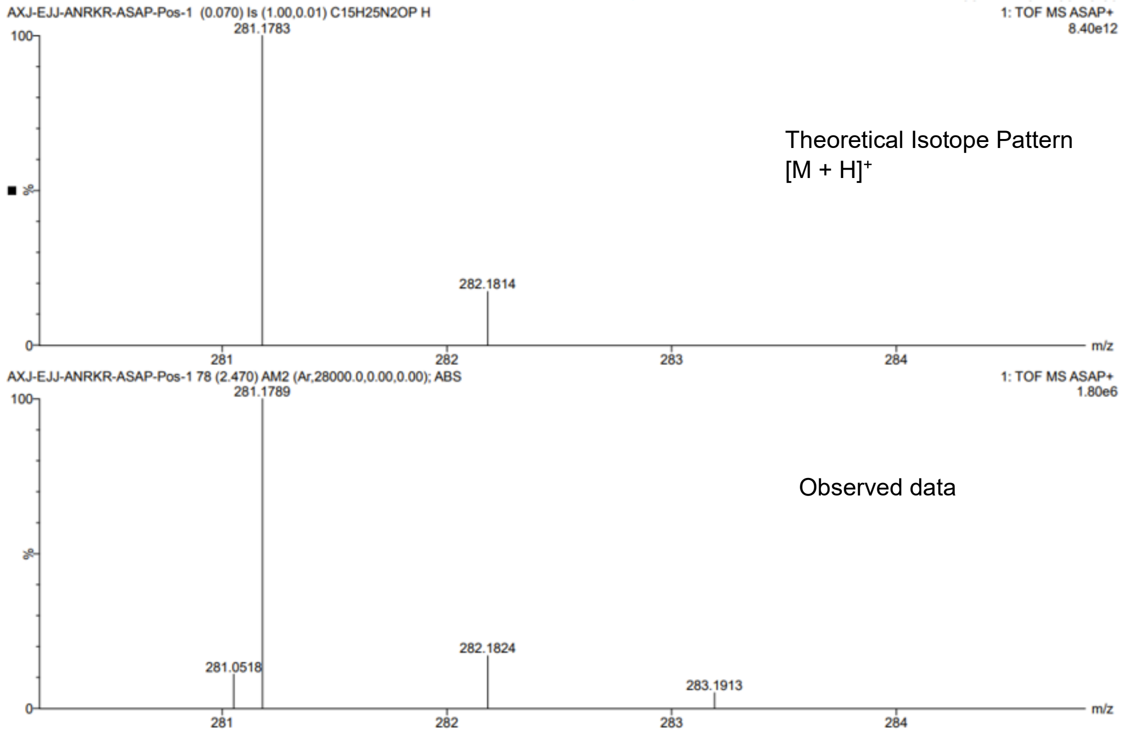

Figure S77. High-resolution Mass Spectrum of **1-OMe**

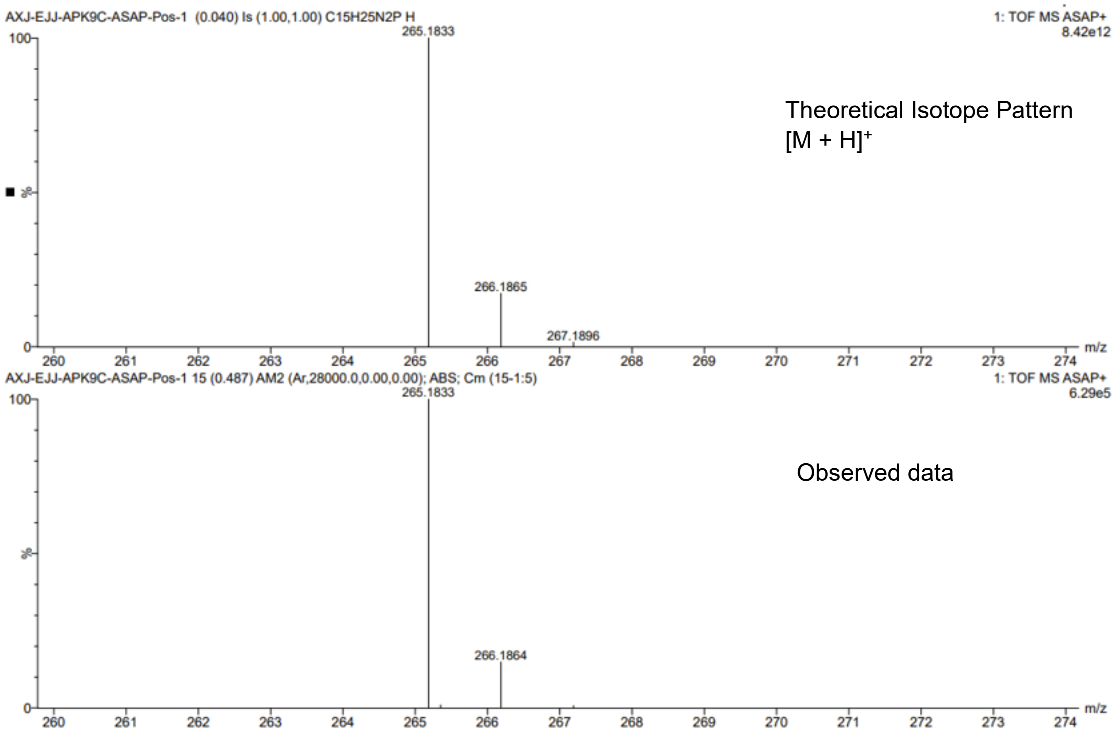

Figure S78. High-resolution Mass Spectrum of **1-Me**

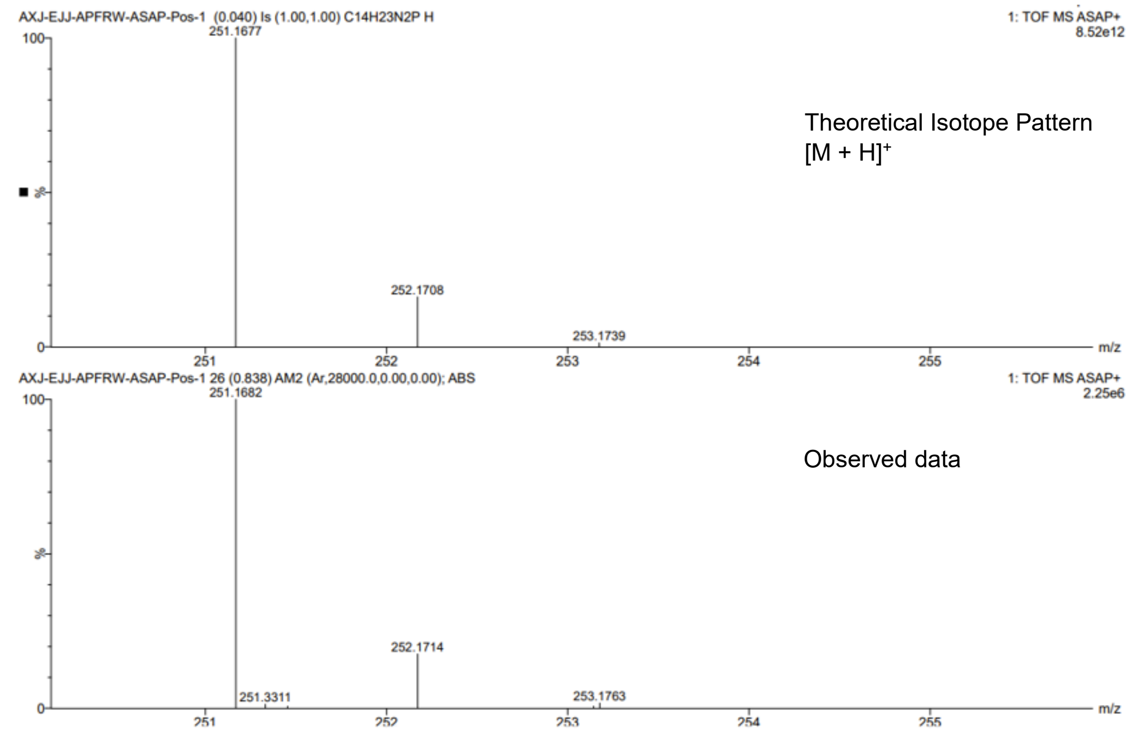

Figure S79. High-resolution Mass Spectrum of **1-H**

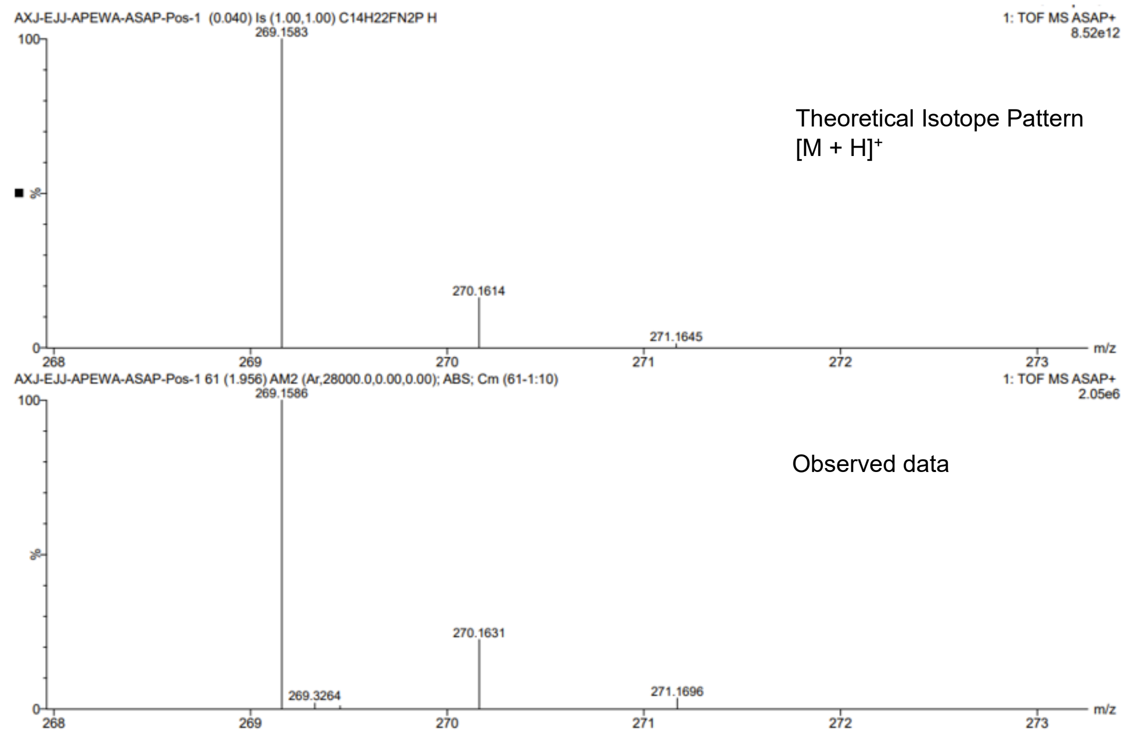

Figure S80. High-resolution Mass Spectrum of **1-F**

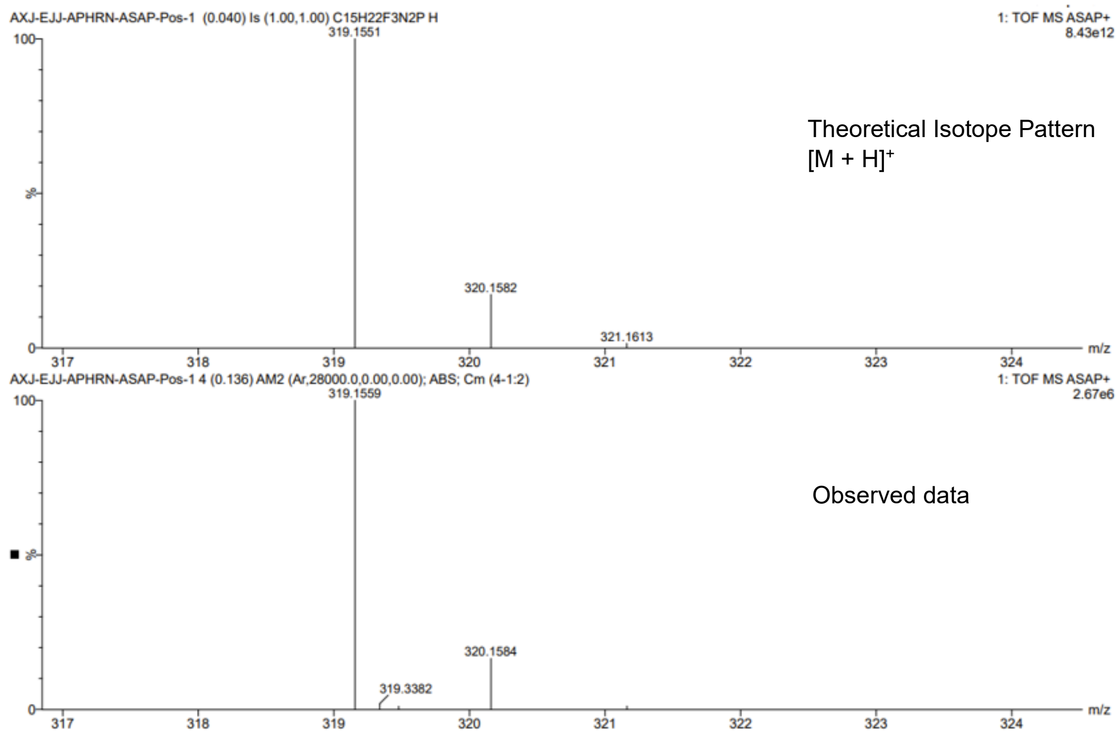

Figure S81. High-resolution Mass Spectrum of **1-CF_3**

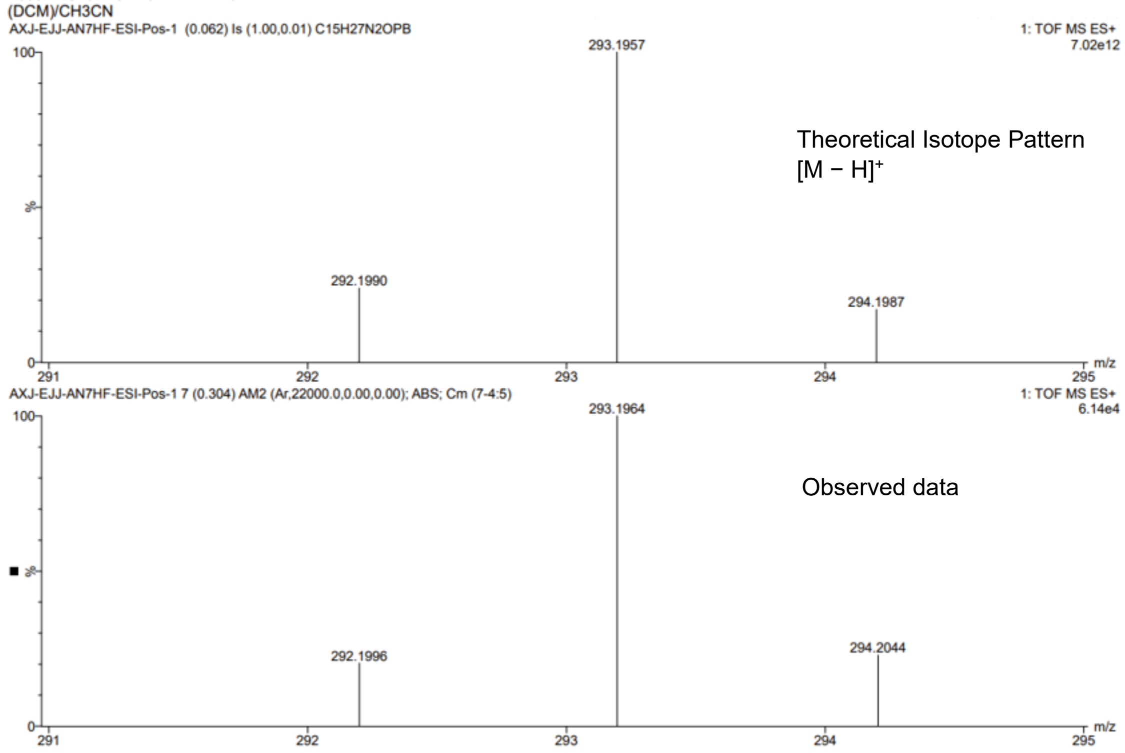

Figure S82. High-resolution Mass Spectrum of **2-OMe**

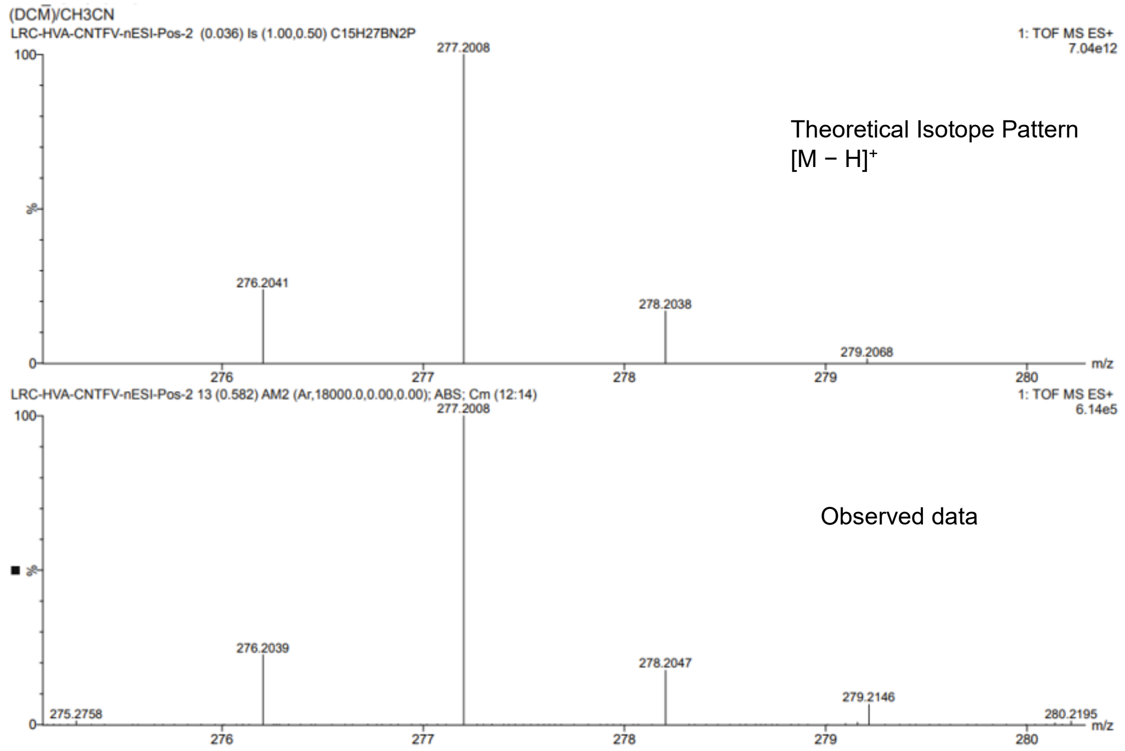

Figure S83. High-resolution Mass Spectrum of **2-Me**

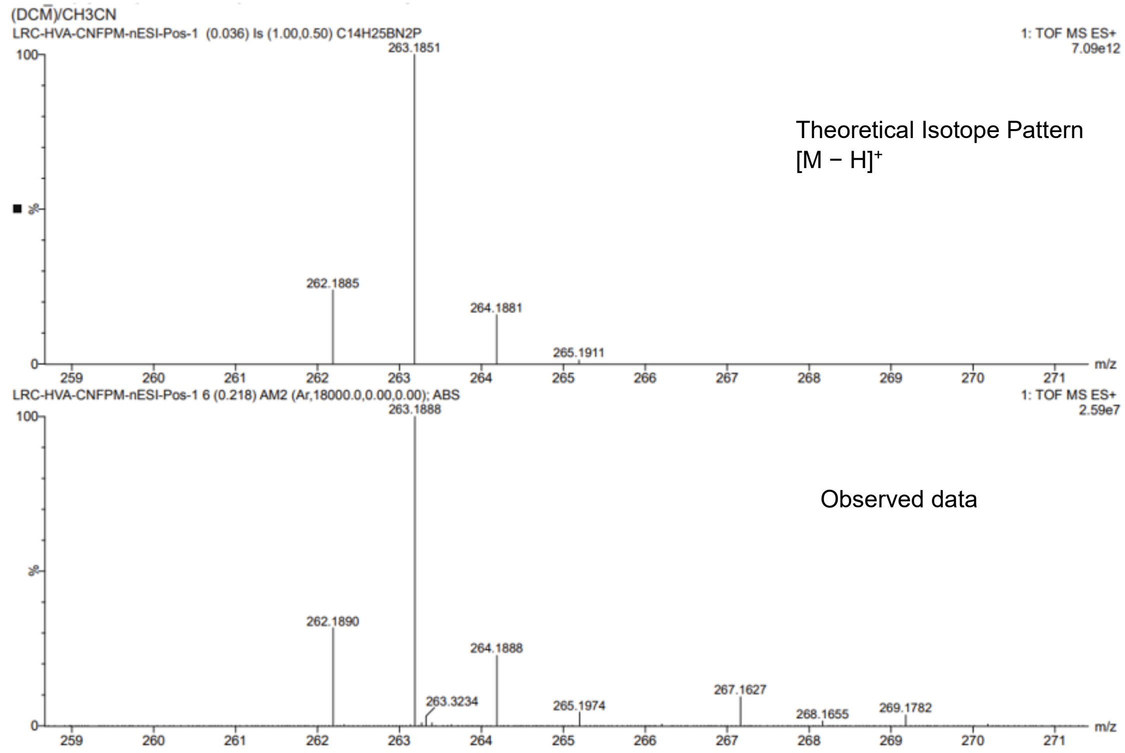

Figure S84. High-resolution Mass Spectrum of **2-H**

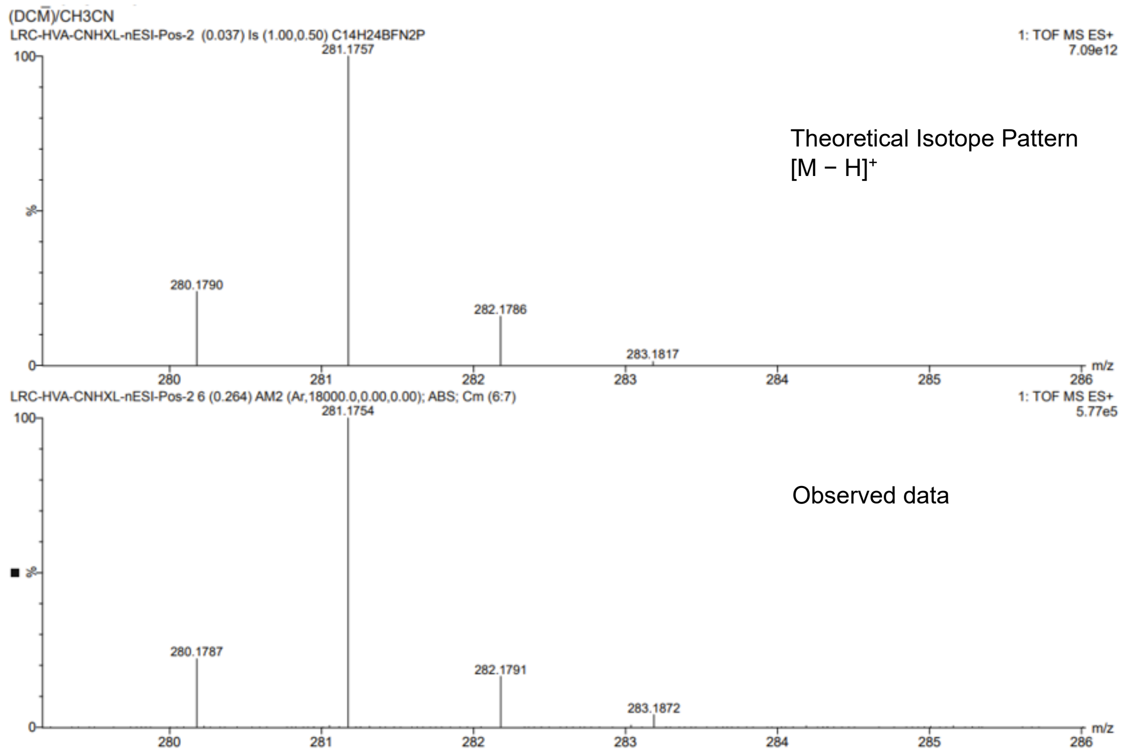

Figure S85. High-resolution Mass Spectrum of **2-F**

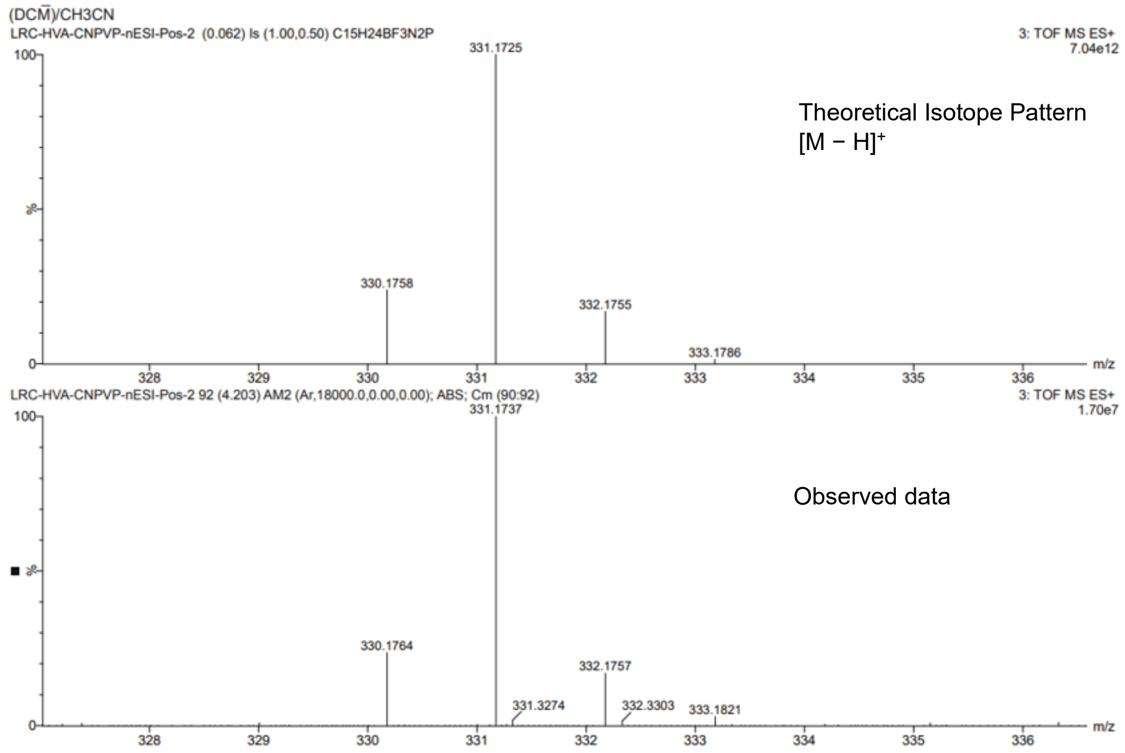

Figure S86. High-resolution Mass Spectrum of **2-CF_3**

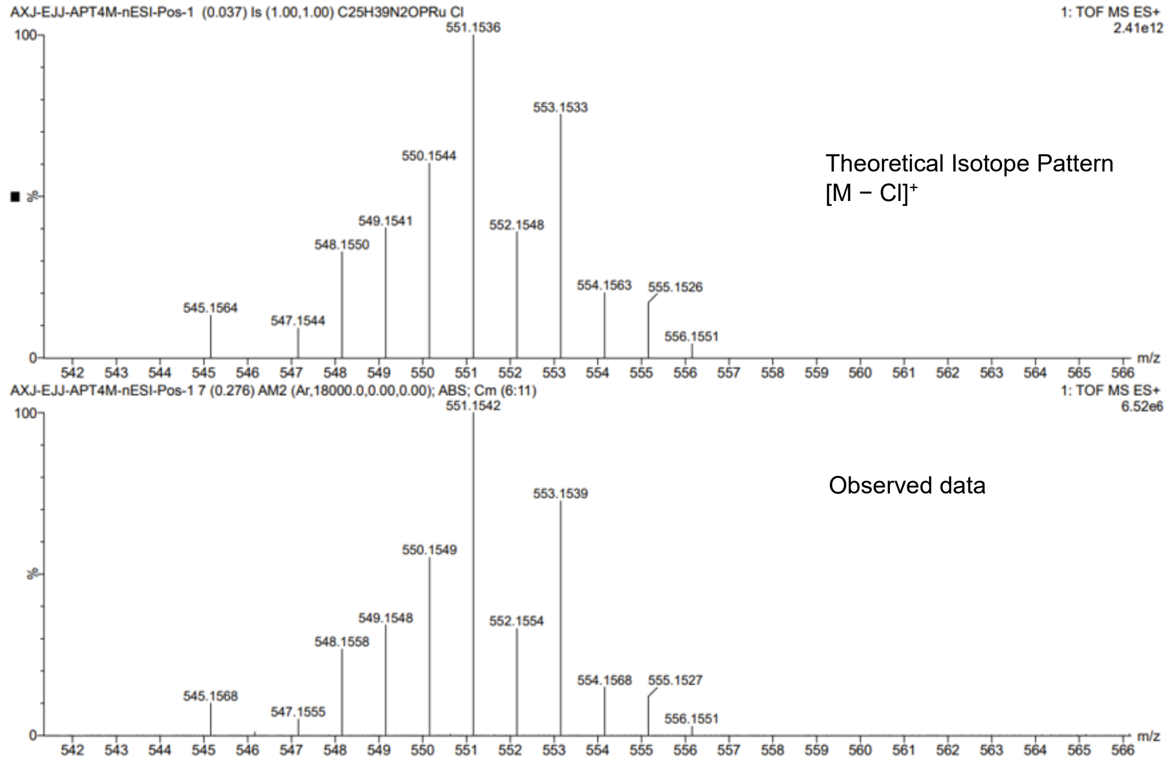

Figure S87. High-resolution Mass Spectrum of **4-OMe**

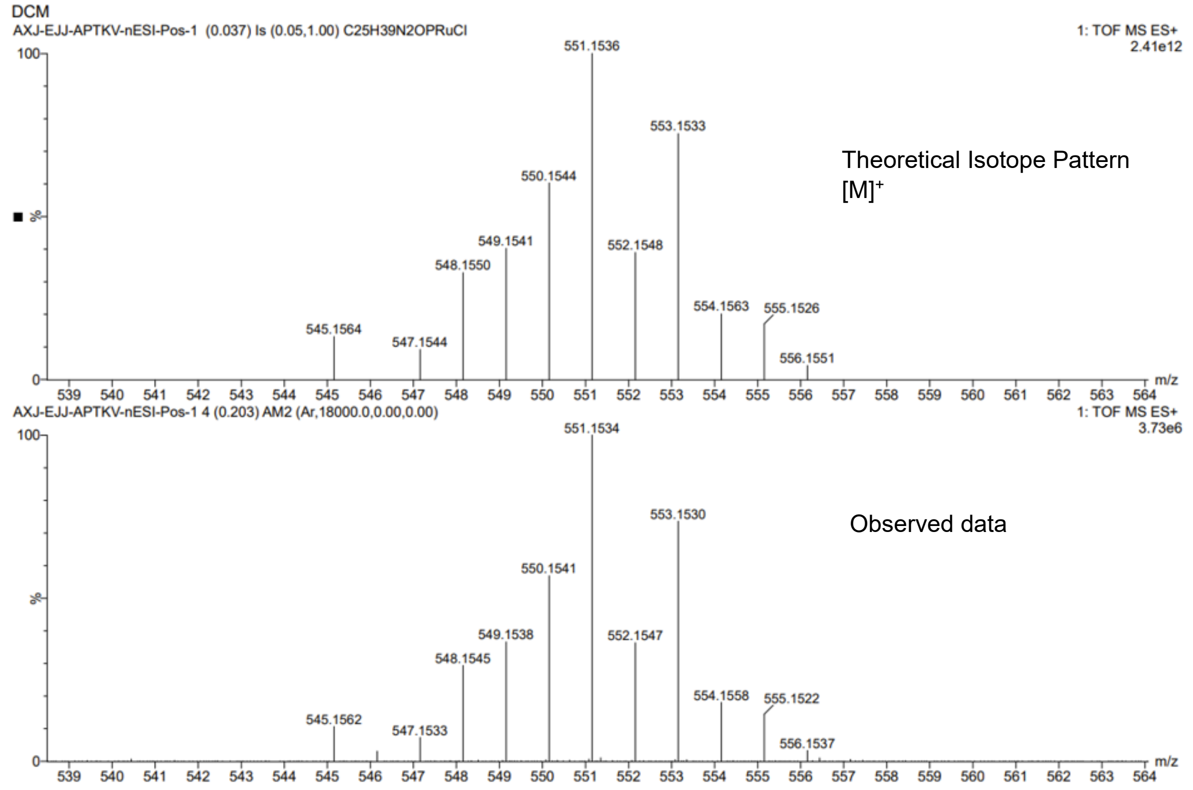

Figure S88. High-resolution Mass Spectrum of **5-OMe** (positive mode)

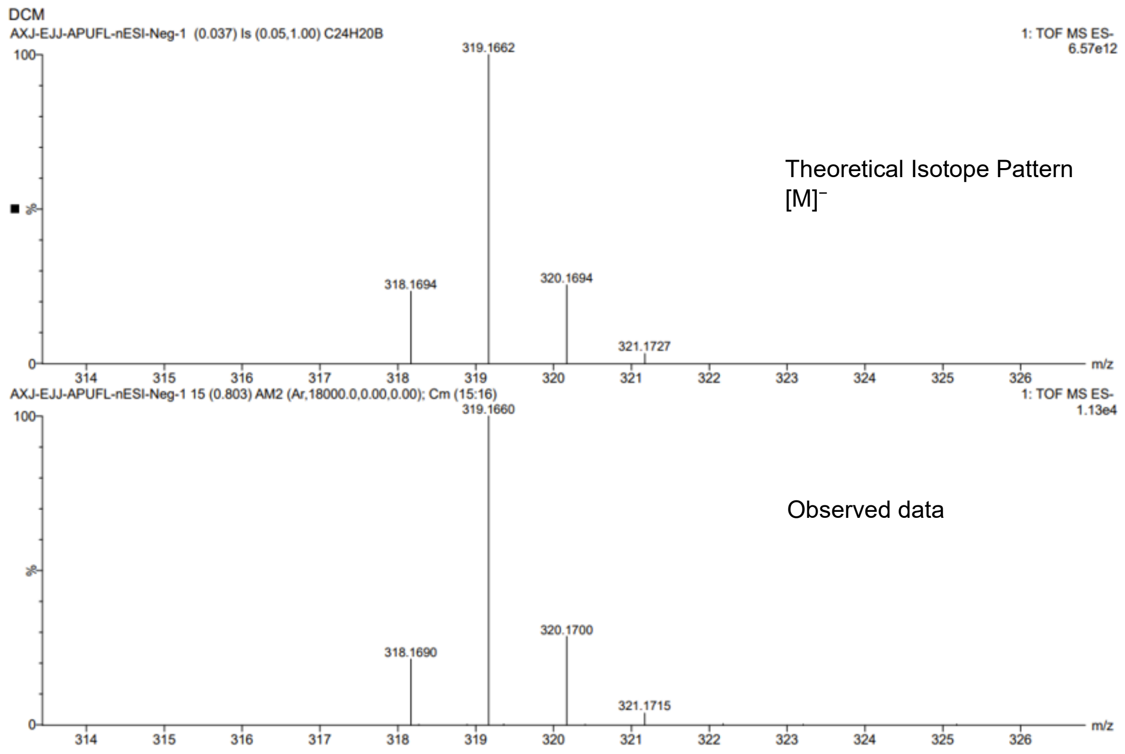

Figure S89. High-resolution Mass Spectrum of **5-OMe** (negative mode)

S2.4. IR Spectra

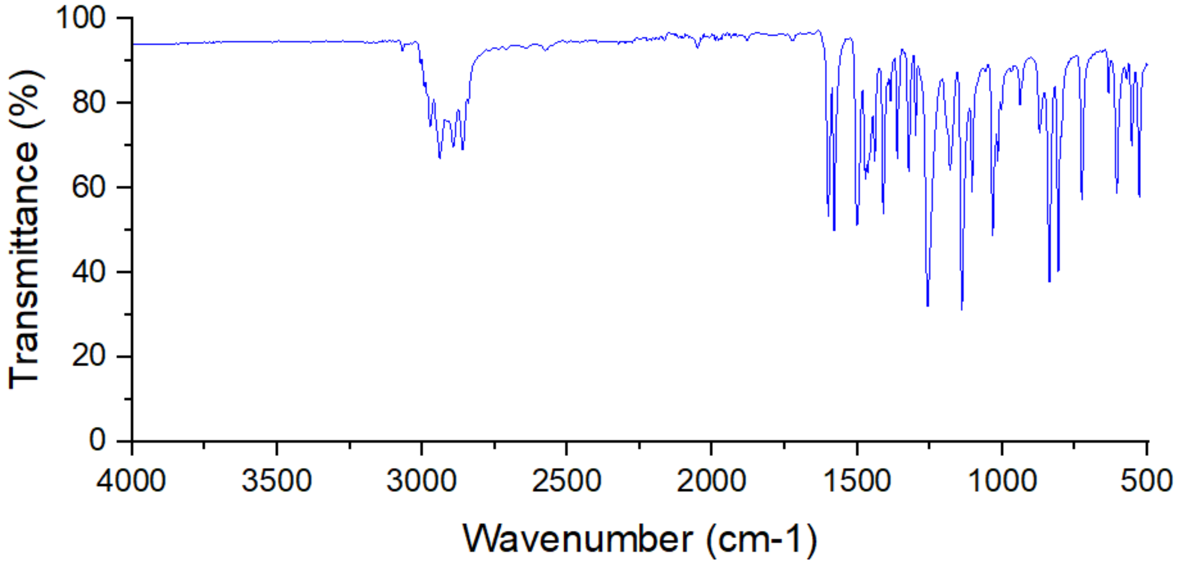

Figure S90. IR Spectrum of **1-OMe**

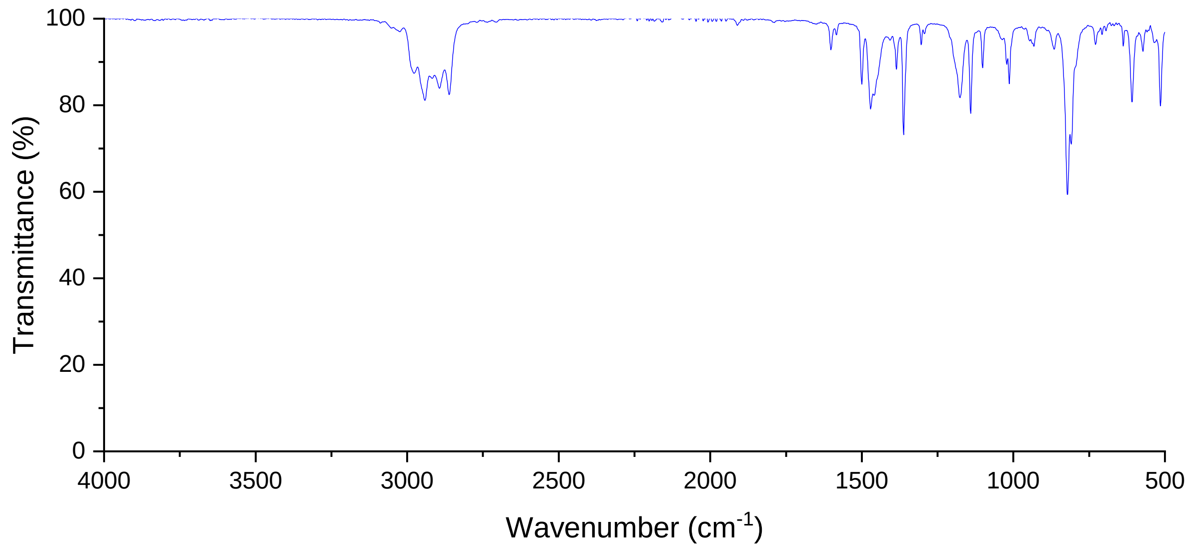

Figure S91. IR Spectrum of **1-Me**

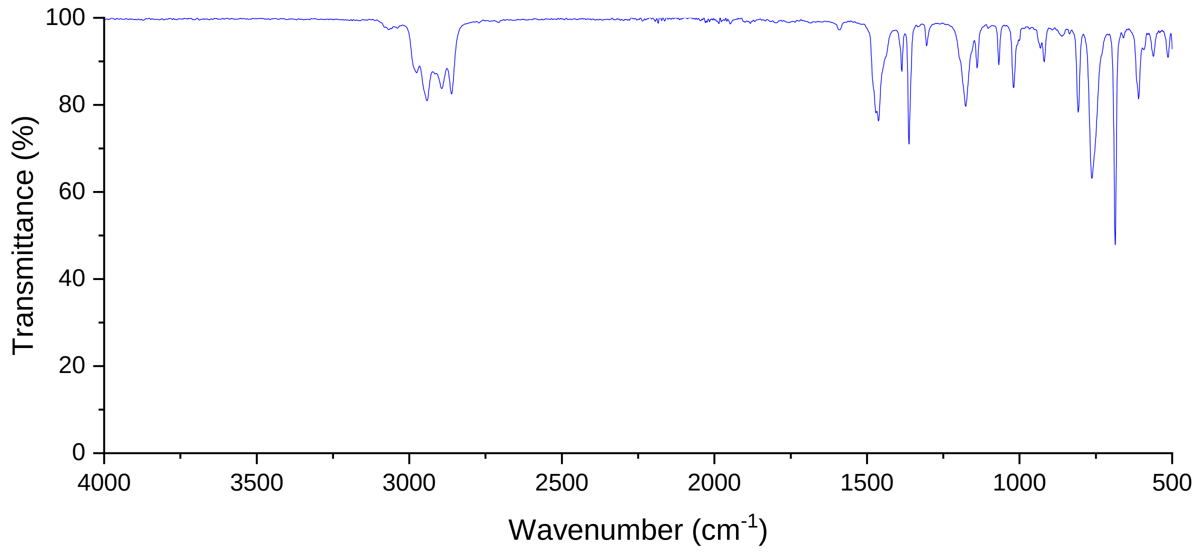

Figure S92. IR Spectrum of **1-H**

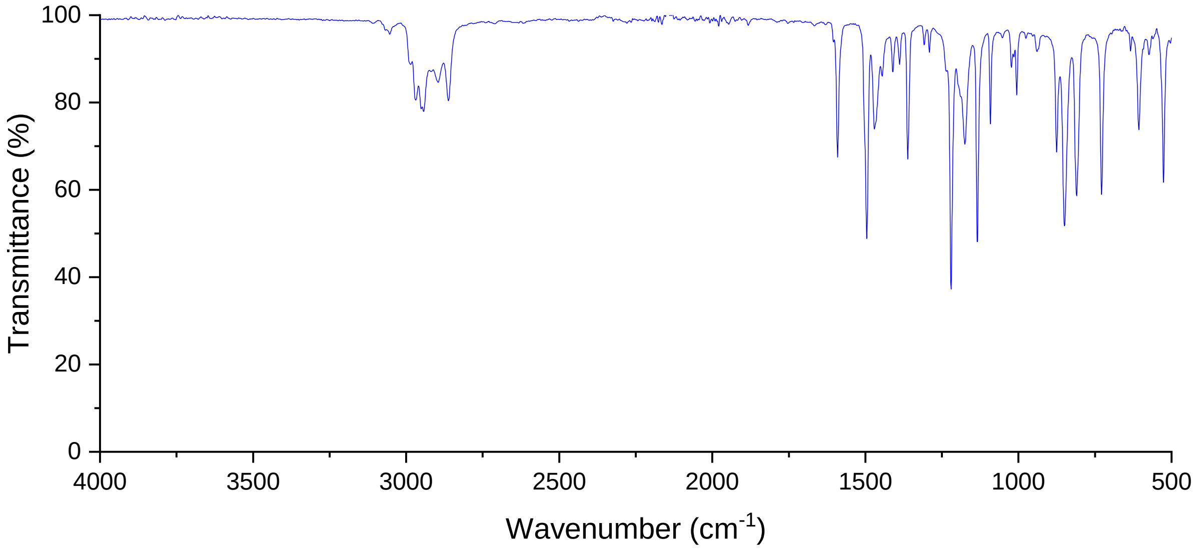

Figure S93. IR Spectrum of **1-F**

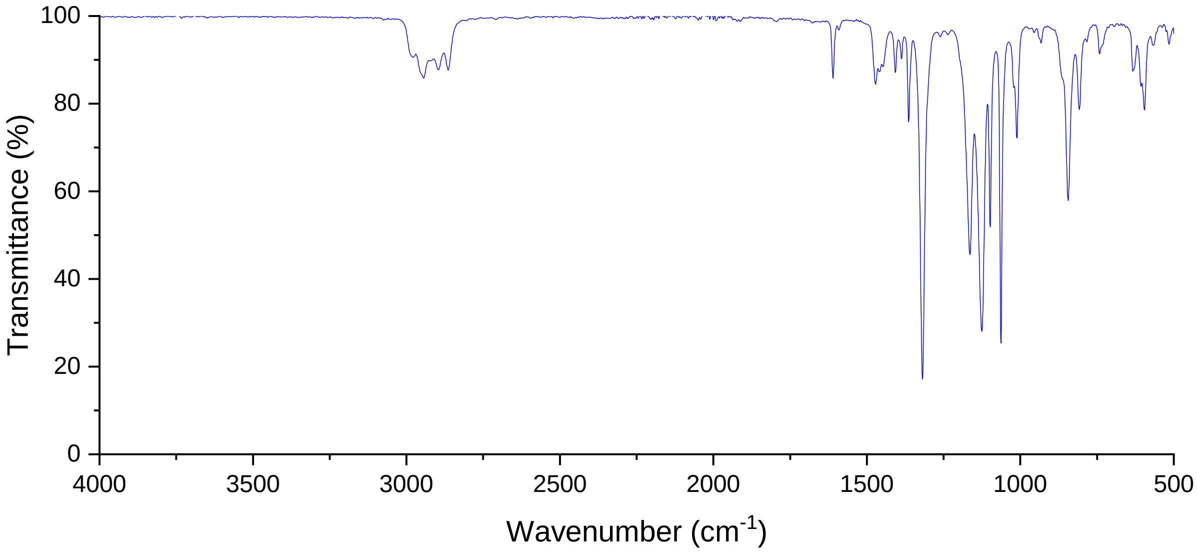

Figure S94. IR Spectrum of **1-CF$_3$**

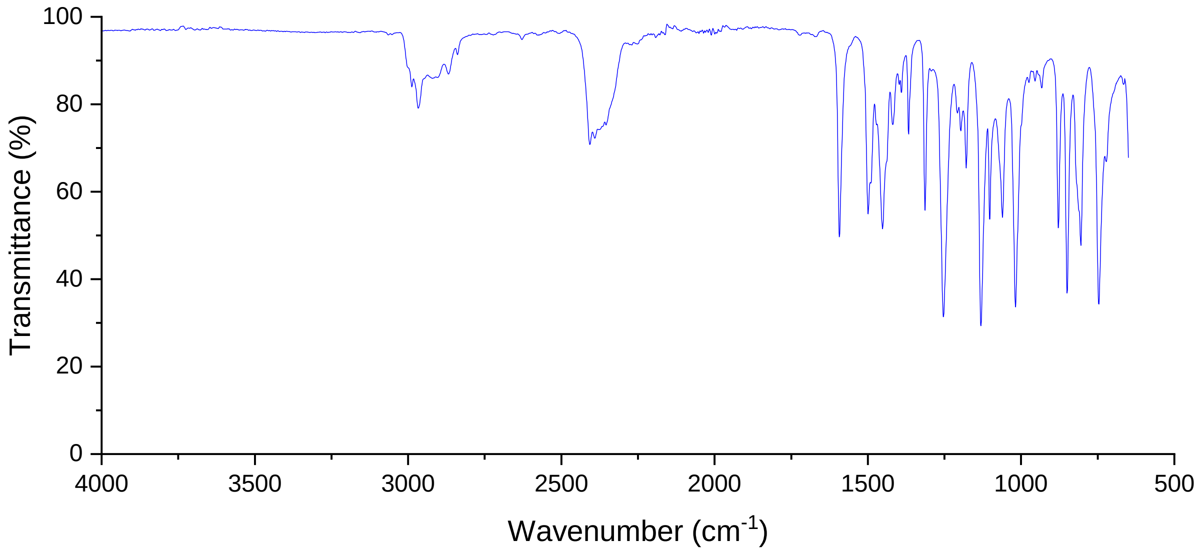

Figure S95. IR Spectrum of **2-OMe**

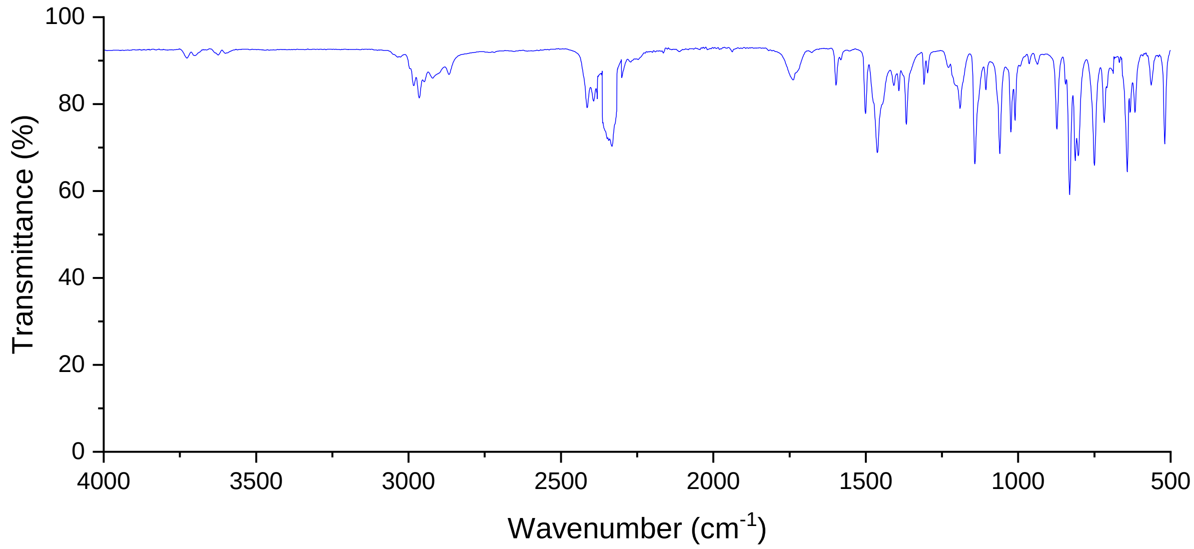

Figure S96. IR Spectrum of **2-Me**

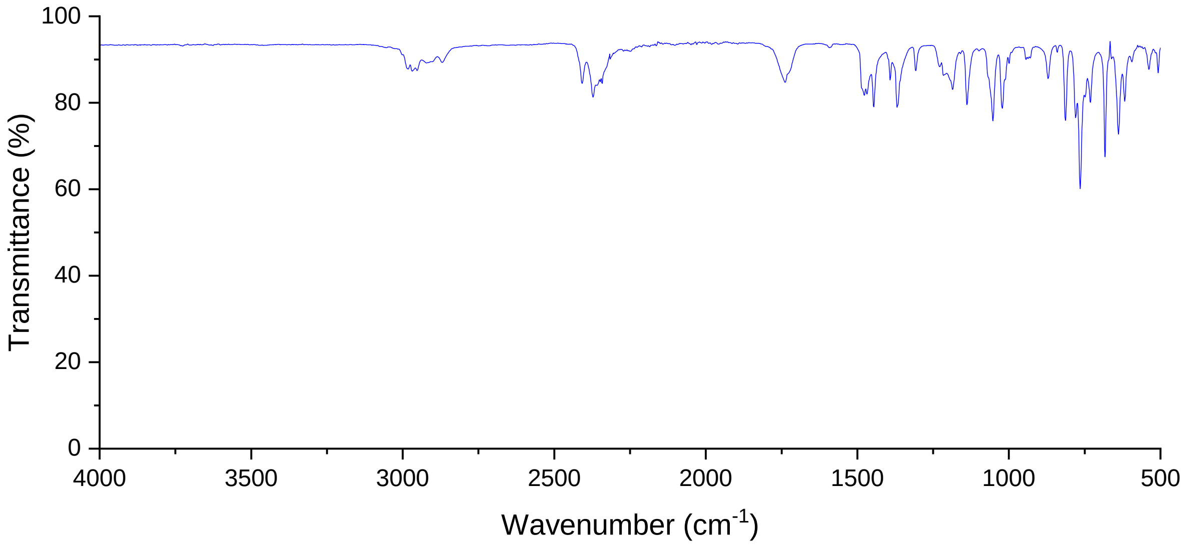

Figure S97. IR Spectrum of **2-H**

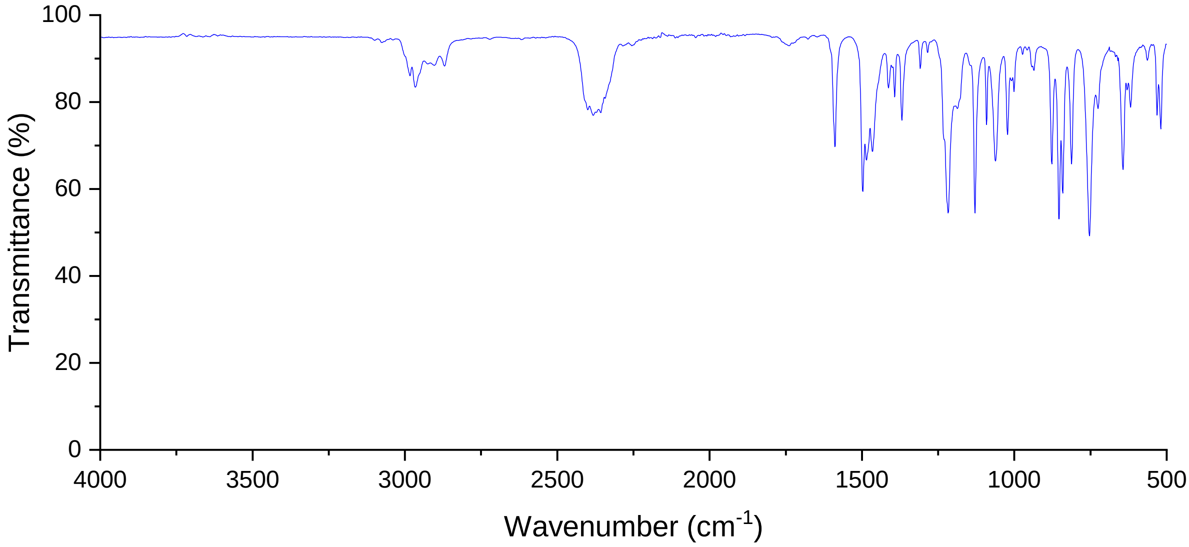

Figure S98. IR Spectrum of **2-F**

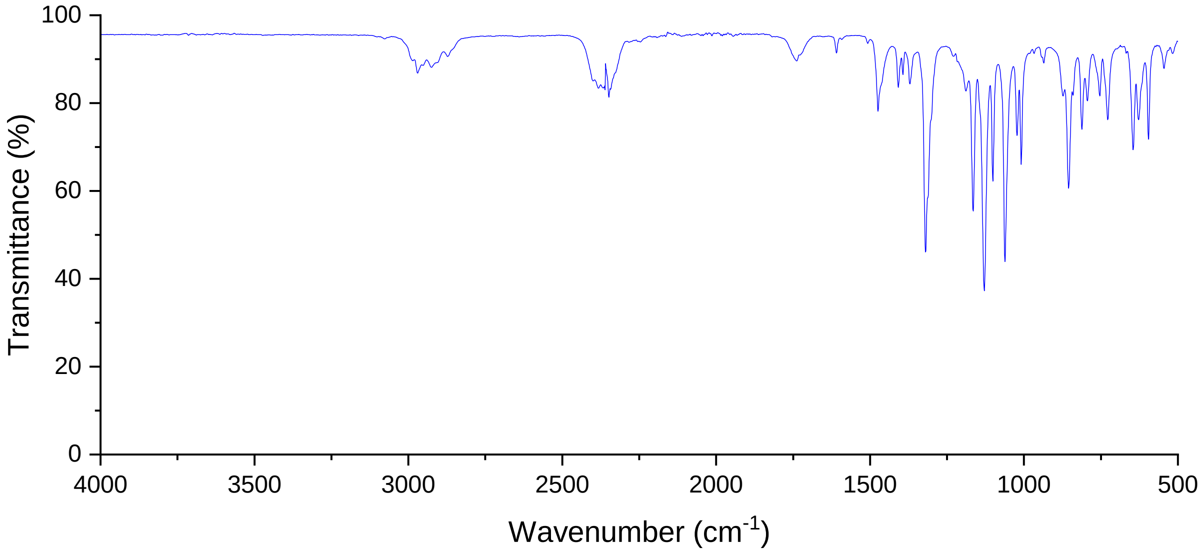

Figure S99. IR Spectrum of **2-CF_3**

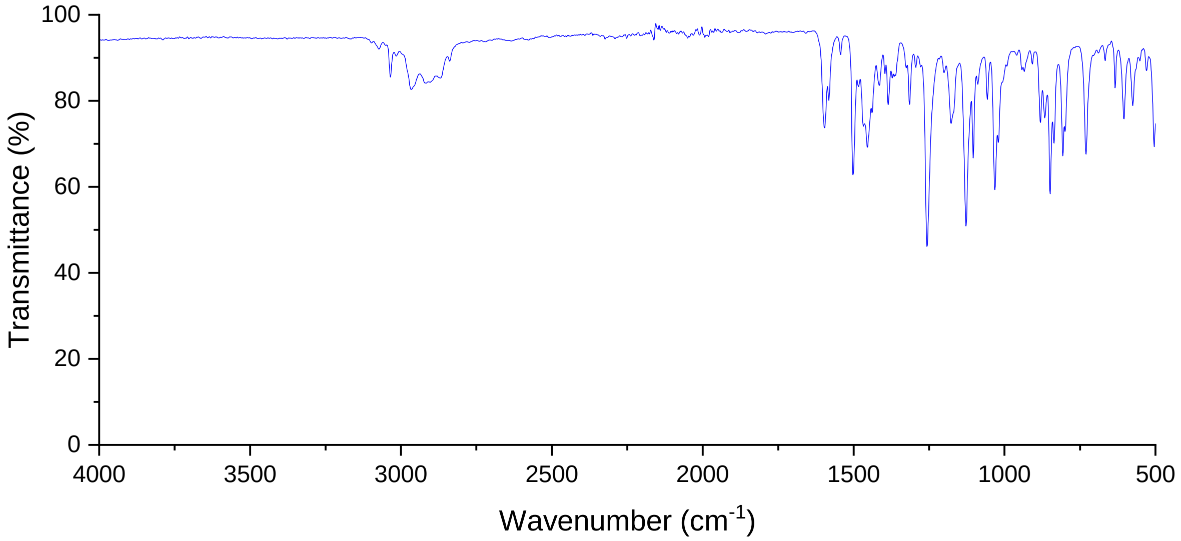

Figure S100. IR Spectrum of **4-OMe**

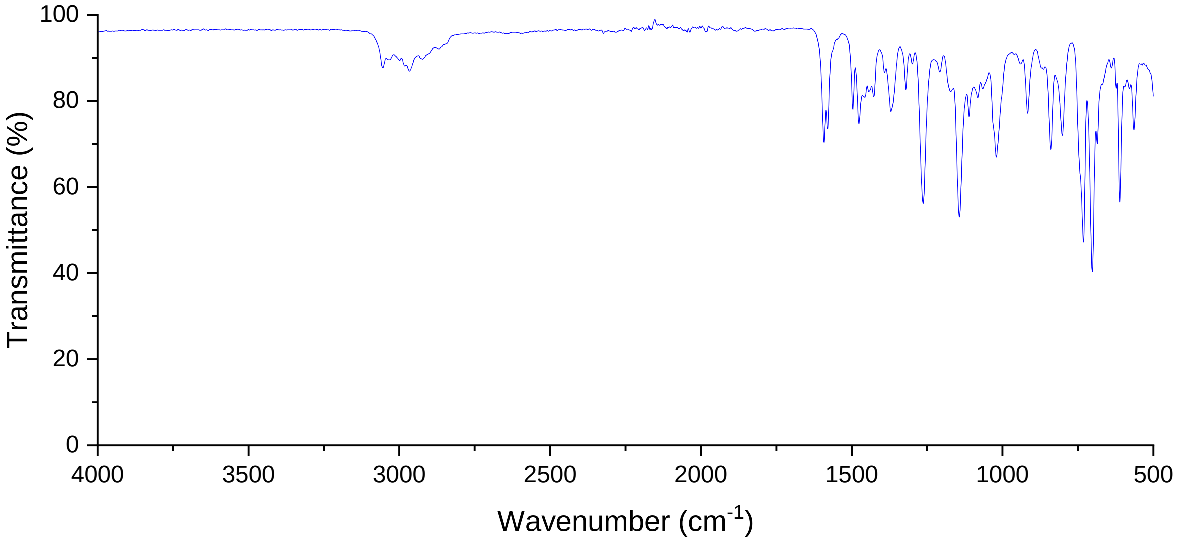

Figure S101. IR Spectrum of **5-OMe**

S3. Single Crystal X-ray Diffraction

Data collections were driven and processed, and absorption corrections were applied using CrysalisPro.[1] Using OLEX2[2] the structures were solved using SHELXT[3] and were refined by a full-matrix least-squares procedure on F^2 in SHELXL.[4] All non-hydrogen atoms were refined with anisotropic displacement parameters.

S3.1. Tables of Crystallographic Data and Structure Refinement

Table S1. Crystallographic data and structure refinement for **1-OMe, 2-OMe, 2-Me** and **2-H**.

Compound	1-OMe	2-OMe	2-Me	2-H
**Crystallographic Parameter**				
Identification code	EJJ200	EJJ047	EJJ_HVA	EDEC003
Empirical formula	$C_{15}H_{25}N_2OP$	$C_{15}H_{28}BN_2OP$	$C_{15}H_{28}BN_2P$	$C_{14}H_{26}BN_2P$
Formula weight	280.34	294.17	278.17	264.15
Temperature/K	120.00(10)	100.01(10)	99.94(18)	100.01(10)
Crystal system	triclinic	monoclinic	monoclinic	monoclinic
Space group	P-1	$P2_1/n$	$P2_1/n$	$P2_1/n$
a/Å	6.3217(3)	10.7092(2)	12.5144(6)	8.0589(4)
b/Å	7.0360(3)	14.1255(3)	10.7260(3)	24.6162(13)
c/Å	19.1910(6)	11.1853(2)	13.6865(6)	8.0997(5)
α/°	98.292(3)	90	90	90
β/°	95.150(3)	97.554(2)	112.488(5)	95.435(5)
γ/°	102.444(3)	90	90	90
Volume/Å^3	818.36(6)	1677.35(6)	1697.43(13)	1599.59(15)
Z	2	4	4	4
ρ_{calc}g/cm^3	1.138	1.165	1.089	1.097
μ/mm^{-1}	1.441	1.416	1.328	1.386
F(000)	304.0	640.0	608.0	576.0
Crystal size/mm^3	0.273 × 0.124 × 0.025	0.205 × 0.173 × 0.116	0.2 × 0.16 × 0.1	0.228 × 0.128 × 0.077
Radiation	Cu Kα (λ = 1.54184)	Cu Kα (λ = 1.54184)	Cu Kα (λ = 1.54184)	Cu Kα (λ = 1.54184)
2Θ range for data collection/°	9.388 to 154.532	10.142 to 146.104	8.152 to 146.252	7.182 to 146.294
Index ranges	-7 ≤ h ≤ 7, -8 ≤ k ≤ 8, -24 ≤ l ≤ 24	-11 ≤ h ≤ 13, -17 ≤ k ≤ 17, -13 ≤ l ≤ 13	-15 ≤ h ≤ 15, -8 ≤ k ≤ 13, -16 ≤ l ≤ 16	-9 ≤ h ≤ 9, -30 ≤ k ≤ 22, -8 ≤ l ≤ 9
Reflections collected	26935	31186	9714	6582
Independent reflections	3399 [R_{int} = 0.0582, R_{sigma} = 0.0318]	3331 [R_{int} = 0.0392, R_{sigma} = 0.0160]	3327 [R_{int} = 0.0339, R_{sigma} = 0.0336]	3105 [R_{int} = 0.0185, R_{sigma} = 0.0226]
Data/restraints/parameters	3399/0/179	3331/0/200	3327/1/191	3105/0/181
Goodness-of-fit on F^2	1.047	1.058	1.021	1.054
Final R indexes [I>=2σ (I)]	R_1 = 0.0379, wR_2 = 0.0949	R_1 = 0.0310, wR_2 = 0.0805	R_1 = 0.0354, wR_2 = 0.0833	R_1 = 0.0299, wR_2 = 0.0758
Final R indexes [all data]	R_1 = 0.0465, wR_2 = 0.1000	R_1 = 0.0332, wR_2 = 0.0827	R_1 = 0.0439, wR_2 = 0.0887	R_1 = 0.0329, wR_2 = 0.0782
Largest diff peak/hole / e Å^{-3}	0.40/-0.25	0.30/-0.29	0.24/-0.31	0.30/-0.25

Table S2. Crystallographic data and structure refinement for **2-F**, **2-CF3**, **4-OMe** and **5-OMe**.

Compound	2-F	2-CF$_3$	4-OMe	5-OMe
**Crystallographic Parameter**				
Identification code	EJJ049	EJJ180	EJJ123_3	EJJ214
Empirical formula	$C_{14}H_{25}BFN_2P$	$C_{15}H_{25}BF_3N_2P$	$C_{25}H_{39}Cl_2N_2OPRu$	$C_{49}H_{59}BN_2OPClRu$
Formula weight	282.14	332.15	586.52	870.28
Temperature/K	100.01(10)	120.03(10)	99.95(15)	100.00(10)

Crystal system	Monoclinic	monoclinic	triclinic	monoclinic
Space group	Ia	$P2_1/c$	P-1	$P2_1/c$
a/Å	14.3543(3)	14.5020(4)	9.2219(3)	16.53190(10)
b/Å	20.1151(3)	16.3948(6)	9.3766(4)	11.70170(10)
c/Å	22.8612(3)	15.3881(5)	16.9666(7)	23.41560(10)
α/°	90	90	102.406(3)	90
β/°	91.138(2)	93.858(3)	97.481(3)	109.3460(10)
γ/°	90	90	108.658(4)	90
Volume/$Å^3$	6599.60(19)	3650.3(2)	1325.80(10)	4274.01(5)
Z	16	8	2	4
$\rho_{calc}g/cm^3$	1.136	1.209	1.469	1.352
μ/mm^{-1}	1.464	1.562	7.359	4.189
F(000)	2432.0	1408.0	608.0	1824.0
Crystal size/mm^3	0.314 × 0.166 × 0.069	0.245 × 0.151 × 0.086	0.3 × 0.048 × 0.033	0.148 × 0.097 × 0.025
Radiation	Cu Kα (λ = 1.54184)	Cu Kα (λ = 1.54184)	Cu Kα (λ = 1.54184)	Cu Kα (λ = 1.54184)
2Θ range for data collection/°	7.568 to 146.03	7.89 to 145.212	10.302 to 136.498	5.666 to 157.958
Index ranges	-14 ≤ h ≤ 17, -24 ≤ k ≤ 24, -28 ≤ l ≤ 28	-13 ≤ h ≤ 17, -20 ≤ k ≤ 19, -17 ≤ l ≤ 19	-11 ≤ h ≤ 11, -9 ≤ k ≤ 11, -20 ≤ l ≤ 20	-20 ≤ h ≤ 21, -14 ≤ k ≤ 14, -27 ≤ l ≤ 29
Reflections collected	38387	18188	14013	108193
Independent reflections	11296 [R_{int} = 0.0280, R_{sigma} = 0.0238]	7072 [R_{int} = 0.0379, R_{sigma} = 0.0474]	4830 [R_{int} = 0.0602, R_{sigma} = 0.0580]	8805 [R_{int} = 0.0424, R_{sigma} = 0.0181]
Data/restraints/parameters	11296/11/758	7072/4/433	4830/0/299	8805/0/515
Goodness-of-fit on F^2	1.023	1.015	1.063	1.085
Final R indexes [I>=2σ (I)]	R_1 = 0.0380, wR_2 = 0.1029	R_1 = 0.0426, wR_2 = 0.1011	R_1 = 0.0515, wR_2 = 0.1268	R_1 = 0.0265, wR_2 = 0.0679
Final R indexes [all data]	R_1 = 0.0397, wR_2 = 0.1049	R_1 = 0.0631, wR_2 = 0.1148	R_1 = 0.0633, wR_2 = 0.1349	R_1 = 0.0287, wR_2 = 0.0692
Largest diff. peak/hole / e $Å^{-3}$	0.35/-0.24	0.54/-0.24	2.51/-1.46	0.42/-0.63
Flack parameter	0.448(19)	-	-	-

S3.2. Crystal Structure Determinations

Unless otherwise stated, the single-crystal X-ray diffraction studies were carried out as detailed in section S1.1. All hydrogen atoms not bonded to boron were fixed as riding models and the isotropic thermal parameters (U_{iso}) were based on the U_{eq} of the parent atom.

For **2-OMe**, the hydrogen atoms bonded to B(1) were located in the electron density and freely refined.

For **2-Me**, the hydrogen atoms bonded to B(1) were located in the electron density and freely refined, one of which is subject to B-H bond length restraints.

For **2-H**, the hydrogen atoms bonded to B(1) were located in the electron density and freely refined.

For **2-F**, the structure occupies a non-centrosymmetric space group and has been refined as an inversion twin, with the refined percentage ratio of domains being 55.2 (19) : 44.8 (19). The hydrogen atoms bonded to B(1), B(101), B(201) and B(301) were located in the electron density and freely refined, some of which are subject to B-H bond length restraints. The structure of **2-F** contains four crystallographically independent molecules (only one shown in

Figure 1E for clarity).

For **2-CF_3**, the hydrogen atoms bonded to B(1) and B(101) were located in the electron density and freely refined, four of which are subject to B-H bond length restraints. The structure of **2-CF_3** contains two crystallographically independent molecules (only one shown in Figure 1F for clarity).

Deposition numbers 2358664 (for **1-OMe**), 2358660 (for **2-OMe**), 2358661 (for **2-Me**), 2358659 (for **2-H**), 2358663 (for **2-F**) 2358666 (for **2-CF_3**), 2358665 (for **4-OMe**) and 2358662 (for **5-OMe**) contains the supplementary crystallographic data for this paper. These can be obtained free of charge by the joint Cambridge Crystallographic Data Centre and Fachinformationszentrum Karlsruhe via http://www.ccdc.cam.ac.uk/structures.

S4. Catalysis

S4.1. Spectra

Transfer hydrogenation of acetophenone using ReactIR

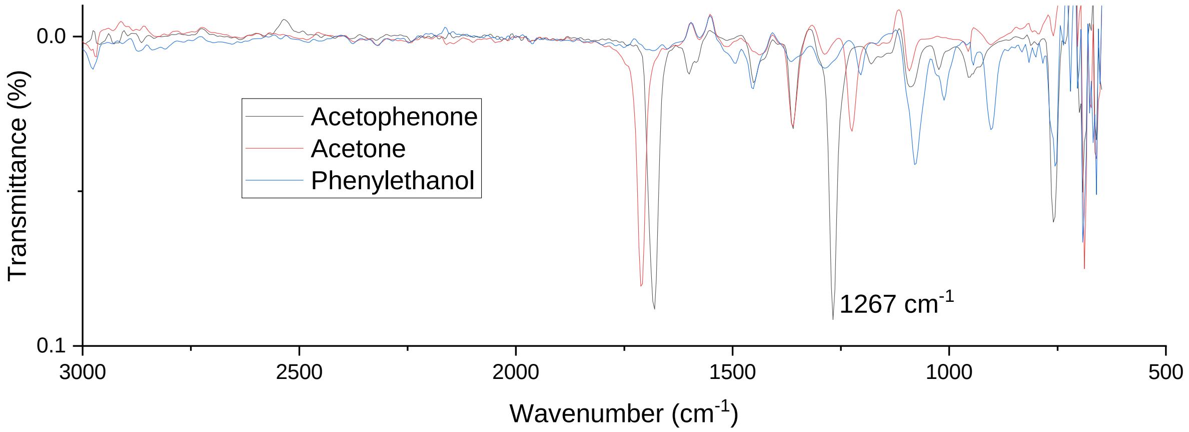

Figure S102. IR spectrum of acetophenone, acetone and 1-phenylethanol. The peak at 1267 cm^{-1} (C_{Ar}–C_{CO}–C_{Me} stretch) was tracked to measure the conversion of acetophenone with time.

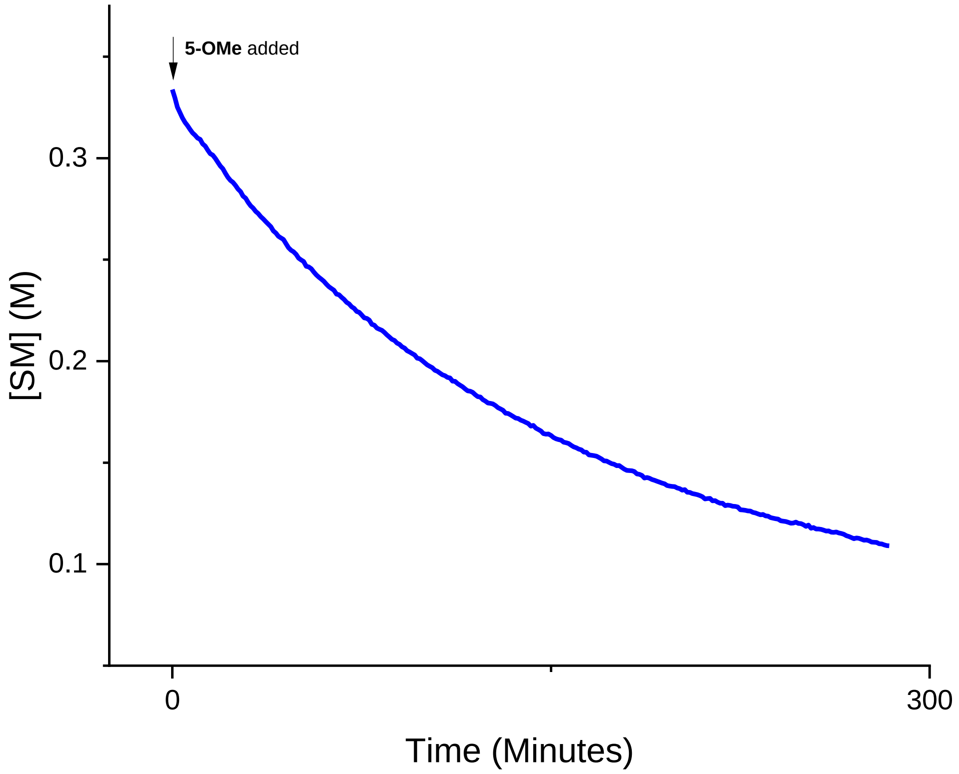

Figure S103. Conversion of acetophenone (SM) with time after addition of **5-OMe** as catalyst.

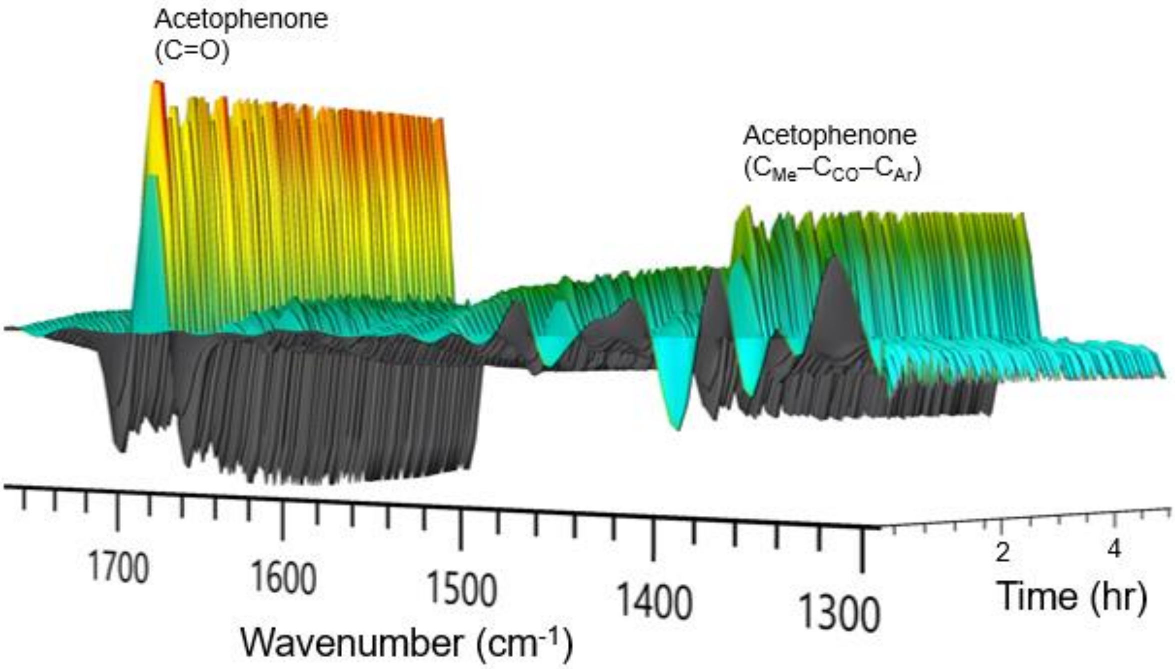

Figure S104. ReactIR surface plot of TH reaction using **4-OMe** as catalyst.

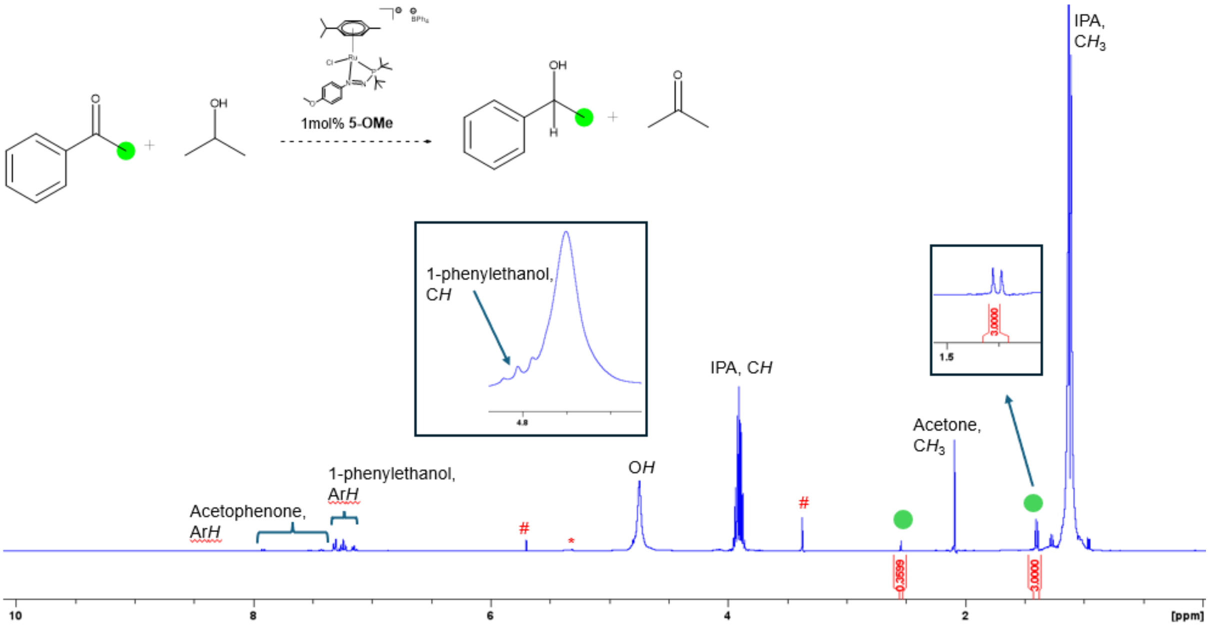

Figure S105. ¹H NMR spectrum of an aliquot of the TH reaction using **5-OMe** as catalyst. * = residual CDHCl2, # = internal standard (trimethoxybenzene in $CDCl_3$ within a sealed capillary tube).

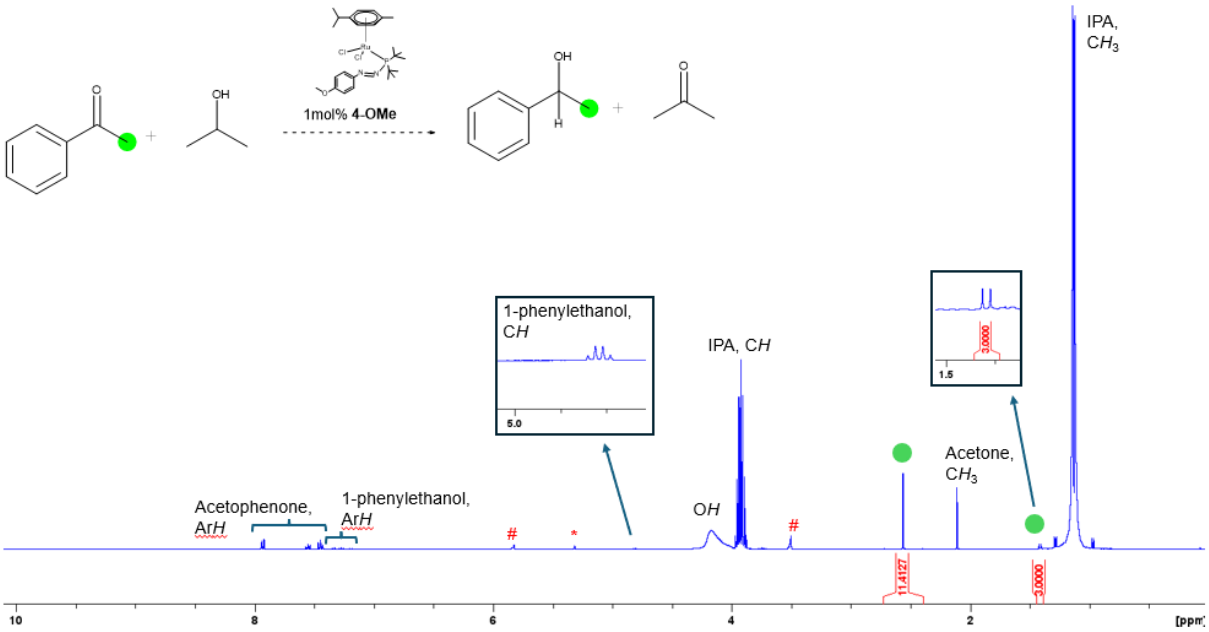

Figure S106. ^{1}H NMR spectrum of an aliquot of the TH reaction using **4-OMe** as catalyst. * = residual CDHCl2, # = internal standard (trimethoxybenzene in $CDCl_3$ within a sealed capillary tube).

Transfer hydrogenation (NMR scale)

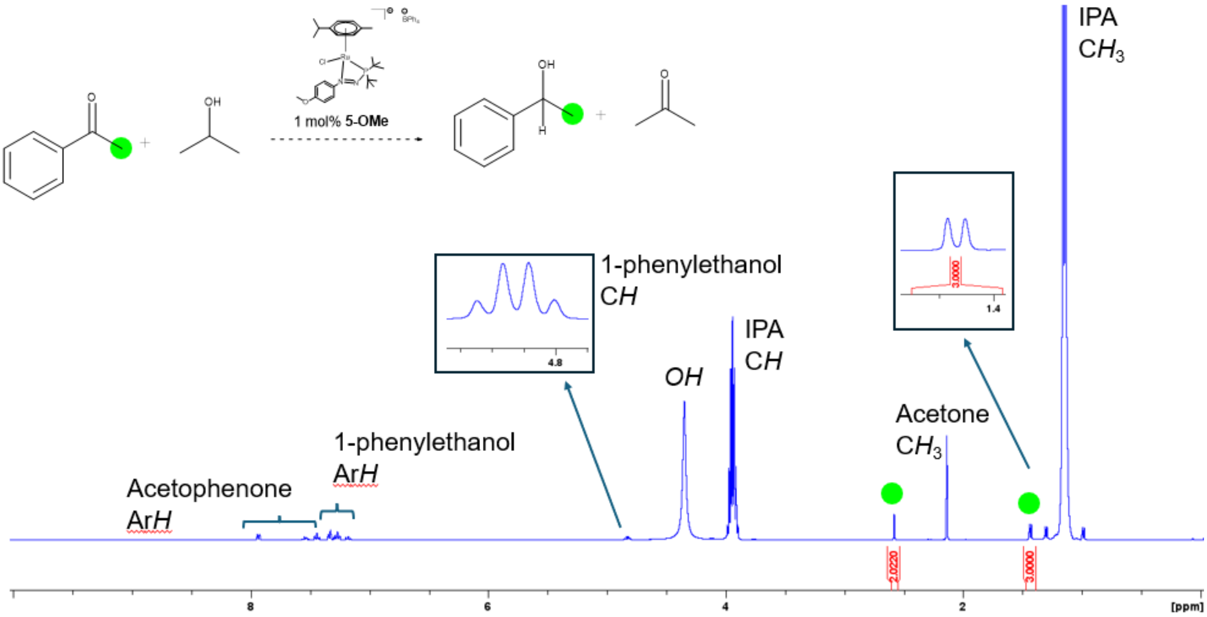

Figure S107. ^{1}H NMR spectrum of an aliquot of the TH of acetophenone using IPA as the proton source and **5-OMe** as catalyst.

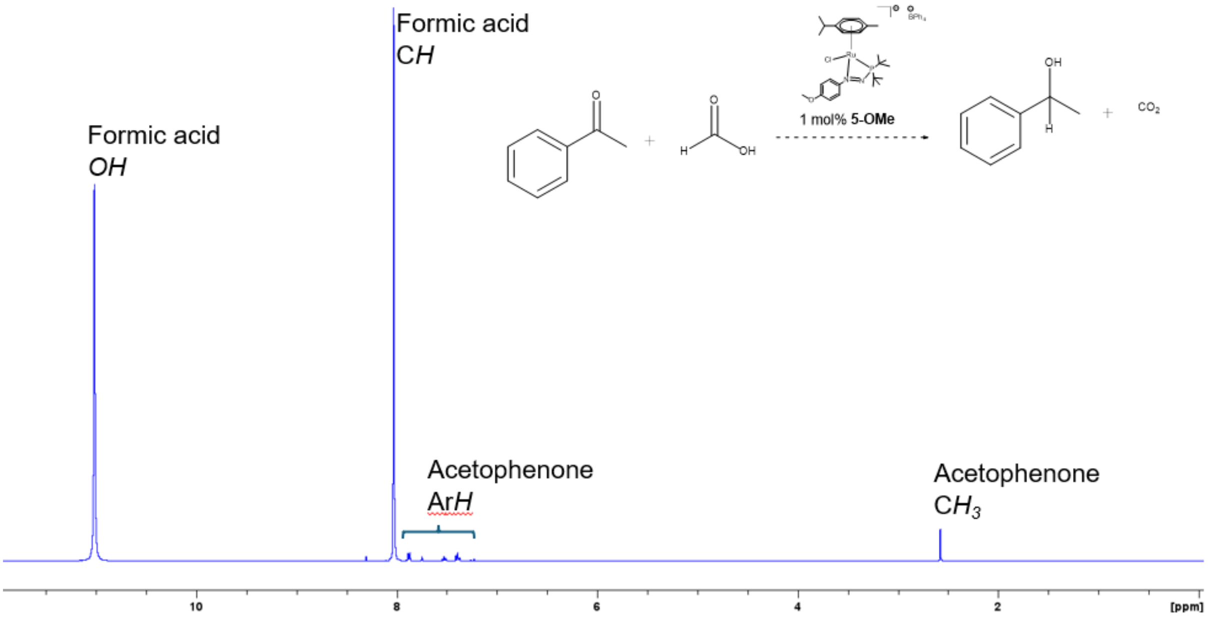

Figure S108. ^{1}H NMR spectrum of an aliquot of the TH of acetophenone using formic acid as the proton source and **5-OMe** as catalyst.

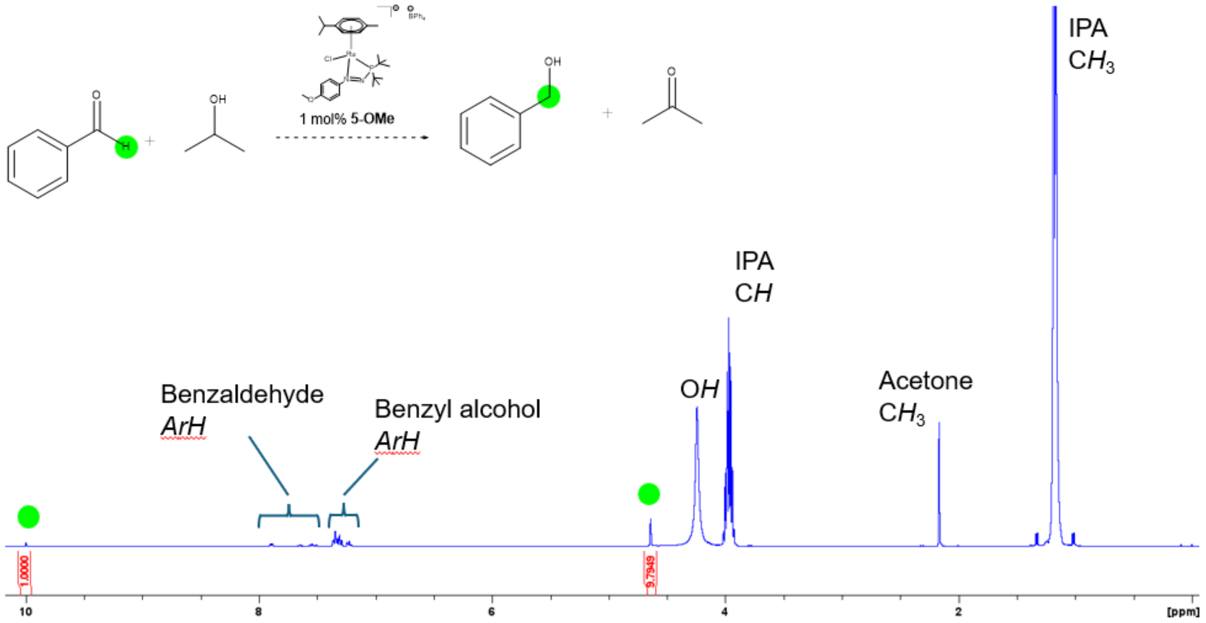

Figure S109. ^{1}H NMR spectrum of an aliquot of the TH of benzaldehyde using IPA as the proton source and **5-OMe** as catalyst.

S5. Computational Details

S5.1. General Information for Optimisations of Free Azophosphines

Calculations for all free azophosphines (**1-R** (R = CF_3, F, H, Me, OMe, NMe_2)) were performed using DFT in Gaussian 09.[10] Geometry optimisations and frequency calculations were carried out using the M06-2X functional and the def2-TZVPP basis set,[11, 12] using Grimme's D3 dispersion correction,[13] solvent interactions modelled using the polarisable continuum model (using toluene as the solvent),[14] and symmetry disabled (keyword = nosymm). Frequency calculations were carried out on all optimised structures; for all geometries, the absence of any imaginary frequencies confirmed that each optimised structure was located at a minimum. NBO calculations (including Wiberg bond indices and NPA analysis) were carried out on optimised geometries using the NBO package in Gaussian 09,[15] using the keywords Pop=(FULL,NBOREAD,SAVENBO). Full cartesian coordinates for all optimised structures can be found in externally, in the file entitled *AJP011.xyz*. The structures of the structures analysed are shown below.

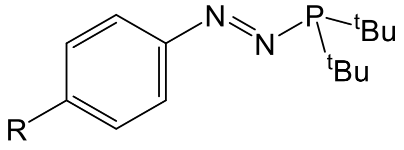

R = CF_3
F
H
Me
OMe
NMe_2

S5.2. Computational Data for Free Azophosphines

Table S3. Pertinent bond lengths for optimised structures.

R =	C-N	N=N	N-P
**CF_3**	1.433	1.236	1.744
**F**	1.430	1.236	1.753
**H**	1.433	1.236	1.753
**Me**	1.431	1.236	1.754
**OMe**	1.427	1.237	1.751
**NMe_2**	1.419	1.239	1.759

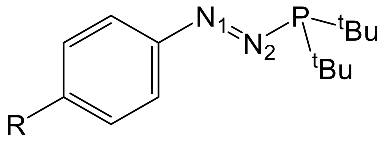

Table S4. NPA (natural population analysis) values for optimised structures.

R =	Nitrogen 1	Nitrogen 2	Phosphorus
**CF_3**	-0.18977	-0.43892	0.99563
**F**	-0.17949	-0.44682	0.98311
**H**	-0.18189	-0.443	0.98237

**Me**	-0.17926	-0.44691	0.98005
**OMe**	-0.17666	-0.4572	0.98047
**NMe_2**	-0.16917	-0.46895	0.97066

Table S5. Donor/acceptor analysis for optimised structures. These values are obtained as printed from the relevant NBO calculation, from the section titled 'SECOND ORDER PERTURBATION THEORY ANALYSIS OF FOCK MATRIX IN NBO BASIS'.

R =	**P lp to N=N π* orbital** (kcal/mol)	**C=C π to N=N π* orbital** (kcal/mol)
**CF_3**	9.18	18.71
**F**	7.33	20.77
**H**	7.25	20.08
**Me**	6.88	20.89
**OMe**	7.1	17.54
**NMe_2**	4.73	24.88

Table S6. Wiberg Bond Indices for optimised structures.

R =	**C–N**	**N=N**	**N–P**
**CF_3**	1.0590	1.8463	0.8836
**F**	1.0644	1.8539	0.8653
**H**	1.0600	1.8574	0.8666
**Me**	1.0639	1.8558	0.8637
**OMe**	1.0717	1.8469	0.8657
**NMe_2**	1.0881	1.8418	0.8480

S5.3. General Information for Optimisations of Ru-Azophosphine Complexes

Calculations for all Ru-azophosphine complexes were performed using DFT in Gaussian 09.[10] Geometry optimisations and frequency calculations were carried out using the ωB97XD functional and the def2-TZVP basis set.[11,12,15] Optimisations were carried out with solvent interactions modelled using the polarisable continuum model (using toluene as the solvent),[14] and symmetry disabled (keyword = nosymm). Frequency calculations were carried out on all optimised structures; for all geometries, the absence of any imaginary frequencies confirmed that each optimised structure was located at a minimum. Single point energy corrections were then carried out on these optimised structures using the ωB97XD functional and def2-TZVPP basis set.[11,12,15] Solvent interactions were modelled using the polarisable continuum model.[14] Both toluene and chlorobenzene as the solvent were used; results with chlorobenzene are used in the main manuscript as these gave significantly smaller energy differences between monodentate and bidentate complexes, as well as chlorobenzene being the solvent used in experimental studies. Results for both solvents are given below. All calculations were performed with symmetry disabled (keyword = nosymm). For computational efficacy, Cl^- is modelled as the counter-ion for all Ru complexes where necessary, rather than the BPh_4^-

counter-ion used in experimental studies. The halide abstracting agent ($NaBPh_4$) and side-product (NaCl) has not been modelled in this study, only the Ru-based complexes. The Cl^- anion and Ru complexes are optimised separately, and the Gibbs energies of each summed to produce a Gibbs energy for the whole complex. Full cartesian coordinates for all optimised structures can be found in externally, in the file entitled *AJP011.xyz*. The structures of the Ru-complexes analysed are shown below.

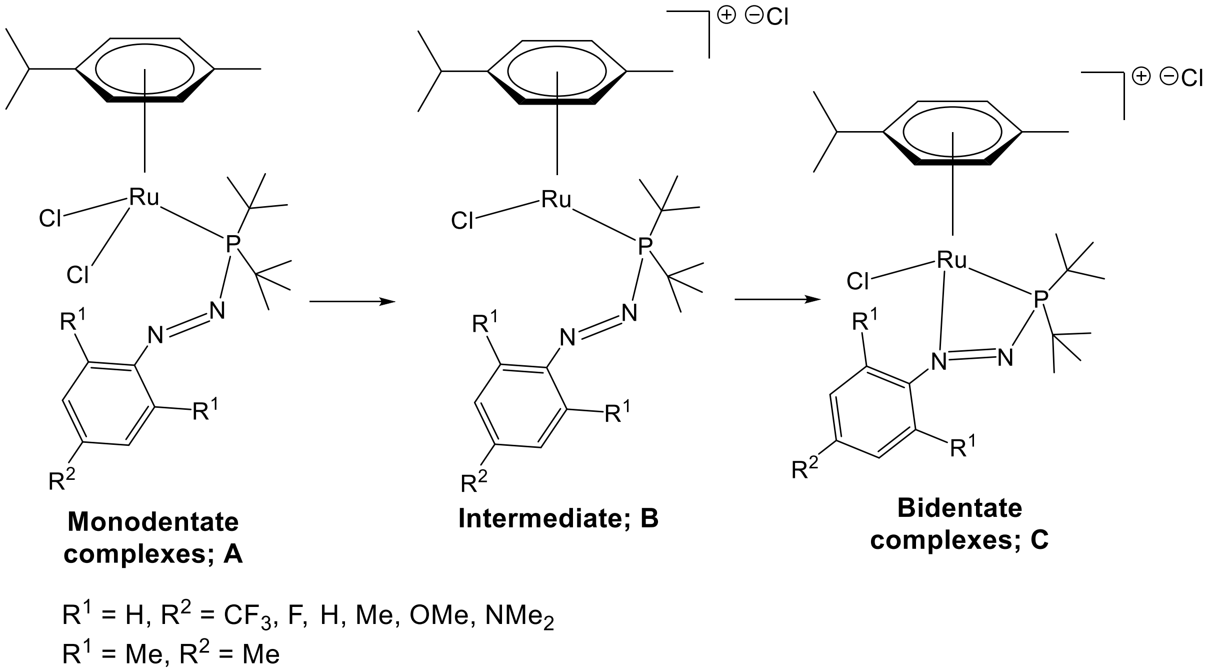

R^1 = H, R^2 = CF_3, F, H, Me, OMe, NMe_2
R^1 = Me, R^2 = Me

S5.4. Computational Data for Ru-Azophosphine Complexes

Table S7. Gibbs energies for monodentate complexes, intermediates, and bidentate complexes, using toluene as the solvent.

R^1	R^2	$Gibbs_{(C)} - Gibbs_{(A)}$ (kcal.mol^{-1})	$Gibbs_{(B)} - Gibbs_{(A)}$ (kcal.mol^{-1})	$Gibbs_{(C)} - Gibbs_{(B)}$ (kcal.mol^{-1})
H	CF_3	45.19	48.55	-3.38
H	F	43.61	48.89	-5.28
H	H	42.56	47.09	-4.53
H	Me	42.20	46.89	-4.69
H	OMe	41.45	45.44	-3.98
H	NMe_2	39.87	46.14	-6.26
Me	Me	54.03	47.54	+6.49

Table S8. Gibbs energies for monodentate complexes, intermediates, and bidentate complexes, using chlorobenzene as the solvent.

R^1	R^2	$Gibbs_{(C)} – Gibbs_{(A)}$ (kcal.mol^{-1})	$Gibbs_{(B)} – Gibbs_{(A)}$ (kcal.mol^{-1})	$Gibbs_{(C)} – Gibbs_{(B)}$ (kcal.mol^{-1})
H	CF_3	20.70	23.97	-3.27
H	F	19.50	24.59	-5.10
H	H	18.71	23.21	-4.50
H	**Me**	18.54	23.22	-4.68
H	OMe	17.95	22.01	-4.06
H	NMe_2	16.90	23.17	-6.26
Me	Me	30.86	23.94	+6.92

References

1. CrysAlisPro, Rigaku Oxford Diffraction, 2021.
2. O. V. Dolomanov, L. J. Bourhis, R. J. Gildea, J. A. K. Howard and H. Puschmann, *J. Appl. Crystallogr.*, 2009, **42**, 339-341.
3. G. M. Sheldrick, *Acta Crystallogr.,* 2015, **A71**, 3-8.
4. G. M. Sheldrick, *Acta Crystallogr.,* 2015, **C71**, 3-8.
5. Hutchinson, G.; Welsh, C. D. M.; Burés, J., *J. Org. Chem.*, 2021, **86**, 2, 2012-2016.
6. Gaussian 09, Revision D.01, M. J. Frisch, G. W. Trucks, H. B. Schlegel, G. E. Scuseria, M. A. Robb, J. R. Cheeseman, G. Scalmani, V. Barone, G. A. Petersson, H. Nakatsuji, X. Li, M. Caricato, A. Marenich, J. Bloino, B. G. Janesko, R. Gomperts, B. Mennucci, H. P. Hratchian, J. V. Ortiz, A. F. Izmaylov, J. L. Sonnenberg, D. Williams-Young, F. Ding, F. Lipparini, F. Egidi, J. Goings, B. Peng, A. Petrone, T. Henderson, D. Ranasinghe, V. G. Zakrzewski, J. Gao, N. Rega, G. Zheng, W. Liang, M. Hada, M. Ehara, K. Toyota, R. Fukuda, J. Hasegawa, M. Ishida, T. Nakajima, Y. Honda, O. Kitao, H. Nakai, T. Vreven, K. Throssell, J. A. Montgomery, Jr., J. E. Peralta, F. Ogliaro, M. Bearpark, J. J. Heyd, E. Brothers, K. N. Kudin, V. N. Staroverov, T. Keith, R. Kobayashi, J. Normand, K. Raghavachari, A. Rendell, J. C. Burant, S. S. Iyengar, J. Tomasi, M. Cossi, J. M. Millam, M. Klene, C. Adamo, R. Cammi, J. W. Ochterski, R. L. Martin, K. Morokuma, O. Farkas, J. B. Foresman, and D. J. Fox, Gaussian, Inc., Wallingford CT, 2016.
7. F. Weigend and R. Ahlrichs, *Phys. Chem. Chem. Phys*., 2005, **7**, 3297-3305.
8. Y. Zhao and D. G. Truhlar, *Theor. Chem. Acc.*, 2008, **120**, 215-241.
9. S. Grimme, *Wiley Interdiscip. Rev.: Comput. Mol. Sci*., 2011, **1**, 211-228.
10. F. Lipparini, G. Scalmani, B. Mennucci, E. Cancès, M. Caricato and M. J. Frisch, *J. Chem. Phys*., 2010, **133**, 014106.
11. NBO Version 3.1, E. D. Glendening, A. E. Reed, J. E. Carpenter, and F. Weinhold.
12. J-D. Chai and M. Head-Gordon, *Phys. Chem. Chem. Phys*., 2008, **10**, 6615-6620
13. M.L. Laury and A.K. Wilson, *J. Chem. Theory Comput.*, 2013, **9**, 3939-3946
